# Dynamic pigmentary and structural coloration within cephalopod chromatophore organs

**DOI:** 10.1038/s41467-019-08891-x

**Published:** 2019-03-01

**Authors:** Thomas L. Williams, Stephen L. Senft, Jingjie Yeo, Francisco J. Martín-Martínez, Alan M. Kuzirian, Camille A. Martin, Christopher W. DiBona, Chun-Teh Chen, Sean R. Dinneen, Hieu T. Nguyen, Conor M. Gomes, Joshua J. C. Rosenthal, Matthew D. MacManes, Feixia Chu, Markus J. Buehler, Roger T. Hanlon, Leila F. Deravi

**Affiliations:** 1 Department of Chemistry and Chemical Biology, Northeastern University, Boston, MA 02115 USA; 2 The Eugene Bell Center, The Marine Biological Laboratory, Woods Hole, MA 02543 USA; 3 Department of Biomedical Engineering, Tufts University, Medford, MA 02155 USA; 4 Laboratory for Atomistic and Molecular Mechanics (LAMM), Department of Civil and Environmental Engineering, Massachusetts Institute of Technology, Cambridge, MA 02139 USA; 5 Institute of High Performance Computing, A*STAR, Singapore, 138632 Singapore; 6 Department of Molecular, Cellular, and Biomedical Sciences, University of New Hampshire, Durham, NH 03824 USA

## Abstract

Chromatophore organs in cephalopod skin are known to produce ultra-fast changes in appearance for camouflage and communication. Light-scattering pigment granules within chromatocytes have been presumed to be the sole source of coloration in these complex organs. We report the discovery of structural coloration emanating in precise register with expanded pigmented chromatocytes. Concurrently, using an annotated squid chromatophore proteome together with microscopy, we identify a likely biochemical component of this reflective coloration as reflectin proteins distributed in sheath cells that envelop each chromatocyte. Additionally, within the chromatocytes, where the pigment resides in nanostructured granules, we find the lens protein Ω- crystallin interfacing tightly with pigment molecules. These findings offer fresh perspectives on the intricate biophotonic interplay between pigmentary and structural coloration elements tightly co-located within the same dynamic flexible organ - a feature that may help inspire the development of new classes of engineered materials that change color and pattern.

## Abstract (web summary)

Chromatophores in cephalopod skin are known for fast changes in coloration due to light-scattering pigment granules. Here, authors demonstrate structural coloration facilitated by reflectin in sheath cells and offer insights into the interplay between structural and pigmentary coloration elements.

## Introduction

The richest and most diverse color patterns in the animal kingdom occur in organisms that have evolved elegant combinations of structural and pigmentary elements to manipulate light efficiently. For instance, octopus, squid, and cuttlefish have the ability to dynamically alter their appearance to quickly display a diverse range of camouflage and signaling^1–4^. This fast and dynamic adaptation involves the use of specialized dermal structures that modulate the animal’s appearance through multiple effects^5^. These structures include pigmentary chromatophore organs uppermost in the dermis as well as two classes of non-pigmentary (structural) coloration cell types: iridocytes that can specularly reflect nearly any color, appearing iridescent; and leucocytes that diffusely reflect all visible wavelengths at once, producing bright white. Iridocytes comprise protein platelets of a high-refractive-index protein—reflectin—that selectively reflect light via thin-film interference, producing a variety of iridescent colors spanning the visible spectrum^6–9^. Leucocytes are also reflectin-based but mostly use microspheres to reflect diffuse white light^10^. Unlike the leucophores and iridophores, the composition of the light-interacting elements in the chromatophore is not yet well characterized.

Given their ability to work as dynamic color-filters within living tissue, the functional morphology of the chromatophore is of considerable interest from the viewpoints of both basic and applied science. Chromatophore neuromuscular organs comprise five cell types: nerves, glial cells, radial muscles, sheath cells, and the large central chromatocyte that is filled with a flexible cytoelastic sac containing nanostructured pigmented granules^11^. In *Doryteuthis pealeii*, each chromatocyte has 18–30 muscles radially arranged around its periphery that, upon neural stimulation, pull the pigmented cell outward into a flat colored disc^12,13^. The mature chromatocyte is occupied almost entirely with nanostructured granules containing ommochrome pigments and associated proteins that may help to maintain spatial cohesion among granules^14–16^. This combination of pigments and proteins maintains the color uniformity and richness of the chromatocytes^17,18^ even as the cell expands into a very thin (2–4 granules thick) layer during actuation. Expansion of these systems is extremely fast (ca. 125 msec) due to the contractions of the radial muscle fibers attached to the chromatocyte. Thus, of particular interest is whether—and to what extent—light is being manipulated by both pigmentary and structural coloration within the cephalopod chromatophore to aid in its optical functions^5,6^.

Although the composition of the pigments in the squid *D. pealeii* chromatophores has recently been verified as a combination of xanthommatin and decarboxylated xanthommatin^15^, the proteins within the chromatophore saccule, specifically those that might coordinate and couple with the pigments to aid in color filtering during actuation, remain unknown. Several proteins have been identified within or near the chromatophores including S-crystallin^14^, reflectin^14^, and r-opsin^19,20^. Crystallins are a diverse set of proteins found in the lens of animal eyes. One isoform, S-crystallin, has been found in cephalopod eyes and skin^21^. This crystallin has also recently been shown to assemble into patchy colloids of varying density and refractive indices to prevent spherical aberration in the eyes of squid^22^. Another cephalopod lens crystallin found in the skin, Ω-crystallin, is structurally homologous to aldehyde dehydrogenase, although it is enzymatically inactive^21,23,24^. It is also the predominant isoform of crystallin found in the bioluminescent light organ of the Hawaiian bobtail squid *Euprymna scolopes*^25^. Although the S- and Ω-isoforms are compositionally dissimilar, they share (i) high-refractive indices, (ii) optical transparency, and (iii) solubility in water, suggesting key functional roles in both the eyes and skin of the animals^21,24,26^. The structural protein reflectin is cephalopod-specific and aggregates into nanoparticles or self-assembles into ribbons^27–29^, contributing to its ability to scatter and reflect light that produces colorful iridescence or diffuse whiteness^30,31^. Rhodopsin, a light-sensitive protein involved in phototransduction, has also been implicated in skin optical function, including the ability of the (octopus) chromatophore system to respond to light even when physically isolated from cephalic input^9,19,20^.

In this work, we present evidence of an additional optical feature of cephalopod skin: intense colorful reflection from chromatophores. When combined with a systematic compositional and computational study, our data reveal that these organs are a remarkably refined system that produces dynamic coloration, stimulating reassessment of (i) how cephalopods are able to produce structural color in the absence of iridophore-like plates, and more generally (ii) how light might be manipulated to produce both pigmentary and structural coloration with richer photonic repertoires than previously recognized.

## Results

### Expanded chromatophores exhibit intense structural color

**Fig. 1:**
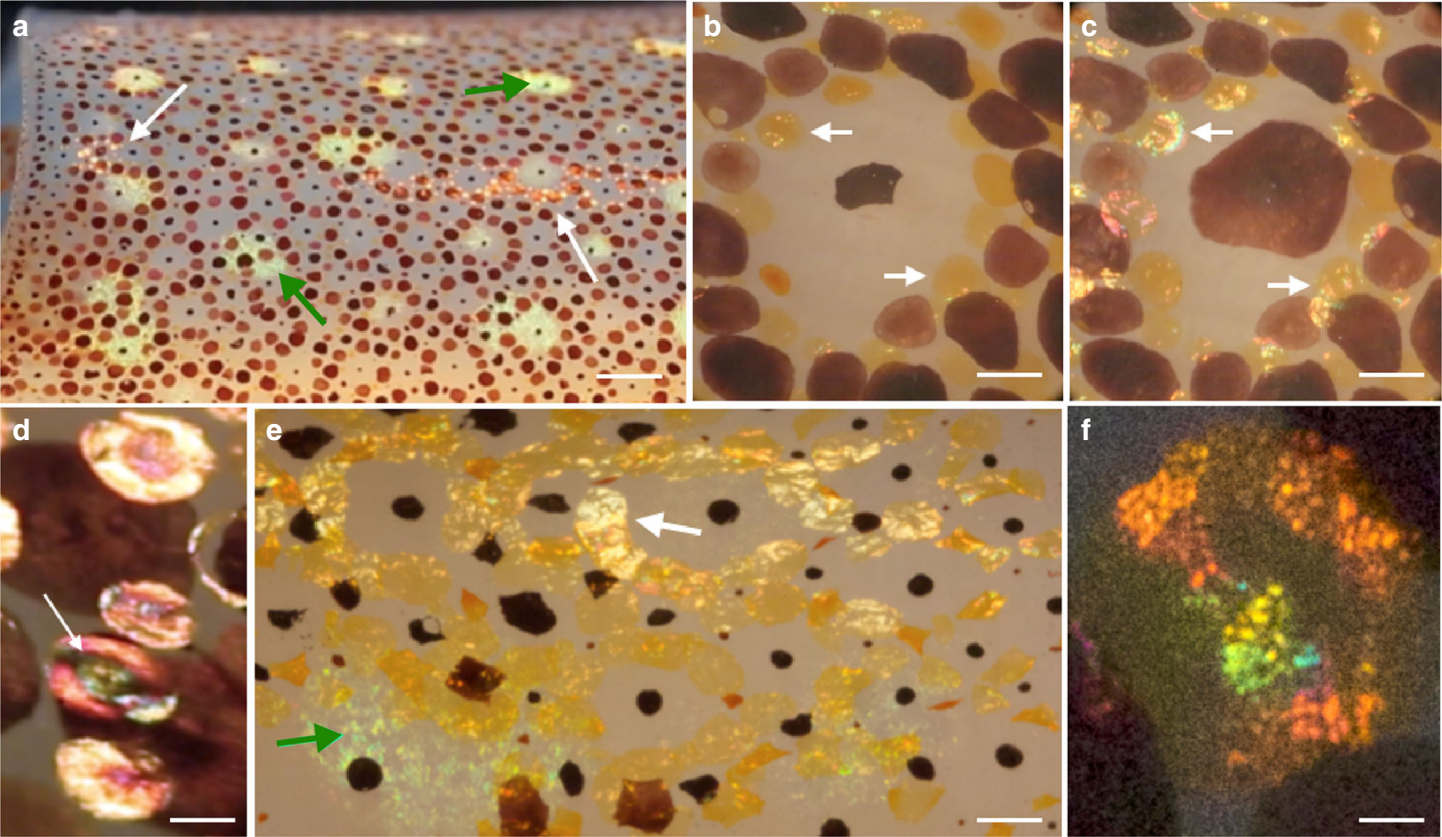
Iridescence produced in chromatophores of live squid. **a** Low power of mantle skin; arrows indicate narrow zones of iridescence that coincide with yellow chromatocytes (white arrows) and typical iridophores (green arrows). Scale bar is 3 mm. **b**, **c** Arrows show yellow chromatophores under different lighting angles expressing different iridescence. Scale bars are 600 µm. **d** Yellow chromatophores showing iridescence that coincides with the exact expansion of the chromatocyte (arrow shows concentric variation in hue seen frequently). Scale bar is 300 µm. **e** Contrasting iridescence from a typical subjacent iridophore (green arrow) compared to a yellow chromatophore (white arrow). Scale bar is 1 mm. **f** A single yellow chromatophore showing granular and patchy iridescence. Scale bar is 100 µm

We imaged chromatophores in living squid and in excised viable skin preparations using bright-field optical microscopy and observed colorful iridescent patches located precisely across the expanded surface of every color type of chromatophore (yellow, red, or brown), especially with incident light at ca. 20–50° from the viewing angle (Fig. 1). At the whole-animal level, we observed narrow streaks or patches of iridescence especially along the mantle and head of the squid (Fig. 1a and Supplementary Movie 1). All three colors of chromatophores displayed this iridescence (Supplementary Movie 2), but particularly intense and colorful reflection was observed from the yellow chromatophores in live intact skin (Fig. 1b–e and Supplementary Movie 3). At high magnification, this phenomenon showed an iridescent, multiple-hue structural coloration quality even across a single chromatophore. The colors progressed sequentially akin to Newton’s series (orders two and three, i.e., purple, blue, green, yellow, orange, red, Fig. 1f and Supplementary Movie 4)^32^. At times the reflectance appeared as a wrinkled membranous material (Fig. 1e and Supplementary Movie 3), reminiscent of shrink-wrapped plastic deforming under tension.

This reflectivity observed from the actuating chromatophores was initially mistaken for the iridescence long-known to be produced by underlying iridocytes (Fig. 1a and e, green arrow). However, upon closer examination we noted that the iridescence observed from the chromatophores was certainly different in location, size, shape, texture, and timing from the subjacent iridescing iridocytes. In *D. pealeii*, clusters of iridocytes sit below the uppermost chromatophore layer of the skin, where they could be readily recognized under the dissecting microscope from other cell types. Squid skin also contained large iridophore patches (several mm across, containing hundreds of iridocytes) that were located deeper in the dermis, widely spaced and at much lower densities than the chromatophores (Fig. 1a–e, and Supplementary Movie 1). Moreover, iridophore iridescence exhibited minute facets, each reflecting a dominant wavelength (based on numerous oriented platelets, operating singly and as Bragg stacks). Their hue did not vary with changing illumination angle like that observed from the iridescing yellow chromatophores (Fig. 1 and Supplementary Movies 2, 3, 4).

**Fig. 2:**
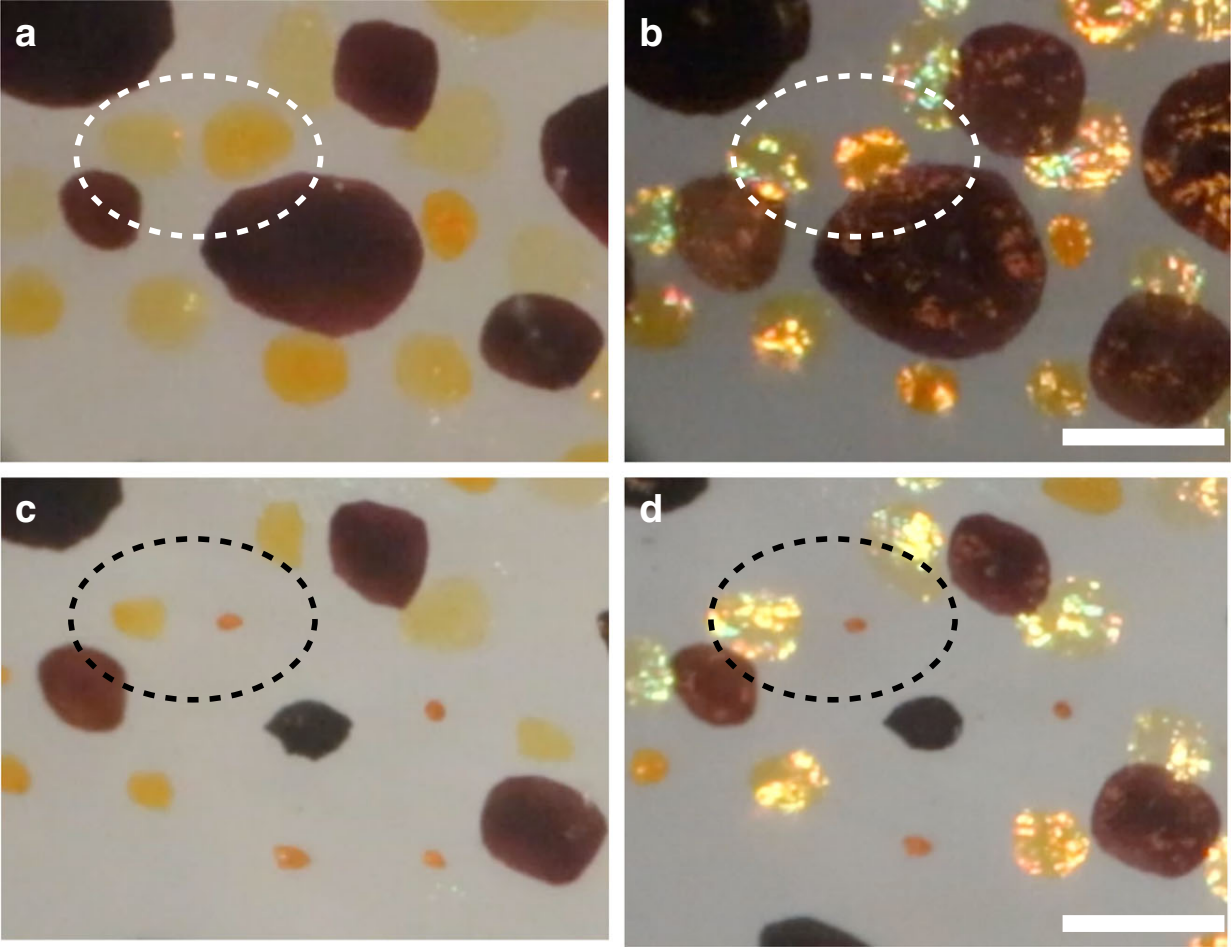
Specular reflection from chromatophores. Still images collected from dynamic Movies of intact adult squid skin through a dissecting microscope using a movable light-pipe illuminator showing that specular reflection from chromatophores requires both specific illumination conditions and expansion of the chromatophores. **a** A subset of chromatophores (white dotted circles) in expanded configurations with off-axis illumination, where perceived color is attributed to pigment absorption and scattering; and **b** moments later, the same expanded chromatophores showing strong specular reflections (interpreted as structural coloration) that are seen restricted precisely to regions within each chromatophore. Only the angle of illumination was changed. When the same chromatophores are captured before **c** and after **d** actuation (black dotted circles), coloration varies depending on the angle of illumination, where there is intense specular reflectance for the expanded chromatophores but not for the compacted ones. (Movie frame time points all in seconds are as follows: *A* = 35 and *B* = 38, *C* = 4, and *D* = 10). Scale bars are 1 mm

In many parts of the squid mantle, chromatophores exhibited morphological discoid units^33^, with one large central brown chromatophore ringed by circlets of smaller yellow, red, and brown ones (Fig. 1a–e). With incident light at ca. 20–50 degrees from the viewing angle, these regions showed a specular golden reflectance perfectly coincident with each yellow chromatocyte (Fig. 1e). The optical clarity of living skin permitted close examination of these and many other chromatophores, providing strong confidence that the structural coloration effect reported here emanated strictly from the chromatophores. Most conclusively, however, the reflective color was coextensive with the dynamic shape of each chromatophore (Fig. 1d, e and Supplementary Movies 2, 3). The iridescence expanded and reduced in precise register and sub-second timing with the changing diameter of the pigment-containing chromatocyte, whether actuated naturally, biochemically, or by electrical stimulation (Supplementary Movie 5). The iridescence depended on the orientation of the incident light (Fig. 2a-b) and on the state of expansion of the chromatophore (Fig. 2c-d).

**Fig. 3:**
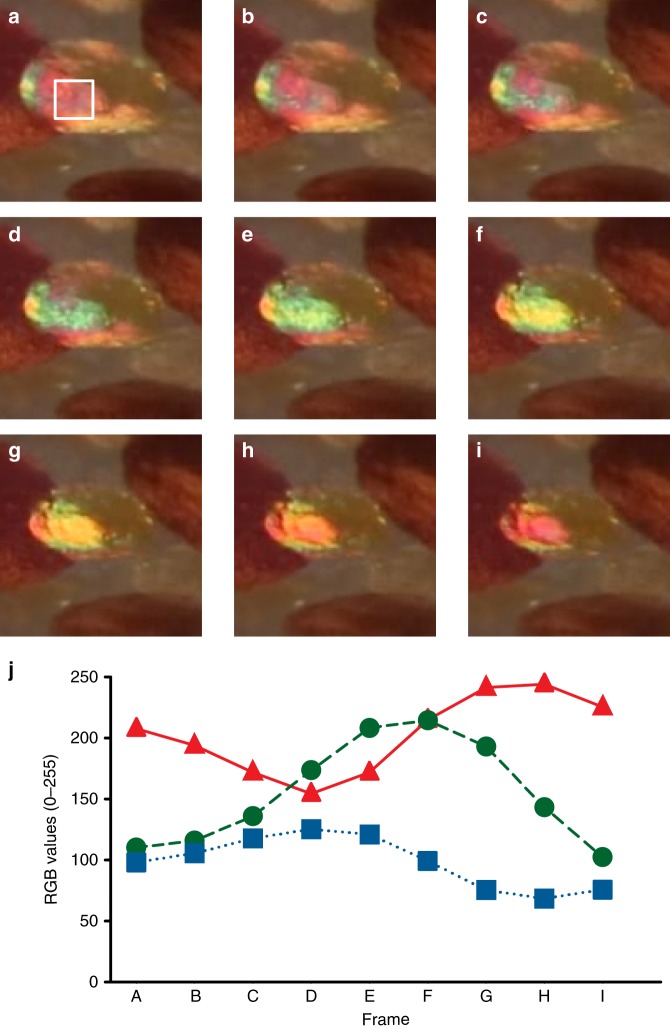
Dynamic visible color emanating from a single chromatophore over time Consecutive Movie frames (**a**–**i**) taken from Supplementary Movie 2 (7.60–7.87 s, where each frame represents 0.03 s) showing the relaxation of a yellow chromatophore in the lower right-hand side. The white outline in **a** (20 × 20 pixel square) shows the location of the selected area used to measure RGB profiles in each frame. These frames were selected because they encompass the visible spectrum, giving unique red, green, and blue (RGB) values during the actuation cycle. **j** Using ImageJ software, the RGB values over the selected area were measured, averaged, and plotted from each frame, Here, the line graph shows the changes in RGB value intensities over time with the red triangles anchored by solid red lines representing red values, the green circles anchored by dashed green lines representing green values, and the blue squares anchored by dotted blue lines representing blue values. The changing RGB values shown here comprise a much wider range of colors than previously attributable to absorbance by any yellow chromatophores and constitute strong argument that dynamic structural color mechanisms must be present

These findings are completely different from the electrical and chemical induced changes in iridophore coloration, which are known to require seconds or minutes^9^. Many of the iridescing chromatophores exhibited variations in their reflected color centrally versus peripherally, possibly related to differences in chromatophore thickness (Fig. 1d). In addition, at higher magnification, the observed iridescence sometimes appeared granular (Fig. 1f), suggesting a possible role for subcellular structures in or near the chromatophore in creating this phenomenon. In all cases, the color reflected from actuating chromatophores was confined to the area of the saccule, displaying a dynamic range that spanned the visible spectrum (Fig. 3a-j).

### Compositional analysis of the squid chromatophore

**Fig. 4:**
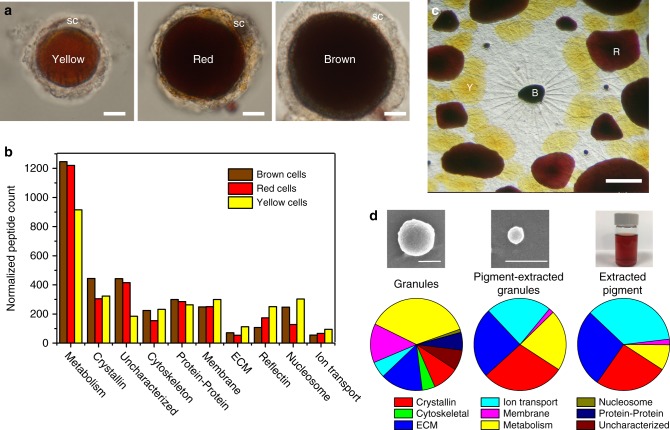
Spectrometric analysis of chromatophore proteins. **a** Enzymatically isolated squid dorsal mantle chromatophores, retaining the plasma membrane of the variously colored chromatocytes and their surrounding sheath cells (sc). Scale bar is 6.5 µm. **b** Proteins identified and categorized by function in the yellow, red, and brown pigment cells in squid *D. pealeii d*orsal and ventral mantle. **c** Typical morphological arrangement of chromatophores in squid skin; note the radial muscles visible around the central retracted brown chromatophore. Scale bar is 500 µm. **d** Categorized proteins identified within the squid chromatophore granules, pigment-extracted granules (e.g., the granule shell), and the extracted pigment along with representative SEMs (scale bar = 500 nm) and optical image of extracted pigment

We previously identified an abundance of both S-crystallins and various reflectin isoforms associated with semi-purified granules from cuttlefish chromatophores, suggesting that the granules were more than just chromogenic pigments^14^. However, the relationship between the pigments and proteins in chromatophores, including whether and how they coordinate and couple together to give rise to iridescence, remained unknown. We embarked on a detailed compositional analysis by first enzymatically isolating then manually sorting individual chromatophores by color from the dorsal mantle of five *D. pealeii* individuals, yielding ~700 yellow (*n* = 2 animals), ~700 red (*n* = 2 animals), and ~1000 brown chromatophore organs (*n* = 3 animals; Fig. 4a). The chromatophores were pooled by color, and proteins extracted from each set were proteolyzed and analyzed using tandem mass spectrometry in conjunction with liquid chromatography (LC-MS/MS, Fig. 4b). Bioinformatic analysis of the LC-MS/MS data led to the identification of 469 putative protein entries translated from the transcriptome of squid *D. pealeii* chromatophores. The isolated material included chromatocytes with their enclosed saccule containing the pigment granules, as well as surrounding membranes and, likely, sheath cells that may still be bound to some of the surrounding muscle fibers (Fig. 4c). The function of identified protein entries was annotated through sequence homology using blast against the Uniprot non-redundant protein database. The identity of 412 (all but 57) of these proteins matched positively to known proteins, constituting an annotated proteome specific to squid chromatophores (Table S1). We compared the relative amounts of each identified protein in different color chromatophore cells using a semi-quantitative spectrum counting approach^34,35^ and grouped them by biological functions (Fig. 4b). We assessed the relative abundance of proteins from different color chromatophore cells by a protein abundance index, where peptide counts were scaled by the number of predicted peptides produced by tryptic digestion^36,37^, as detailed in the methods section. Total peptide count was normalized between chromatophore types to avoid sample-loading bias. Owing to the difficulty of collecting sufficient material for replicate experiments, our assessment of relative protein abundances is less accurate for the low abundance proteins^38^.

The most abundant individual protein was Ω-crystallin, whose normalized peptide count was 290, 170, and 256 for the yellow, red, and brown cells, respectively, while S-crystallin was the second most abundant protein in red and brown cells, with normalized peptide counts of 101 and 144. Compared to the total number of proteins, the various crystallin isoforms made up only 10–13% of all peptides detected, while the various metabolism-related proteins, loosely characterized as any protein involved in the production or catabolism of biomolecules, collectively represented 30–40% of the detected peptides (Fig. 4b and Table S1). We observed color-linked variations in nearly every category of protein and two major trends emerged from our analysis (Supplementary Fig. S1): the brightest chromatophores, the yellows, had the most reflectins (isoforms A1, A2, B1, C1, and 3), which have been known to contribute to enhanced reflectivity of surfaces^7,10^; while the darkest chromatophores, the browns, had more crystallin (Ω- plus S-), the latter of which has been suggested to enhance absorption of light via scattering in other systems^39^. The protein content in the red cells represented a combination of the browns and yellows. It is noteworthy that only four proteins were identified as glutathione S-transferases (GSTs) (Table S1). While S-crystallins and GSTs are homologous to one another with similar primary structures^21^, BLASTP comparisons indicated that they can be clearly differentiated from one another. In our categorization, we labeled S-crystallins as such when the identified polypeptide sequence had > 90% coverage and > 60% identity similar to that of S-crystallin (see Table S1). When taken together, the presence of these various protein classes indicated that the sacculus and the surrounding cell membrane network were composed of a heterogeneous population of proteins responsible for signaling, structural, metabolic, and optical functions that contribute to the complexity of the chromatophore organ.

### Crystallin but not reflectin found amidst pigment granules

The presence of reflectins and crystallins in the isolated chromatophores is significant (Fig. S1). It confirms a previous finding of both proteins in *S. officinalis* brown chromatophores^14^ and indicates that the percentage of reflectin varies among the different color types of chromatophores. However, it was still unclear whether the proteins were localized within or outside the chromatocytes or their saccules. Thus, we attempted to target a more selective sub-section of the chromatocyte: the isolated and purified pigment granules. Here, we did not separate the granules based on chromatophore color; instead, we collected whole skin sections across the dorsal and ventral regions of four animals, similar to previous reports^15^. Our extraction protocol also enabled us to selectively extract the pigment from the granules, leaving behind a granule shell that was colorless but still appeared spherical under scanning electron microscopy (Fig. 4d).

We compared the compositions of the intact granules (Table S2), pigment-extracted granules (the granule shell, Table S3), and the extracted pigment (Table S4), where we posited that the remaining shell was composed of proteins that remained largely unaffected by the acid hydrolysis used to extract the pigment. We assessed the relative abundance of identified proteins within each sample using spectral counting, similar to how we treated the chromatophore LC-MS/MS data (Fig. 4d). While peptide count was scaled by the expected number of fragments produced by tryptic digestion, overall protein loading between the samples was not normalized. Based on protein identity, they were either classified by function, as shown in Fig. 4d, or as a contaminant including skin-surface bacterial ribosomal proteins or enzymes used in sample preparation (Table S2–4). After accounting for contaminating proteins, we identified 65, 11, and 8 individual proteins in the granules, pigment-extracted granules, and the pigment-only samples, respectively. The intact granules comprise a variety of proteins associated with metabolism, the cell membrane, and the extracellular matrix, while the shell portion of the granules and the extracted pigment contain a subset of the granule-associated proteins and were both comparatively enriched with Ω-crystallin. S-crystallin and reflectin were not found in any of these three sample types.

### Reflectin molecules are distributed throughout sheath cells

**Fig. 5:**
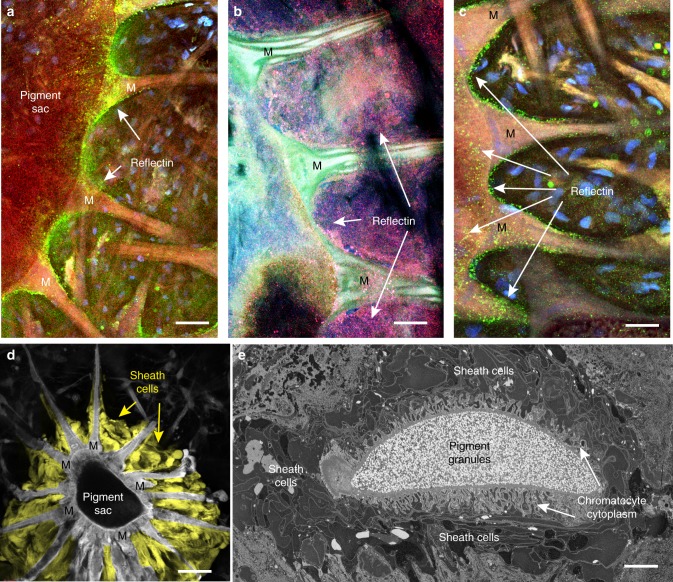
Anatomical localization of reflectin to sheath cells. **a** Reflectin (confocal, secondary antibody in green, arrows) was present surrounding the edge of the chromatocyte and the apical portions of the radial muscles (M). Scale bar is 50 µm; **b** Reflectin (confocal, dual secondary antibodies in lavender; 405 nm and 568 nm fluorophores were applied simultaneously to reduce the potential ambiguity associated with autofluorescence of the tissue) often extended into the spaces between adjacent apical radial muscles. Scale bar is 25 µm; **c** Reflectin distribution was frequently punctate, and in addition to coating the muscles and the edge of the chromatocyte, reflectin was also present over its surface. Note that the chromatocyte section was very thin (in comparison with the confocal depth of field of the 0.45 NA x20 objective used here), but the label consistently showed on its outside, rather than within its sacculus. Scale bar is 25 µm; **d** Confocal section of a chromatophore imaged by autofluorescence with the sheath cells highlighted in yellow for better visualization (scale bar is 50 µm). Sheath cells completely enveloped the pigment sac in all dimensions, as illustrated in the electron micrograph in **e**; scale bar is 12 µm

Our proteomic data indicated that the granule-only samples included Ω-crystallin but not reflectin. This observation was perplexing considering that the whole-cell data in Fig. 4b and Table S1 showed multiple reflectin isoforms present in the manually selected chromatophore material. Thus, we asked whether we could identify where reflectin(s) might reside within the overall chromatophore organ. We obtained polyclonal antibodies that, as confirmed by Western blots, targeted the reflectin A1 and A2 isoforms (other isoforms are known to exist, but specific antibodies against them are not yet available). We used immunocytochemistry and confocal microscopy to identify the cellular distribution of these epitopes in squid skin and observed the strongest signal of reflectin within the sheath cells, which completely envelop the chromatocyte in all planes (Fig. 5a–c), including particularly dense label seen as a mass on the flat surface of the chromatophore (Supplementary Fig. S2). Reflectin was found consistently localized to the sheath cells in chromatophores from both mantle and fin areas of the squid across replicate trials, tissue samples, and chromatophore color. Occasionally, but not routinely, fluorescent signal was observed close to the sacculus and radial muscle fibers.

Sheath cells are an integral component of chromatophore organs, yet their function has not been studied. Historically they were presumed to provide a flexible buffer zone separating the pigment saccule from the adjacent connective tissue, providing low resistance against actuation^11,40^. While no definitive individual morphology has yet been assigned to these cells, our own light and electron micrographs confirmed that sheath cells are hpy ighly folded membranous cells that wrap around the entire chromatocyte with multiple layers, including the proximal portion of the radial muscles near the pigment sacculus potentially also playing a nutritive role, since no mitochondria or other metabolic machinery was seen in our electron micrographs of chromatocytes (Fig. 5d–e and Supplementary Fig. S3A, B). While the total number of cells that ensheath a chromatocyte remains unknown, stains with 4′,6-diamidino-2-phenylindole (DAPI) revealed many nuclei adjacent to the positively stained reflectin regions, which were also adjacent to the chromatocyte and in between the radial muscles (Fig. 5a–c and Supplementary Fig. S2A, C–E). Some of our confocal and electron microscopy images indicate that these cells may be flattened and highly infolded sheets with variable electron densities and textures, suggesting a structural role that could contribute to the regional variation of the chromatophore iridescence (Fig. 5e and Supplementary Fig. S3A, B).

One of the most interesting aspects of this system is that structural coloration is observed without clear underlying ultrastructural electron-dense reflectin-filled lamellae similar to the signature plate-like stacks of cephalopod iridocytes^7,41^. However, thin-film interference in general is known to generate iridescence^42^. Thus, to better understand the potential source(s) of the reflected coloration localized to the chromatophores, we used a multilayer interference equation^43^ to model the dependence of visible color on the nanolayered elements (e.g., sheath cell membranes and cytoplasm) surrounding the chromatophore, where we hypothesized that these structures may contribute to the perceived iridescence. To numerically evaluate this possibility, we measured and averaged the height of 292 cytoplasm layers (*d*_a_ as 116 ± 102 nm) and 237 sheath cell membrane layers (distances, *d*_b_, as 71 ± 14 nm) selected randomly from around the chromatocyte in Fig. S3A (data presented in Fig. S3C; error is reported as standard deviation). We calculated a range of reflected color associated with the variable cytoplasm layer heights (*d*_a_), which were up to ~3x larger than the sheath cell membrane layers. Lower wavelengths were predicted for the smaller spacings (approaching ~100 nm), suggesting that as the chromatophore expands during actuation, the cytoplasm layer would likely spread out, decreasing its layer height to lead to a blue-shifted reflected color (Fig. S3C). As the chromatophore relaxes (e.g., retracts in diameter), the cytoplasm layers thicken back up, effectively increasing the layer thicknesses towards higher wavelengths. Altogether, these estimations provide one potential mechanism describing the dynamic range of visible color presented in the chromatophores during actuation. Dynamic morphology of other components in the chromatophore where refractive index contrast may be present (such as interfaces between sheath cell and chromatocyte or between the sacculus and contained granules) in principle could generate similar optical effects.

### Coordination of pigments and proteins in the granules

Considering that crystallin is specific to the chromatophore granules, while reflectin is localized to the sheath cells along the chromatocyte, we hypothesized that Ω-crystallin might function to stabilize xanthommatin (Xa), the predominant pigment observed within the chromatophore organ^15^, and assist in color retention. To test this, we first evaluated the relative binding affinities of these proteins to Xa. As the crystal structures of the most abundant isoform of each protein in our samples—Ω-crystallin and reflectin A2—were not known, we first optimized structural homologs of each isoform via homology modeling. The structures obtained by these means were further refined with replica exchange molecular dynamics (REMD) simulations in an explicit solvent system (Supplementary Fig. S4). As predicted by I-TASSER^44^ and described experimentally by others^45^, Ω-crystallin was structurally highly related to aldehyde dehydrogenases in animals and bacteria. These observations were supported by our data, showing high average normalized *Z*-score of 4.37 ± 3.74 and sequence identity of 44 ± 9% for the top ten threading templates, as well as high average template modeling (TM) score of 0.91 ± 0.01 and sequence identity of 48 ± 12% for the top ten structural analogs (Tables S5 and 6 error is reported as standard deviation). Even though the functional implications of this similarity to aldehyde dehydrogenases were not clear, our homology models confirmed that Ω-crystallin’s structure was strongly related to those of aldehyde dehydrogenases, resulting in a globular tertiary structure with catalytic and co-factor domains rich in α-helices and β-sheets as well as a characteristic oligomerization tail domain that consisted of β-sheets. As a comparator, we also chose to evaluate Xa’s affinity to reflectin; however, proteins that were structurally related to reflectin were not as numerous as Ω-crystallin due to low sequence identity (12 ± 2% for threading templates and 5 ± 1% for structural analogs; for top ten templates and analogs, where error is reported as standard deviation) (Tables 7–8). Still, we used REMD sampling and showed that the reflectin monomer was highly dominated by α-helical motifs and unstructured linker regions, suggesting a protein structure more flexible and less specific to Xa than Ω-crystallin.

**Fig. 6:**
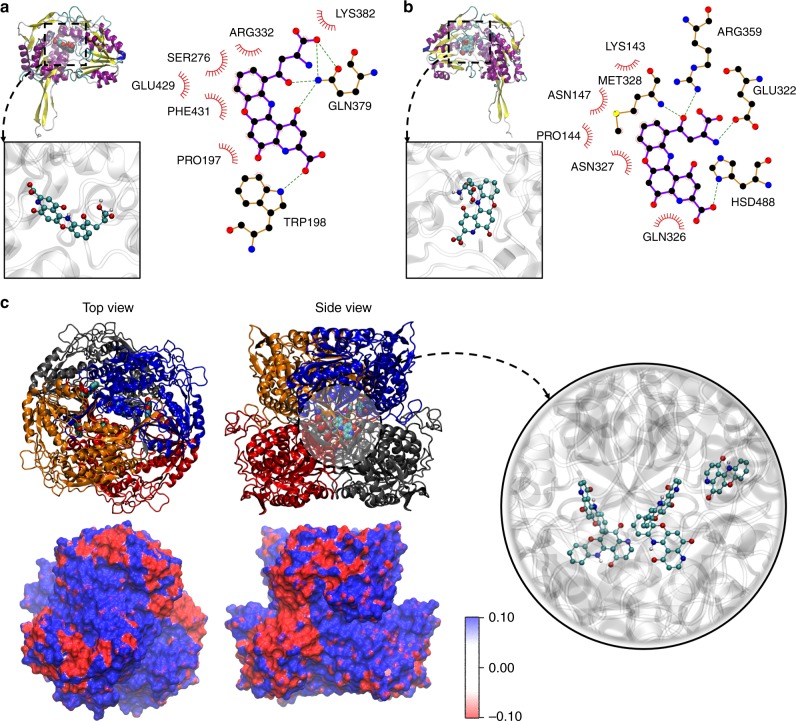
Binding poses elucidated from molecular docking. **a**, **b** Most energetically favorable binding poses from flexible molecular docking of Xa in two of the top-ranked binding pockets in crystallin. In each panel, the figures on the left show the orientation and size of the pigment molecule (space-filling beads) within the binding pockets of the respective proteins (cartoons colored by secondary structure). The inset provides closeup views of the molecule within the pockets. The figures on the right are schematics of the pigment molecule interacting with the amino acid sidechains in the respective pockets. Dotted lines indicate polar interactions between acceptor–donor pairs. Eyelashes (in red) denote sidechains and atoms that are in contact with each other. Solid circles represent carbon (black), oxygen (red), nitrogen (blue), and sulfur (yellow). **c** Top and side views of the five top-ranked poses of Xa binding from rigid docking (space-filling beads) within the central cavity of the crystallin tetramer (cartoon colored by chain number), where the five molecules are in disparate regions of the cavity. Inset provides a closeup view of these poses. Top and side views of the tetramer’s molecular electrostatic potential map are shown at the bottom

We next used rigid docking of the chromatophore pigment Xa to the REMD-deduced protein structures (Fig. 6a, b and Supplementary Fig. S4). The chemical structure of Xa was previously optimized using density functional theory (DFT) methods (see supporting information for details). In Ω-crystallin, we identified two major pigment binding pockets on the left and right regions between the catalytic and co-factor domains of aldehyde dehydrogenases^46,47^, whereas in reflectin we identified two pigment pockets with superficial interactions. Flexible docking of Xa within these pockets was then optimized to calculate the protein-pigment binding energies. The values obtained from these calculations were −10.9 kcal mol^−1^ and −10.0 kcal mol^−1^ for the Xa-bound Ω-crystallin system and −9.5 kcal mol^−1^ for Xa-bound reflectin, indicating that Xa had a more favorable interaction with Ω-crystallin than reflectin (Table 1, Fig. 6a, b, and Supplementary Fig. S5). Comparable differences have been reported in other protein systems to establish significance in binding energetics. For example, a calculated binding energy difference of 0.7 kcal mol^−1^ has been used to distinguish whether minocycline bound to the extrusion or binding protomers of the AcrB protein^48^, while differences ranging from 0.5 to 2.0 kcal mol^−1^ have been reported for other virtual screened ligand-binding complexes to establish significance^49–52^. To further support the significance of our calculated binding energy differences, we performed additional docking calculations on both Ω-crystallin and reflectin using a range of small, aromatic derivatives of Xa, including kynurenine, xanthopterin, xanthurenic acid, cyanidin, tryptophan, and phenoxazine (Table S9). The calculated binding energies of each compound with the two proteins were all higher than Xa, where the smaller molecules (kynurenine, xanthopterin, xanthurenic acid) presented the highest relative binding energies, while the larger molecules (cyanidin, tryptophan, phenoxazine) presented the lowest of the group. Despite these variations, each compound consistently docked with a lower binding energy (i.e. a greater affinity) to crystallin than reflectin with clear energy differences of ~ −1 kcal mol^−1^. This trend further supported our original findings for Xa, which similarly indicated a better binding affinity to Ω-crystallin than reflectin. This higher affinity is likely due to more intermolecular contacts with the amino acid sidechains (19 and 20 for crystallin vs. 15 and 17 for reflectin) between Xa and the available binding pockets of each protein, leading to a concomitant increase in polar interactions and π-π stacking with aromatic sidechains in Ω-crystallin (Fig. 6a, b). Conversely, we observed a weak interaction between Xa and monomeric reflectin (Supplementary Fig. S5) and believe this interaction would likely be further destabilized due to the transient nature of the reflectin secondary structure^53,54^, which has a propensity to aggregate to form oligomeric structures that are rich in β-sheets^27^.

**Table 1:** Calculated binding energies of Xa with chromatophore proteins

Binding energies (kcal mol^−1^)		
	Pocket 1	Pocket 2
Crystallin	−10.0	−10.9
Reflectin	−9.5	−9.5
Average in crystallin tetramer (*N* = 5, ± SD)	−10.7 ± 0.2	

Given Ω-crystallin’s excellent structural homology to aldehyde dehydrogenases and the fact that the experimental crystal structures in both the threading templates and structural analogs were all tetrameric, we investigated how tetramers of Ω-crystallin assembled and interacted with Xa. We found that the multimerization of Ω-crystallin led to the formation of good repositories for the pigment molecules, where rigid docking of Xa to the tetramer showed numerous distinct binding pockets in the central cavity (Fig. 6c and Supplementary Fig. S6). The top five poses had a high average binding energy of −10.7 ± 0.2 kcal mol^−1^ (error is reported as standard deviation). Furthermore, we predicted that multiple pigment molecules could be stabilized and stored within the tetramer structure without inducing significant conformational or structural changes in the binding pocket. Additionally, we calculated the molecular electrostatic potential map of the tetrameric Ω-crystallin and observed a separation of electrostatic potential within each tetramer, where regions of higher electrostatic potential (blue) and regions of lower electrostatic potential (red) were located in different areas of the protein (Fig. 6c). Given the distribution of the potential map, it was possible to conceive of a mechanistic pathway where individual tetramers could coacervate to form a protein-pigment superstructure, in a process similar to a polymerization mechanism previously proposed for S-crystallin in the octopus lens^39^. While polymerization of crystallins in the lens is generally viewed as a negative phenomenon, leading to opacity and cataracts, polymerization of Ω-crystallin into nanostructured granules in chromatophores could be a positive feature during signaling and camouflage, permitting enhanced light-scattering for color amplification during actuation.

**Fig. 7:**
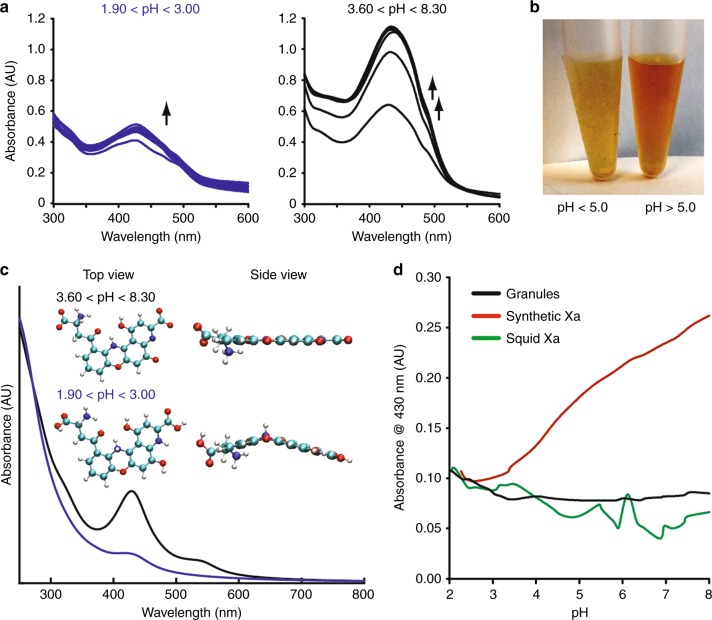
pH-dependent color change of compound Xa. **a** Variations in absorptive behaviors of Xa associated with an increasing pH. **b** A bright-field image of the two visible colors displayed by compound Xa at high (>5.00) and low (<5.00) pH values. **c** Proposed molecular structures of Xa and their corresponding absorption spectra calculated under neutral (designated with a neutral charge, black line) and acidic (designated with *a* + 3 charge, blue line) conditions. **d** The absorbance intensity (collected at 430 nm) associated with varying the pH in the synthetic Xa compared to granules and the pigment-extracted from the granules (squid Xa)

Considering the high affinity between Ω-crystallin and Xa, especially within the largely solvent-excluded cavity in the tetrameric Ω-crystallin structure presented in Fig. 6c, we reasoned that the sequestration of Xa within tetrameric Ω-crystallin offers an environment that may potentially prevent structural or optical changes of the pigment. To test this hypothesis, we probed Xa’s absorptive properties as a function of pH both as a free pigment and as part of the granule, where we selected pH as a parameter due to the multiple ionization and protonation sites on Xa. To decouple the contributions of any proteins in the system, we first synthesized Xa via the oxidative cyclization of 3-hydroxy-kynurenine^55^, then monitored its color as a function of pH. When the pH was varied from 1.90 to 8.90, we observed two distinct patterns associated with the absorption profile (Fig. 7a). At pH < 3.00, the pigment was pale-yellow with a *λ*_max_ ~ 430 nm. Then at pH ~3.60, the solution transitioned to a darker color that ultimately saturated at pH 7.70 (Fig. 7b). The observed intensity in color that was reversible over 12 total acquisition cycles, suggesting a dependency of the pigment’s color intensity to its protonation state.

To better understand the effects of pH on the pigment structure and optical activity, we created and tested a variety of computational molecular structures in different protonation states using DFT calculations. We found two molecular structures that reproduced the experimental absorption spectra—one under charge neutral (8.40–5.50) and one +3 charged molecule under acidic (<3.00) conditions (Fig. 7c). Under neutral conditions, a theoretical *λ*_max_ = 429 nm was observed, which was almost identical to our experimental value (*λ*_max_ = 430 nm). For Xa under acidic conditions, we observed a diminished peak at 430 nm, similar to our experimental absorption spectrum (Fig. 7a–c), suggesting an acid catalyzed prototropic tautomerization at the cyclohexadienone carbonyl under acidic conditions that caused the break in aromaticity and loss of color in the polycyclic system (illustrated in Fig. 7c, inset). These data suggested that Xa alone was susceptible to both structural and functional changes dependent on its protonation state. Given these variations, we hypothesized that the stability offered through the hydrophobic and polar interactions predicted in Fig. 6a, b was necessary to stabilize the heterocycle and mitigate these changes in color. To test this, we varied the pH of the isolated and purified chromatophores granules and the pigment-extracted from the granules—both abundant in Ω-crystallin—and compared their behavior to the synthetic Xa (Fig. 7d). As predicted, we observed negligible changes to color intensity as a function of pH in the granules and extracted pigment when compared to the synthetic Xa (with no proteins). In the future, we will expand these observations to evaluate whether the presence of Ω-crystallin increases the longevity of Xa, effectively protecting it from photodegradation within the chromatophore.

## Discussion

The optical properties of cephalopod skin continue to surprise. Chromatophore organs in squid skin, thought to be exclusively absorptive and pigmentary, are also structurally reflective at select viewing angles. This previously unexplored feature is accompanied by the discovery of reflectin surrounding the chromatocyte and crystallin residing within the chromatocyte. Reflectin has long been recognized as an essential component that generates static or neurally tunable iridescence (iridocytes) as well as exceptionally bright broadband white (leucocytes) in skin layers below the chromatophores^9,10^. On the other hand, the S-isoform of crystallin in squid eyes is present as a colloidal gel with a refractive index gradient that enables the squid lens to counteract spherical aberration^22^, while the Ω-isoform of crystallin is present in the photophore of the bobtail *E. scolopes*, believed to relieve oxidative stress in its light organ^25^. Using an annotated proteome specific to squid chromatophores, we confirm the presence of these proteins in the dermal chromatophores, spatially segregated within the pigment sac (Ω-crystallin) and surrounding it (reflectin).

It is unusual for the elements of pigmentary and structural coloration to interact as intimately as seen in the squid chromatophore. In select species of butterflies, spiders, reptiles, and birds^42,56^, these elements can be observed within the same tissue but they are generally located in discrete cell layers or assemblages (or, if blended within one tissue as with butterfly scales^42^, they produce a single visual effect). For instance, various skin colors are produced in day geckos via iridophore cells interspersed with melanophores and erythrocytes; however, this combination generates static color patterns only^57^. In contrast, the dynamic coloration of the panther chameleon is made possible by two distinct layers of cells, one that features iridophores that modulate brightness and a second layer that uses either iridophores or pigmented cells to control hue^58^. The spatial segregation of color sources into cell layers described in these systems is not dissimilar from squid skin viewed at the large scale, where there is a topmost layer of chromatophores and a subjacent layer of iridophores. However, it is now clear that the squid chromatophore organ, unlike most of these other systems, has closely intertwined pigmentary and structural coloration components at the molecular and cellular level to produce dynamic colors that can appear both richly pigmentary or brightly iridescent.

The abundance of Ω-crystallin (a structural homolog of aldehyde dehydrogenase) and its affinity to xanthommatin suggest dual roles for this protein that contribute to (i) the nanostructure of the granule and (ii) the stabilization and/or sequestration of xanthommatin in the granule, which would otherwise be a toxic byproduct of tryptophan metabolism. Both features are likely contributors to pigment color retention, light absorption, and scattering, as the chromatophore experiences a change in presented surface area during actuation. From the computational analysis, we posit a mechanism describing the coacervation of the protein-pigment complex into granules—a process that has eluded researchers for decades. Based on the distinct electrostatic gradients on the protein surface, we reason that the positively charged regions of one tetramer may stabilize and bind to the negatively charged regions of an adjacent protein (monomer or tetramer) leading to structural associations that may propagate to form the nanostructured granules in the chromatophore. While further investigations are required to support this position, our findings reveal a unique combination of Ω-crystallin and pigments in the granules that may play an important role in regulating color within the chromatocyte.

Outside the chromatocyte, we describe a mode of reflection via structural color originating in part from the chromatophore sheath cells, which are shown here to contain reflectin. Since the chromatocyte and sheath cell membranes are deformable yet closely apposed, chromatophore actuation that expands the pigment cell into a flattened disc might automatically produce a parallel membrane geometry conducive to wavelength-selective interference. While this hypothesis is speculative, it is supported by our direct optical observation of chromatophore iridescence in the expanded state with certain narrow angles of lighting, and by the observation of reflectin aggregations intimately associated within sheath cells tightly surrounding the chromatocyte. It is particularly noteworthy that no platelets (as in iridocytes) or spheres (as in leucosomes) have been found within the sheath cells or elsewhere in or near the chromatophore organ in our numerous micrographs. Instead, at high optical magnification the reflectin antigenicity in fixed tissue is observed as small granular vesicles dispersed in seemingly disordered fashion amidst the convoluted sheath cells, which themselves must morph radically during the 15x areal expansion of the pigmented chromatocyte when it is maximally distended by the radial muscles for full color dispersal (Fig. 2c, e). Considering that reflectins were recently reported to contain no transmembrane domains^59^, we speculate that one or more reflectin isoforms could be condensed (dynamically) along or within the numerous folded sheath cell membrane invaginations that appose each other with sub-micron gaps outside the chromatocyte (Supplementary Fig. 3A). In principle, such folds might constitute a geometrical compartmentalization for reflectin that when flattened could yield structural color using incident photons and those optically filtered by the absorptive granules in the pigment saccule and reflecting upwards. This is consistent with a previous study in which self-assembled films of reflectin exhibited structural color due to thin-film interference, which is highly dependent on film thickness^29^.

**Fig. 8:**
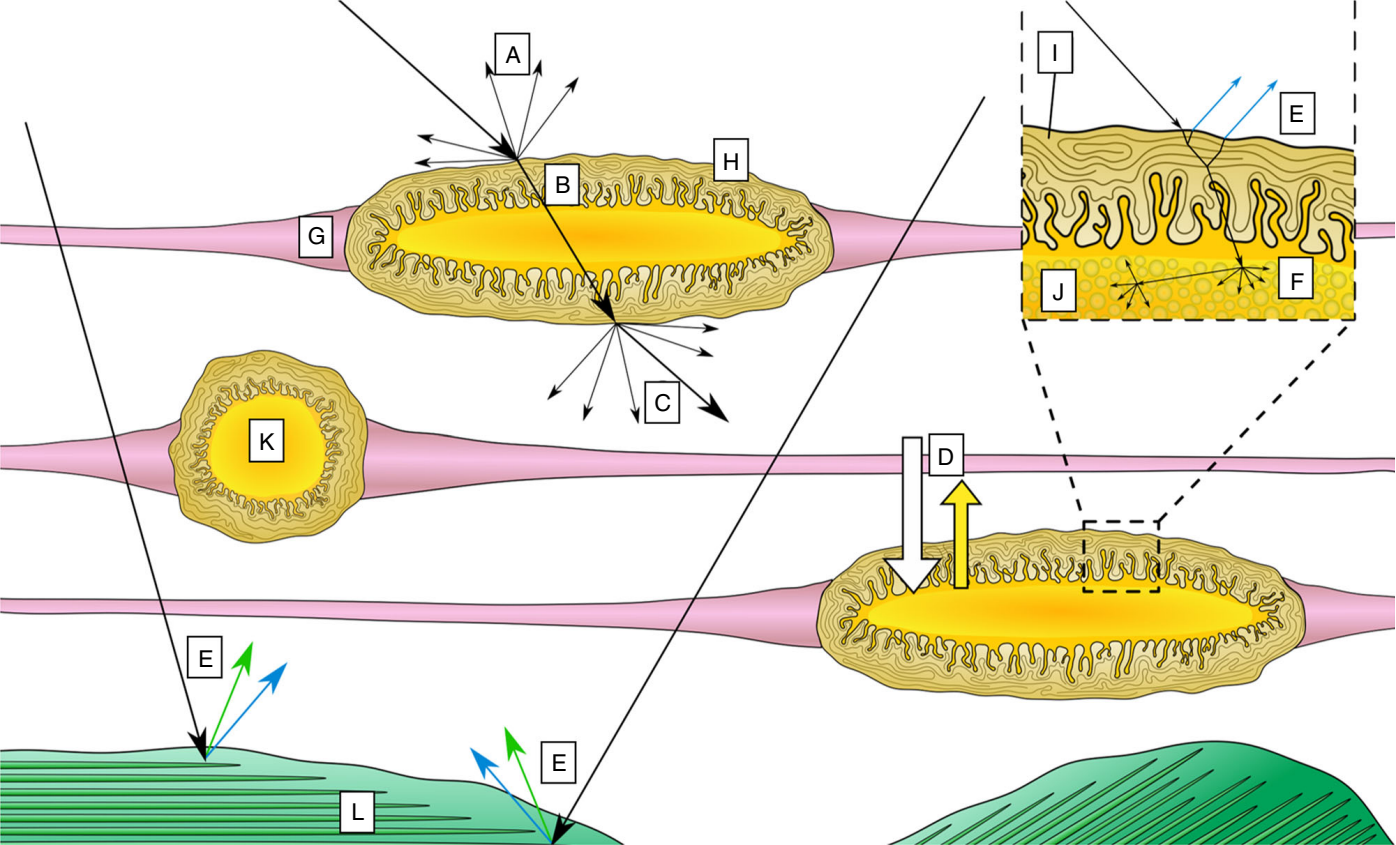
Predicted optical effects within and around the chromatophore organs. The associated optical phenomena proposed based on our current and previous (see ref. ^17^) findings are represented as **a** back scatter, **b** refraction, **c** forward scatter, **d** absorption (of non-yellow wavelengths), **e** multilayer interference, and **f** diffuse scattering, where **g** represents the radial muscle fibers; **h** represents the sheath cells; **i** represents the cytoplasm of the sheath cells; **j** represents individual granules; **k** represents the a collection of granules within the yellow chromatocyte; and **l** represents an iridophore, deeper in the dermis. For simplicity, only yellow colored chromatophores are illustrated here

How might reflectin influence light from the chromatophore? Since the sheath cells completely surround the pigment granules in the chromatocyte, light that enters the expanded chromatophore from the surface will pass through the sheath cells (and hence encounter the reflectins) to interact with pigment granules before they exit vertically to produce reflection via scattering, effectively doubling its effect. In other cases, light could enter chromatophores from below (the skin is often transparent) to also pass through sheath cells on both sides of the pigment sac. In such cases this structural coloration could, for example, amplify the brightness of the pigments, as we have seen in biomimetic analogs of chromatophores^17^ and have illustrated in Fig. 8.

While our understanding of the reflectin family is still growing (by the last count there are dozens of isoforms), we observed only five in our MS/MS analysis (A1, A2, B1, C1, and 3) and two (A1 and A2) from our immunocytochemical labeling. As the antibodies used in our study were highly unlikely to have revealed all reflectins, a near-future task will require in situ labeling of chromatophores using RNA-based probes to better understand the spatial distribution of these proteins.

When taken together, the compositional, computational, and optical analyses of the squid chromatophore presented here—from molecules to the whole organ—provide insights into the ultrastructure and chemical composition of these organs. While cephalopod chromatophores have been thought to be solely pigmentary organs for decades^5,11,12,60^, our findings demonstrate an additional photonic mechanism via structural iridescence that could conceivably be used to enhance the dynamic range of color presented by the squid, or to provide an additional specular visual cue contextually detectable by conspecifics, predators, or prey. Altogether, these findings along with the knowledge that approximately 15 additional isoforms of reflectins exist in cephalopods with functions yet to be explored, suggest there is still much more to be learned about these complex systems, including a better understanding of molecular mechanisms regulating these dynamic biophotonic processes especially as it pertains to the future designs of engineered materials with dynamic optical capabilities.

## Methods

### Isolation of chromatophores for whole-cell analysis

Adult squid, *D. pealeii*, were collected by the Marine Resources Center (MBL, Woods Hole, USA). As ethics approval for cephalopods is not required in the United States, the Institutional Animal Care and Use Committee has no authority for review of such protocols. However, the authors conducted all experiments with sincere efforts towards ethical care and treatment of these animals, where the number of individuals was minimized and an aquatic veterinarian at the Marine Resources Center of MBL was routinely consulted throughout the season. For our experiments, squid were anesthetized in ethanol and sacrificed. Individual chromatophores were isolated by removing sections of dermis from *D. pealeii*. Prior to removal of dermis, the upper hyaline and epidermal layer was removed. The chromatophore containing skin layer was then removed from the animal, with careful attention to exclude the iridophore layer below. The tissue sections were immersed overnight in a solution of collagenase (~6 mg mL^−1^ *Clostridium histolyticum*) in sea water. Samples were triturated to encourage the breaking up of tissue. After a final trituration, the samples were washed five times with filtered sea water or Tris buffer. Finally, individual chromatophores were plucked out of the solution manually via micropipette, aided by stereomicroscope, and they were separated by color. Brown and yellow chromatophores were collected from dorsal mantle, red chromatophores were obtained from the ventral dermis.

### Collection of chromatophore granules

Chromatophore granules were collected in a color independent manner. *D. pealeii* were dissected and skin samples were collected as before. The skin sections were aliquoted into miniature centrifuge tubes with 0.5 mL of collagenase and papain solution. Samples were vortexed, sonicated, and centrifuged. The supernatant was removed, and more enzyme solution was added. This was repeated three times total. After the final centrifugation, supernatant was removed and a homogenization solution was added. After vortexing and sonicating the mixture, any large tissue pieces were removed, and the remaining suspension was centrifuged. This step was repeated three times to ensure that the enzyme solution was completely inhibited and any unreacted tissue was removed, leaving behind the water-insoluble granules^14,15^.

### Pigment extraction

The granules were rinsed with water and centrifuged. The supernatant was removed and replaced with a solution of 0.5% hydrochloric acid in methanol. This suspension was vortexed and sonicated, but the supernatant was collected as the pigment, while the pellet, consisting of de-pigmented granules, was saved separately similar to previous protocols^15^.

### Protein purification and analysis using mass spectrometry

The yellow, red or brown chromatophore cells were lysed by sample buffer and sonicated to solubilize proteins. Proteins were separated by sodium dodecyl sulfate-polyacrylamide gel electrophoresis (SDS-PAGE) and stained with Coomassie blue G250.

Each lane of different color chromatophore cells were divided into five bands, reduced and alkylated, digested in-gel with trypsin. Tryptic peptides were extracted with 50% acetonitrile in 2% formic acid. Volume of the digestion mixture was reduced to 3.5 µL, and 1 µL was injected in a Dinonex Ultimate 3000 RSLC nano UHPLC system (Dionex Corporation, Sunnyvale, CA), and separated by a 75 µm x 25 cm PepMap RSLC column (100 Å, 2 µm) at a flow rate of ~450 nL min^−1^. The eluant was connected directly to a nanoelectrospray ionization source of an LTQ Orbitrap XL mass spectrometer (Thermo Scientific, Waltham, MA). LC-MS data were acquired in an information-dependent acquisition mode, cycling between a MS scan (*m*/*z* = 315–2000) acquired in the Orbitrap, followed by low-energy CID analysis on three most intense multiply charged precursors acquired in the linear ion trap.

For the granules, pigment, and extracted granules, the samples were denatured and separated by SDS-PAGE. After staining the gel with Coomassie blue G250, visible bands from all three samples were excised for in-gel digestion and MS/MS analysis as above.

### Sample collection and RNA sequencing of squid chromatophore

Existing RNA sequencing data were accessed from the Short Read Archive (SRA) under accession number SRX817967. Briefly, specimens of *D. pealeii* were collected by the MBL. Fresh tissue was dissected from the chromatophore layer of the skin. RNA was extracted with the RNAqueous kit (Life Technologies, Carlsbad, CA) and stored at −80 °C. RNA libraries were prepared using TruSeq Stranded mRNA Prep Kit (Illumina, San Diego, CA) and sequenced using an Illumina HiSeq 2000 instrument^61^.

### Transcriptome assembly

The RNA sequence data were assembled using the Oyster River Protocol^62^. In brief, this assembly procedure began by implementing a read-error correction algorithm^63^. Next, Illumina sequencing adapters were removed, as were nucleotides whose PHRED score was <5^64^. The corrected and trimmed reads were assembled, after which the assembly was evaluated using the software packages BUSCO^65^ and TransRate^66^. The transcriptome was annotated using the software package dammit, which included the production of amino acid sequences via open reading frame identification and subsequent translation. The resultant transcriptome was then used as described below.

### Protein identification using BLAST

The centroided peak lists of the CID spectra were generated by PAVA^67^ and searched against a protein database translated from the transcriptome of squid *D. pealeii* chromatophores. A random-concatenated version of this protein database was also generated to assess false discovery rate of protein identification.^68^ In addition, the Swiss-Prot protein database was also included to accommodate common contaminations in sample handling. Batch-Tag, a program in the in-house version of the University of California San Francisco Protein Prospector version 5.20.23, was used to identify proteins. A precursor mass tolerance of 15 ppm and a fragment mass tolerance of 0.5 Da were used for protein database search. Protein hits were reported with a Protein Prospector protein score ≥ 22, protein discriminant score ≥ 0.0 and a peptide expectation value ≤ 0.01^69^. This set of protein identification parameters threshold did not return any substantial false positive protein hit from the randomized half of the concatenated database. For each of the identified protein entries, the amino acid sequence was searched against Swissprot database (downloaded January, 2017) using the BLASTP (Basic Local Alignment Search Tool) program for function annotation. The BLASTP search was conducted using default parameters, with the exception of the e- value, which was set to 1*e*^−10^.

### Data processing

The spectra count for each of the identified protein entries was recorded. For the whole, red, brown, and yellow chromatophore cells, normalization was based on the assumption that the amount of all proteins constituting chromatophore cells of different colors was similar, though the amount of individual proteins might vary. Therefore, the sum of spectra counts for identified protein entries in each color of chromatophore cells was used to calculate the normalization factor for protein loading, 1, 2.2, and 1.8 for yellow, red, and brown respectively. Spectra counts were divided by normalization factors before direct comparison of identified protein entries in yellow, red and brown chromatophore cell samples.

In order to compare relative abundance of different proteins within samples, all spectra counts for each protein were normalized by the number of peptide fragments produced upon trypsin digestion. This was done by dividing each protein by the number of peptides it is expected to produce, then multiplying it by 65.5, the average number of expected peptides across the protein dataset used. These numbers were calculated using the Protein Prospector MS-Digest tool at http://prospector.ucsf.edu and further supported by statistical analyses as described in Supplementary Notes 1.

### Immunocytochemistry

Adult *D. pealeii* (approximately 6 inches long from mantle to tail) were obtained live in the late Summer and Fall season from the Marine Resources Center at the MBL. They were anesthetized in 2–3% ethanol for 10 min, then decapitated. The mantle or fin skin biopsies were removed from the underlying body musculature and pinned out under sea water in a petri dish half-filled with Sylgard to prepare for immunofluorescent imaging (details in Supplementary Notes 2).

### Synthesis of Xa

Xa was synthesized via the oxidative cyclization of 3-OHK using potassium hexacyanoferrate in a phosphate buffered saline solution (PBS, pH = 7.4) according to a previous report^55^. An orange product was purified using an OASIS WAX solid phase extraction with water and collected using 0.5% (v/v) hydrochloric acid in methanol (HCl-MeOH). The purified product was then characterized using liquid chromatography-tandem mass spectrometry, verifying the purified product was attained.

### Spectrometric measurements

A 1 mM solution of synthetic Xa, squid extracted and purified Xa, and a 10% (v/v) solution of granules were prepared in water and titrated to a starting pH of ~2. The solution was then titrated with 166 mM NaOH until the pH reached ~10. During each point in the titration, the pH and absorbance of the solution was determined. pH levels were measured using a Fisher Scientific Accumet AP110 pH meter (Fisher Scientific, Waltham, MA). Absorbance was measured using a Spectramax® M5 microplate reader. All samples were measured in triplicate from at least two technical replicates. Data shown are representative of each condition. All data is normalized to starting intensity values across the runs.

### Computational methods

The structure for the pigment molecule (Xa) was obtained after geometry optimizations from different initial structures with DFT in implicit solvent (Supplementary Notes S3). Homology models of Ω-crystallin and reflectin were constructed from the identified protein sequences and refined with replica exchange molecular dynamics (MD) (Supplementary Notes S3). The tetrameric structure of Ω-crystallin was constructed through structural alignment of four Ω-crystallin monomers (Supplementary Notes S3) and the electrostatic potential map of the crystallin monomer and tetramer were also calculated (Supplementary Notes S4). Xa was then docked to the Ω-crystallin and reflectin structures where the binding energies were obtained (Supplementary Notes S5), and the interacting ligands and amino acid sidechains were mapped and visualized (Supplementary Notes S6). To obtain the Xa absorption spectra, the first 200 excited states of the Xa molecules in protonation states were calculated and a Lorentzian broadening with full width at half maximum (FWHM) of 30 nm was applied to the raw results (Supplementary Notes S7).

### Reporting summary

Further information on experimental design is available in the Nature Research Reporting Summary linked to this article.

## Supplementary information

Supplementary Information

Description of Additional Supplementary Files

Supplementary Movie 1

Supplementary Movie 2

Supplementary Movie 3

Supplementary Movie 4

Supplementary Movie 5

Reporting Summary

Source Data

## Data Availability

Data supporting the findings of this manuscript are available from the corresponding authors upon reasonable request. A reporting summary for this Article is available as a Supplementary Information file. The mass spectrometry proteomics data that support this study have been deposited to the ProteomeXchange Consortium via the PRIDE^70^ partner repository with the dataset identifier PXD011975. The source data underlying Fig. 2b, d, Figs. 3b, 7a, d, and Supplementary Figs S1 and S3c are provided as a Source Data file.
