# Supplementary material for: Dynamic pigmentary and structural coloration within cephalopod chromatophore organs
Source: Nat Commun. 2019 Mar 1;10:1004. doi: 10.1038/s41467-019-08891-x (PMC6397165; doi:10.1038/s41467-019-08891-x)
Supplement: Supplementary file 1 — Supplementary Information [file 41467_2019_8891_MOESM1_ESM.docx]

**Dynamic pigmentary and structural coloration within cephalopod chromatophore organs**

Thomas L. Williams^1*^, Stephen L. Senft^2*^, Jingjie Yeo^3,4,5*^, Francisco J. Martín-Martínez^4^, Alan M. Kuzirian^2^, Camille A. Martin^1^, Christopher W. DiBona^1^, Chun-Teh Chen^4^, Sean R. Dinneen,^1^ Hieu T. Nguyen,^6^ Conor M. Gomes^1^, Joshua J. C. Rosenthal^2^, Matthew D. MacManes^6^, Feixia Chu^6^, Markus J. Buehler^4^, Roger T. Hanlon^2#^, Leila F. Deravi^1#^

^1^ Department of Chemistry and Chemical Biology, Northeastern University, Boston, MA 02115, United States.

^2^ The Marine Biological Laboratory, Woods Hole, MA 02543, United States.

^3^ Department of Biomedical Engineering, Tufts University, Medford, MA 02155, United States.

^4^ Department of Civil and Environmental Engineering, Massachusetts Institute of Technology, Cambridge, MA 02139, United States.

^5^ Institute of High Performance Computing, A*STAR, Singapore 138632, Singapore.

^6^ Department of Molecular, Cellular, and Biomedical Sciences, University of New Hampshire, Durham, NH 03824, United States.

These authors contributed equally: Thomas L. Williams, Stephen L. Senft, Jingjie Yeo

Correspondence and requests for materials should be addressed to L.F.D. (email: l.deravi@northeastern.edu) or to R.T.H. (email: rhanlon@mbl.edu)


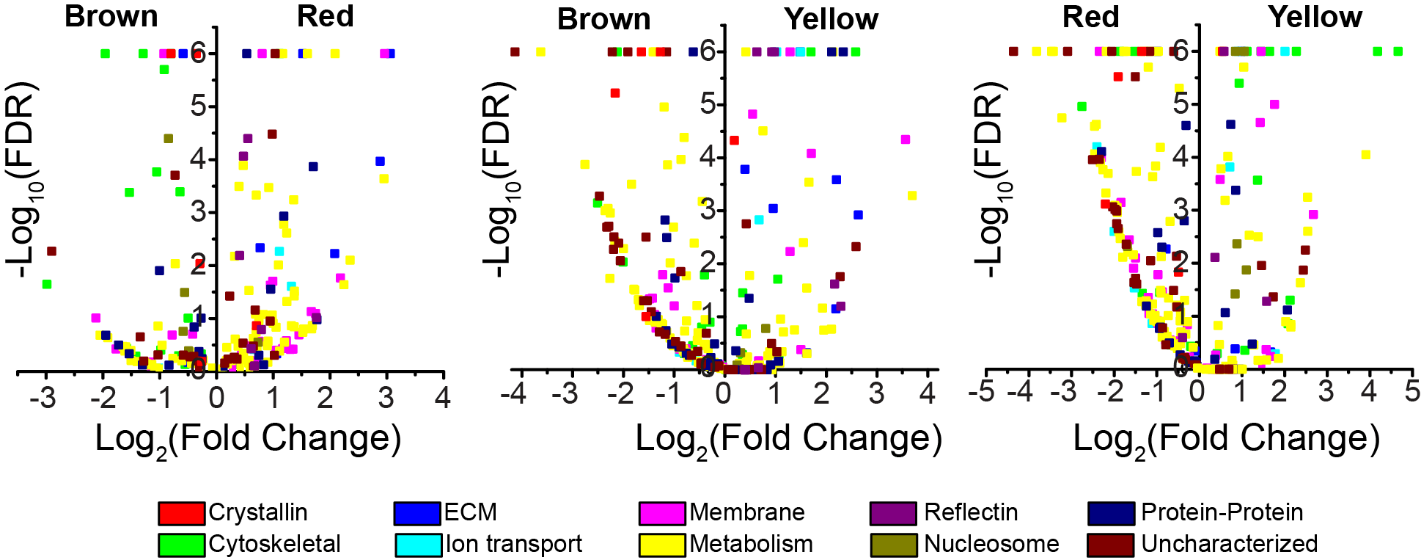


**Figure S1.** QSpec analysis showing both scale and significance of differences in protein expression, as measured by spectral count MS/MS data. The three chromatophores are compared pairwise (Brown vs. Red, Brown vs. Yellow, and Red vs. Yellow) to show how protein abundance differs between brown, red, and yellow chromatophores. For each protein detected in more than one chromatophore, significance is expressed as the negative log of the false discovery rate (-log_10_(FDR)), where a value greater than 1.301 signifies an FDR of less than 5%, while the magnitude of the differential expression between the two types of chromatophore is shown as the log base two of the fold change (log_2_((Fold Change)), where a value of 1 or -1 means that the protein is twice as abundant in the indicated chromatophore color as compared to the other. For proteins with a reported FDR of 0, the -log(FDR) was set to 6. Each protein was also categorized by function based on BLAST identification, as indicated by color.

**
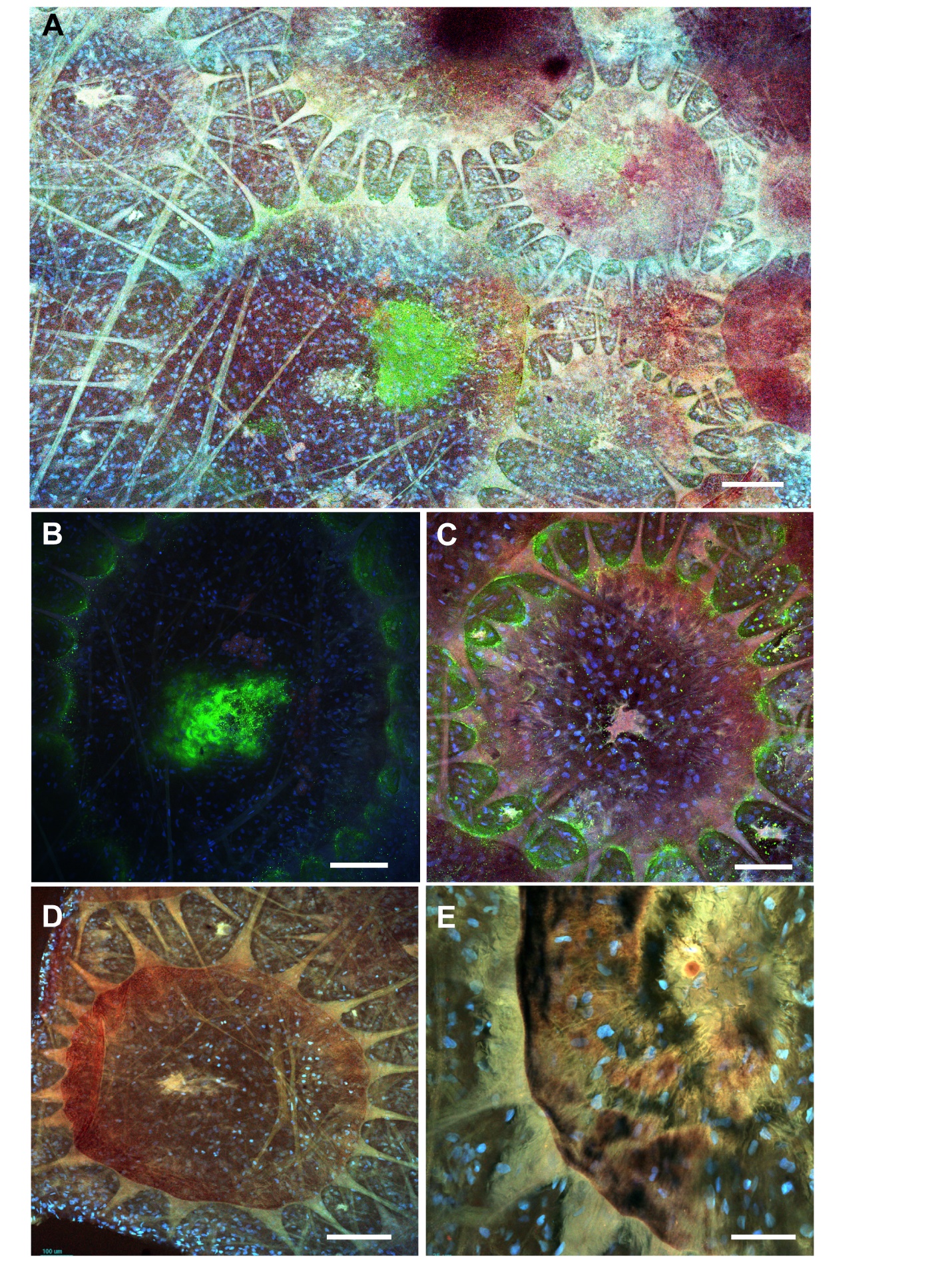
**

**Figure S2**. (A) Low magnification tiled confocal image (10x objective) of adult squid skin from anterior mantle. Tissue processed for ICC against reflectin A1/A2 [Morse, ~1:250] (green), counterstained with DAPI to show nuclei (blue); other structural details, such as the radial muscles and pigment-filled saccules, are visible by autofluorescence. A large (membranous) reflectin-positive mass is located near the center of the chromatophore. This was seen frequently, in addition to the scalloped pattern of label around the proximal portion of the radial muscles and near the perimeter of the chromatophores. Color values enhanced by histogram-equalization to accentuate background anatomy. (B) Confocal image of a single (brown-type) chromatophore, stained for reflectin (green) by ICC, nuclei counterstained using DAPI. Heavy staining in a region central to the chromatophore (located on its surface, not within the saccule). (C) Confocal image of a single (red-type) chromatophore, stained for reflectin (green) by ICC, nuclei counterstained using DAPI. Red and blue channels were enhanced to accentuate background anatomy. A large mass of membrane (pink) is visible on the surface of the chromatophore, here only moderately stained for reflectin (small green dots). Many of the nuclei (blue) over the chromatophore are likely from sheath cells (others may be from fibroblasts). The membranous striations visible over the chromatophore are interpreted as primarily sheath cell membrane material. (D, E) Control ICC images. (D) No primary antibody; 488 secondary present, DAPI counterstain (confocal, 10x objective). (E) No secondary; primary reflectin antibody [Goodson. ~1:200] present, DAPI counterstain (confocal, 20x objective). Scale bars for A-C are100 µm; D is 200 µm; E is 50 µm.

**
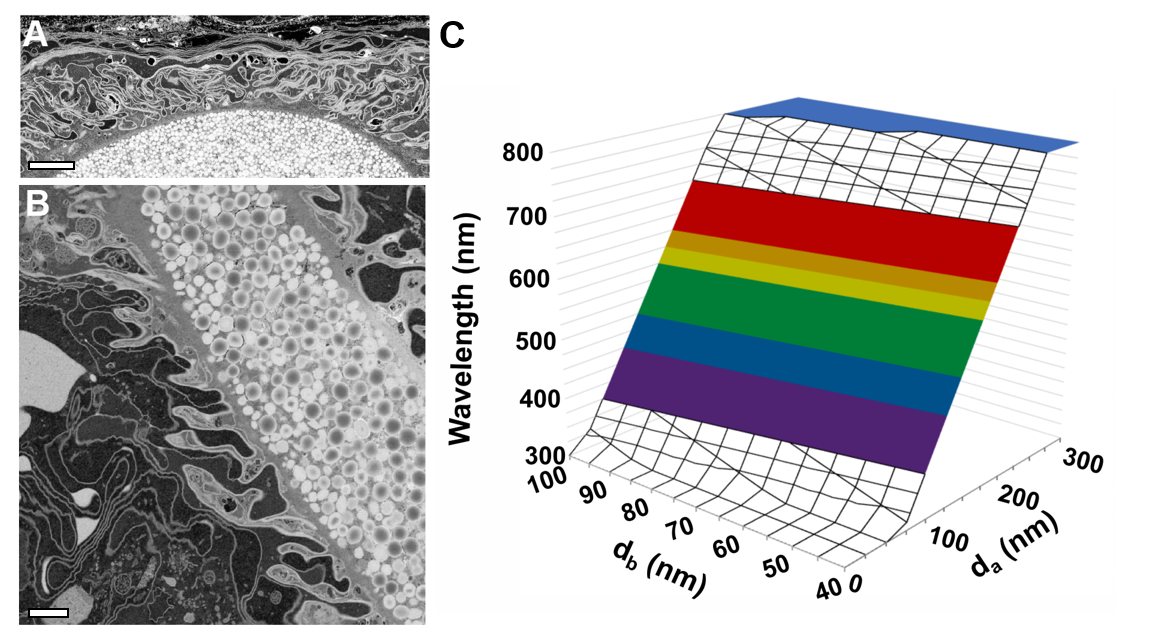
**

**Figure S3.** Chromatocyte processes interdigitate extensively. (A) An electron micrograph of a compacted adult chromatophore (from serial block face imaging (SFBI) stack, imaging of our material courtesy of the Field Electron and Ion (FEI) Company). The curved sacculus is filled with pigmented granules (reddish-brown type). Extensions of sheath cytoplasm (darker gray) project nearly to the saccule wall faintly visible within the chromatocyte. At the top are a series of parallel membranes, likely all from sheath cells. The section orientation with respect to the skin surface is uncertain but is most likely perpendicular to it. Scale bar is 5 µm. (B) An oblique electron micrograph from SBFI stack of partially expanded adult chromatophore (imaging of our material courtesy GATAN). Saccule filled with pigmented granules (brown type) running diagonally at upper right. Portions of a radial muscle and its mitochondria are visible at extreme upper left. Numerous chromatocyte fingers (light grey) periodically extend outward from near the saccule into a region filled with intricately folded sheath cell processes (dark greys). Scale bar is 2 µm. (C) Using the layer height ranges (± 2 standard deviations on the average) from A and B, peak reflected wavelength, λ, was estimated using:

$m\lambda=2\left( n_{a}d_{a}\cos\theta_{a}+n_{b}d_{b}\cos\theta_{b} \right)$(Eq. 1)^1^

where *m* is an integer or half integer, *n*_a_ and *n*_b_ are the refractive indices of the sheath cell cytoplasm and membrane, respectively (*n*_a_ > *n*_b_), and *ϴ*_a_ and *ϴ*_b_ are the angles of refracted light in their respective layers. Given that the sheath cell cytoplasm may have some dense regions of condensed reflectin, the refractive index of the sheath cell cytoplasm was approximated to be that of native reflectin protein,1.44^2^, and the membrane layer was assigned an approximate refractive index of 1.35^3^. The incident light angle was kept constant at 15˚. Based on these values, we estimated the ultra-structural related colors expected to be reflected by the chromatocyte. The *z* axis in (C) represents reflected wavelengths, and the *x* and *y* axes represent the distances between each sheath cell membrane (*d*_a_) and cytoplasm layer (*d*_b_), respectively. The visible region is shaded with its corresponding color. Given the limited resolution of the images collected using the dissecting scope, we cannot state which components of the chromatophore (e.g. the chromatocyte cytoplasm or surrounding sheath cells) are directly responsible for the observed interference. However, this analysis suggests that variations in microscopic spacing in the chromatocyte saccule could generate structural colors qualitatively similar to those observed macroscopically.

**
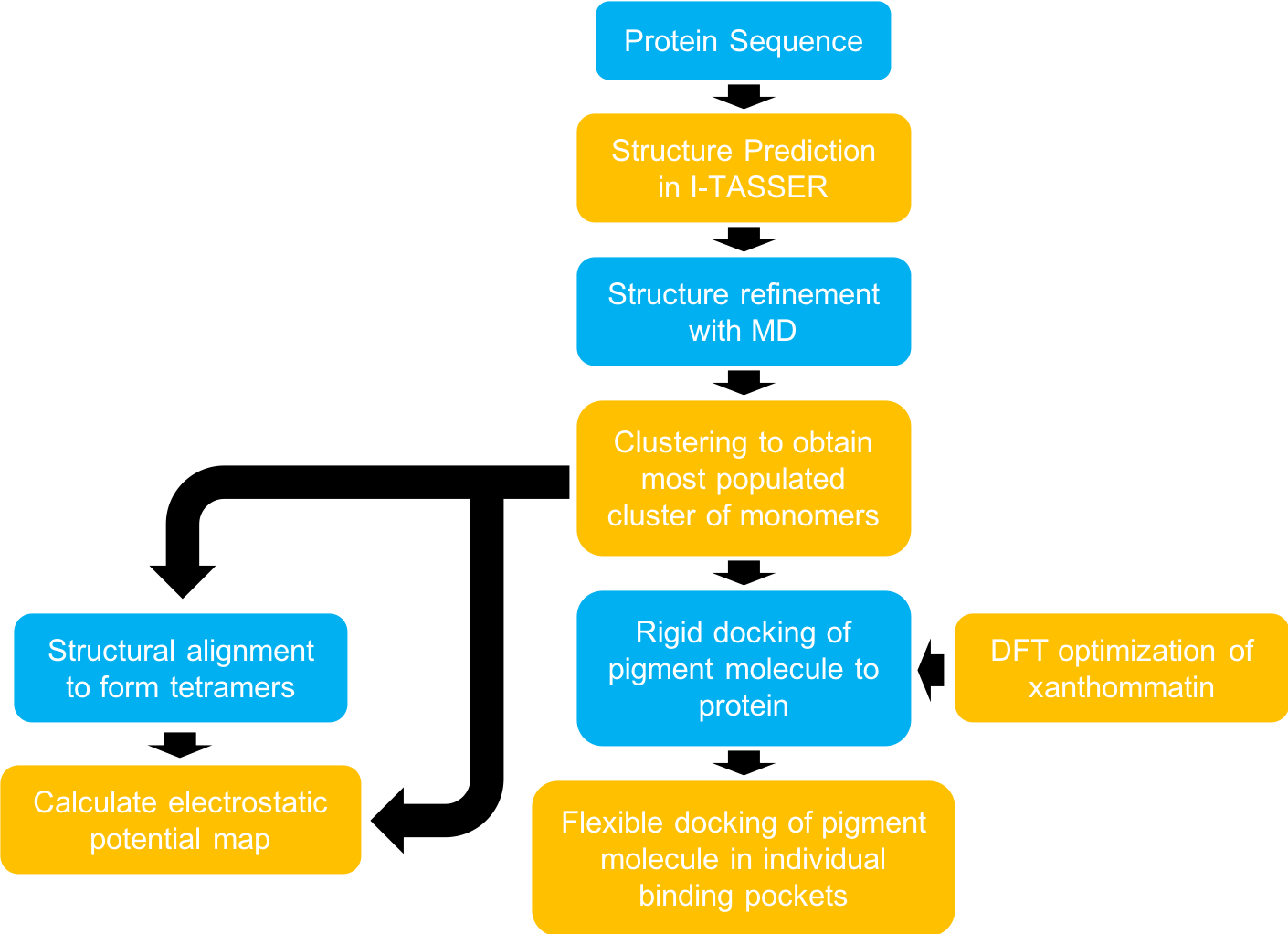
Figure S4.** Schematic of the entire flow of the computational simulations.


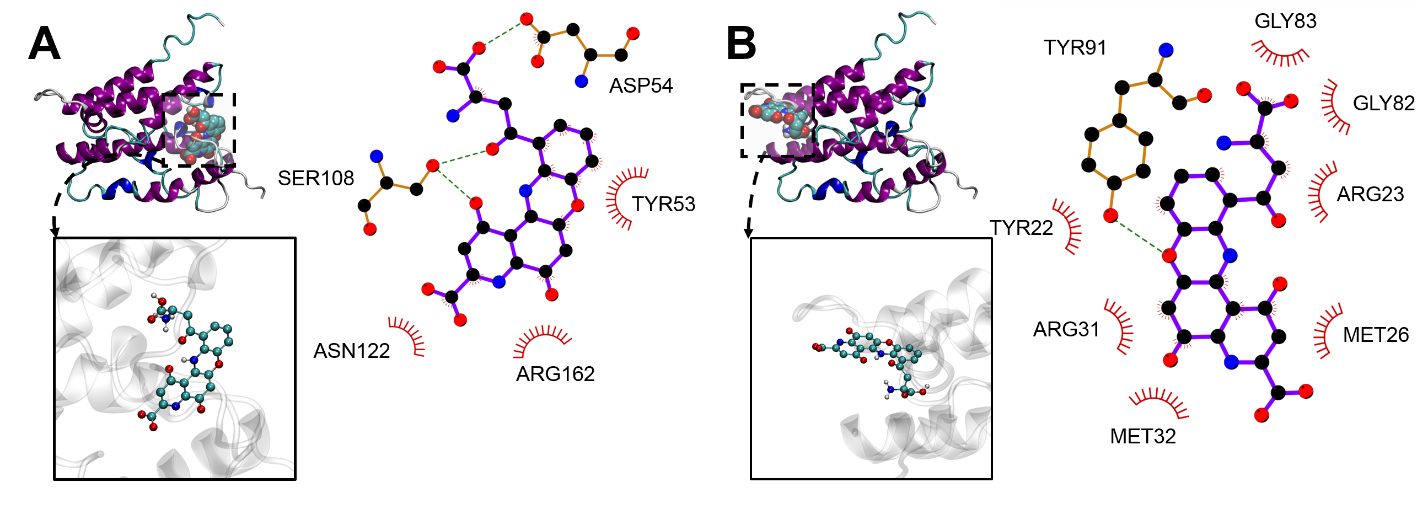


**Figure S5.** (A-B) Most energetically favorable binding poses from flexible molecular docking in two of the top-ranked binding pockets in reflectin. In each panel, the figures on the left show the orientation and size of the pigment molecule (space-filling beads) within the binding pockets of the respective proteins (cartoons colored by secondary structure). Inset provides closeup views of the molecule within the pockets. The figures on the right are schematics of the pigment molecule interacting with the amino acid sidechains in the respective pockets. Dotted lines indicate polar interactions between acceptor-donor pairs. Eyelashes (in pink) denote sidechains and atoms that are in contact with each other. Solid circles represent carbon (black), oxygen (red), nitrogen (blue), and sulfur (yellow).


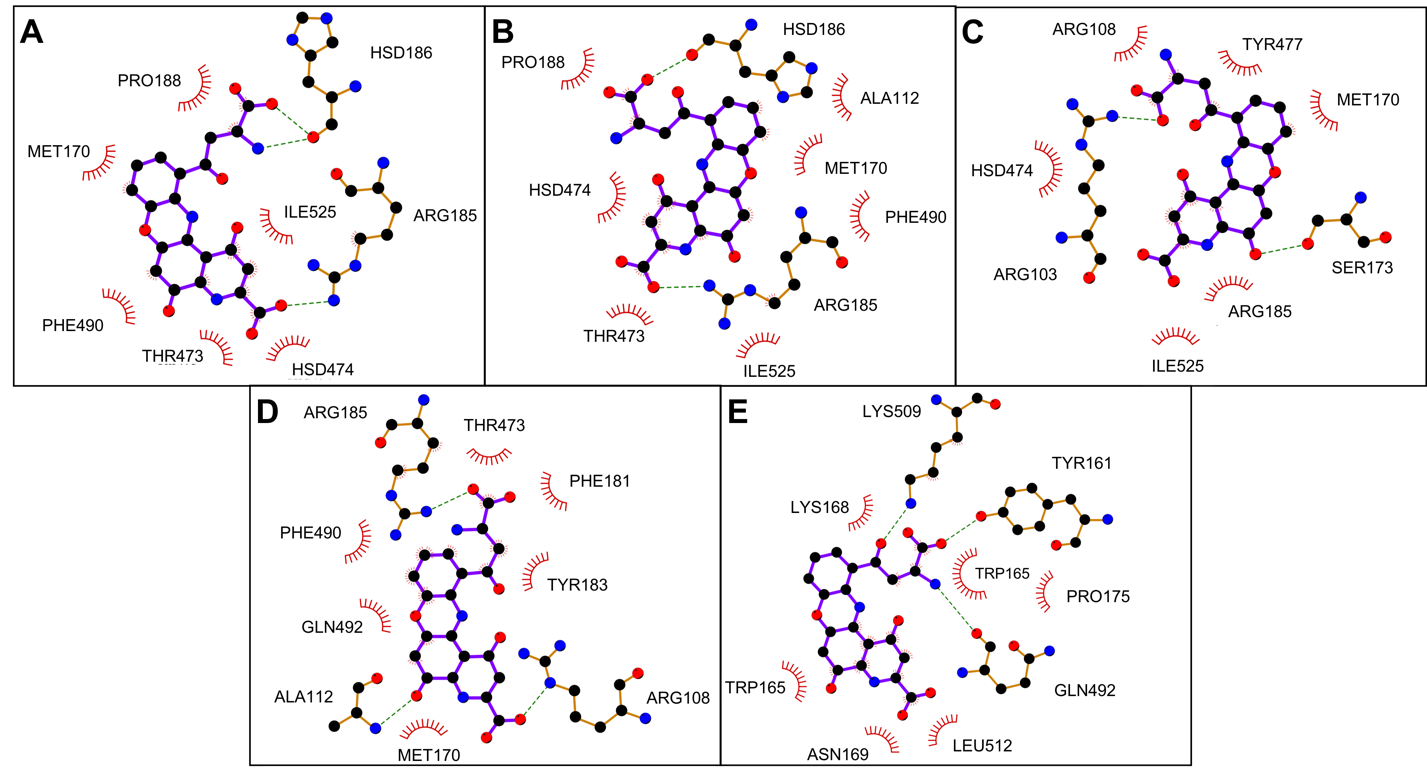


**Figure S6.** (A-E) The five top ranked poses of pigment in the tetramer binding pocket. Schematics of the pigment molecule interacting with the amino acid sidechains in the crystallin tetramer. Dotted lines indicate polar interactions between acceptor-donor pairs. Eyelashes denote hydrophobic interactions.

**Table S1.** Peptide count comparison of proteins identified from whole cell LC/MS/MS analysis. Peptide counts were normalized by predicted peptide count and by total peptide abundance per chromatophore type. (N =1 MS/MS for each color, which included a total of ~700 yellow, ~700 red, and ~1000 brown chromatophore organs that were collected and pooled from five total squid. Yellow chromatophores were pooled from two squid; Red chromatophores were pooled from two squid; and browns were pooled from three.)

| Accession # | Protein Name | Category | Brown | Red | Yellow |
| --- | --- | --- | --- | --- | --- |
| 11724 | Omega-crystallin | Crystallin | 256 | 170 | 290 |
| 6955 | Elongation factor 1-alpha | Metabolism | 100 | 87 | 141 |
| 12391 | PREDICTED: smoothelin-like protein 1 [Octopus bimaculoides] | Protein-protein | 52 | 83 | 60 |
| 11708 | PREDICTED: uncharacterized protein LOC106868335 [Octopus bimaculoides] | Uncharacterized | 18 | 46 | 25 |
| 21216 | Collagen alpha-4(VI) chain | Extracellular Matrix | 54 | 28 | 74 |
| 13220 | S-crystallin SL20-1-like [Octopus bimaculoides] | Crystallin | 144 | 101 | 26 |
| 10569 | Deoxyribonuclease gamma [Jaculus jaculus] | Metabolism | 66 | 63 | 39 |
| 8187 | Annexin A13-like isoform X2 [Octopus bimaculoides] | Protein-protein | 76 | 55 | 39 |
| 8052 | Lethal(2) giant larvae protein homolog 1-like [Octopus bimaculoides] | Cytoskeleton | 13 | 38 | 9 |
| 6946 | Glutathione S-tranferase mu | Metabolism | 69 | 64 | 69 |
| 5845 | Reflectin-like protein A2 [Doryteuthis pealeii] | Reflectin | 35 | 58 | 83 |
| 9491 | Myosin heavy chain isoform B [Doryteuthis pealeii] | Cytoskeleton | 2 | 0 | 20 |
| 3328 | Reflectin-like protein A1 [Doryteuthis pealeii] | Reflectin | 26 | 38 | 44 |
| 11887 | PREDICTED: uncharacterized protein LOC106876157 [Octopus bimaculoides] | Uncharacterized | 72 | 69 | 22 |
| 6590 | Reflectin-like protein B1 [Doryteuthis pealeii] | Reflectin | 31 | 44 | 76 |
| 6997 | Melanotransferrin 4 | Membrane | 19 | 7 | 31 |
| 8179 | Uniref: A0A0L8GHU8 | Metabolism | 38 | 50 | 9 |
| 9224 | PREDICTED: spectrin alpha chain-like isoform X3 [Octopus bimaculoides] | Cytoskeleton | 8 | 1 | 10 |
| 9342 | Hypothetical protein LOTGIDRAFT_217535 [Lottia gigantea] | Uncharacterized | 37 | 44 | 0 |
| 3200 | Mitochondrial carrier (TC 2.A.29) | Membrane | 20 | 40 | 64 |
| 3070 | Glyceraldehyde-3-phosphate dehydrogenase-like isoform X1 [Octopus bimaculoides] | Metabolism | 45 | 63 | 11 |
| 9086 | Spectrin beta chain | Cytoskeleton | 13 | 5 | 16 |
| 7174 | Sulfotransferase family cytosolic 1B member 1-like [Octopus bimaculoides] | Metabolism | 41 | 61 | 17 |
| 6812 | Immunoglobulin E-set | Metabolism | 4 | 20 | 34 |
| 20952 | ATP synthase subunit alpha | Metabolism | 15 | 12 | 23 |
| 356 | Histone H4 [Larimichthys crocea] | Nucleosome | 96 | 52 | 122 |
| 9087 | PREDICTED: spectrin beta chain isoform X5 [Crassostrea gigas] | Cytoskeleton | 7 | 2 | 9 |
| 3475 | Na+/K+ ATPase alpha subunit [Doryteuthis pealeii] | Ion Transport | 7 | 7 | 18 |
| 13200 | Annexin | Protein-protein | 21 | 15 | 31 |
| 16139 | PREDICTED: ATP synthase subunit beta, mitochondrial-like [Octopus bimaculoides] | Metabolism | 14 | 15 | 28 |
| 12158 | PREDICTED: smoothelin-like protein 1 [Octopus bimaculoides] | Protein-protein | 6 | 4 | 5 |
| 17802 | Tubulin beta-4B chain | Cytoskeleton | 32 | 16 | 23 |
| 13974 | Histone H2B | Nucleosome | 66 | 28 | 78 |
| 9250 | TUBA1B | Cytoskeleton | 31 | 12 | 29 |
| 2093 | Elongation factor 1-alpha | Metabolism | 17 | 15 | 23 |
| 7273 | Adenosylhomocysteinase [Astyanax mexicanus] | Metabolism | 25 | 29 | 0 |
| 9779 | Omega-crystallin [Enteroctopus dofleini] | Crystallin | 17 | 7 | 5 |
| 5905 | Voltage-dependent anion-selective channel protein | Ion Transport | 24 | 22 | 44 |
| 7408 | PREDICTED: 14-3-3 protein epsilon-like [Octopus bimaculoides] | Protein-protein | 28 | 20 | 28 |
| 2223 | PREDICTED: heat shock 70 kDa protein cognate 4 [Biomphalaria glabrata] | Metabolism | 16 | 11 | 8 |
| 13189 | Basement membrane-specific heparan sulfate proteoglycan core protein | Extracellular Matrix | 1 | 3 | 3 |
| 19991 | Tyrosinase-like [Octopus bimaculoides] | Metabolism | 31 | 33 | 13 |
| 8129 | PREDICTED: protein disulfide-isomerase 2-like [Octopus bimaculoides] | Metabolism | 12 | 6 | 15 |
| 10842 | Neutral and basic amino acid transport protein rBAT-like [Octopus bimaculoides] | Membrane | 12 | 7 | 10 |
| 3110 | Arginine kinase [Sepia pharaonis] | Metabolism | 22 | 20 | 7 |
| 3163 | Gelsolin-like protein 2 [Octopus bimaculoides] | Cytoskeleton | 19 | 11 | 17 |
| 9230 | Spectrin alpha chain-like isoform X4 [Octopus bimaculoides] | Cytoskeleton | 14 | 4 | 18 |
| 10990 | Non-muscle myosin II heavy chain [Doryteuthis pealeii] | Cytoskeleton | 4 | 3 | 1 |
| 12062 | Uncharacterized protein LOC109462197 isoform X3 [Branchiostoma belcheri] | Uncharacterized | 26 | 29 | 4 |
| 12758 | Hypothetical protein OCBIM_22013362mg [Octopus bimaculoides] | Uncharacterized | 5 | 2 | 1 |
| 6460 | Annexin A4-like [Octopus bimaculoides] | Protein-protein | 14 | 15 | 16 |
| 7073 | Peroxiredoxin-1-like [Octopus bimaculoides] | Metabolism | 31 | 25 | 19 |
| 3624 | PREDICTED: retinal dehydrogenase 1-like [Octopus bimaculoides] | Metabolism | 14 | 11 | 17 |
| 9264 | PREDICTED: glutathione S-transferase A-like [Octopus bimaculoides] | Metabolism | 25 | 25 | 8 |
| 11381 | PREDICTED: sarcoplasmic/endoplasmic reticulum calcium ATPase 1-like [Octopus bimaculoides] | Ion Transport | 3 | 2 | 14 |
| 7836 | Paramyosin | Cytoskeleton | 2 | 0 | 11 |
| 8583 | Uncharacterized protein LOC106879019 [Octopus bimaculoides] | Uncharacterized | 21 | 25 | 14 |
| 13274 | Laminin subunit gamma-1 | Extracellular Matrix | 2 | 3 | 4 |
| 3360 | Lysosomal aspartic protease-like isoform X2 [Octopus bimaculoides] | Metabolism | 16 | 17 | 9 |
| 7611 | Polyubiquitin-C [Chelonia mydas] | Metabolism | 26 | 29 | 37 |
| 11325 | Tetraspanin | Membrane | 38 | 41 | 31 |
| 2246 | PREDICTED: histone H2A [Octopus bimaculoides] | Nucleosome | 45 | 25 | 57 |
| 9625 | PREDICTED: transketolase-like protein 2 isoform X1 [Crassostrea gigas] | Metabolism | 8 | 15 | 0 |
| 2809 | PREDICTED: hemocyte protein-glutamine gamma-glutamyltransferase-like isoform X1 [Octopus bimaculoides] | Protein-Protein | 3 | 8 | 19 |
| 6218 | Ras-related protein Rab-1A [Haliotis discus discus] | Metabolism | 13 | 22 | 19 |
| 13237 | PREDICTED: laminin subunit alpha-like isoform X2 [Lingula anatina] | Extracellular Matrix | 0 | 3 | 1 |
| 13224 | S-crystallin SL20-1-like [Octopus bimaculoides] | Crystallin | 20 | 15 | 3 |
| 3081 | PREDICTED: ras-related protein Rab-32-like [Octopus bimaculoides] | Metabolism | 17 | 22 | 3 |
| 9551 | Belongs to the 3-beta-HSD family | Metabolism | 9 | 8 | 3 |
| 7400 | Sulfotransferase family cytosolic 1B member 1-like [Octopus bimaculoides] | Metabolism | 2 | 7 | 0 |
| 16456 | PREDICTED: hemocyte protein-glutamine gamma-glutamyltransferase-like isoform X2 [Octopus bimaculoides] | Protein-protein | 2 | 9 | 16 |
| 11257 | Calpain-B-like isoform X17 [Crassostrea virginica] | Metabolism | 6 | 4 | 3 |
| 8660 | Calcium ion binding | Metabolism | 1 | 6 | 2 |
| 12464 | PREDICTED: gelsolin-like protein 2 [Octopus bimaculoides] | Cytoskeleton | 15 | 11 | 10 |
| 2357 | Enolase [Doryteuthis pealeii] | Metabolism | 8 | 7 | 5 |
| 6430 | Uncharacterized protein LOC106874846 isoform X3 [Octopus bimaculoides] | Uncharacterized | 5 | 11 | 0 |
| 7486 | Histone H3 [Octopus bimaculoides] | Nucleosome | 24 | 14 | 31 |
| 5134 | Mitochondrial substrate/solute carrier | Membrane | 4 | 9 | 12 |
| 9016 | Membrane-associated protein Hem-like isoform X4 [Octopus bimaculoides] | Membrane | 0 | 7 | 1 |
| 9583 | Annexin A4-like [Crassostrea virginica] | Protein-Protein | 10 | 8 | 3 |
| 10856 | PREDICTED: alpha-adducin-like isoform X2 [Octopus bimaculoides] | Protein-Protein | 5 | 4 | 4 |
| 11850 | PREDICTED: ras-like GTP-binding protein RHO isoform X2 [Octopus bimaculoides] | Metabolism | 19 | 12 | 14 |
| 17820 | Uncharacterized protein LOC106873057 [Octopus bimaculoides] | Uncharacterized | 20 | 14 | 9 |
| 3933 | PREDICTED: retinal dehydrogenase 1-like [Octopus bimaculoides] | Metabolism | 5 | 12 | 0 |
| 8042 | Malate dehydrogenase | Metabolism | 10 | 9 | 6 |
| 9156 | PREDICTED: annexin A4-like [Octopus bimaculoides] | Protein-Protein | 9 | 9 | 4 |
| 13396 | PREDICTED: citrate synthase, mitochondrial-like [Octopus bimaculoides] | Metabolism | 9 | 7 | 3 |
| 5071 | Hypothetical protein BCR42DRAFT_419820 [Absidia repens] | Uncharacterized | 8 | 8 | 10 |
| 7013 | PREDICTED: flotillin-2-like [Octopus bimaculoides] | Membrane | 2 | 3 | 11 |
| 10059 | PREDICTED: fructose-bisphosphate aldolase-like [Octopus bimaculoides] | Metabolism | 9 | 9 | 6 |
| 11060 | Hypothetical protein LOTGIDRAFT_227290 [Lottia gigantea] | Uncharacterized | 7 | 11 | 11 |
| 12437 | PREDICTED: gelsolin-like protein 2 [Octopus bimaculoides] | Cytoskeleton | 9 | 11 | 0 |
| 19218 | ADP-ribosylation factor 1-like [Limulus polyphemus] | Metabolism | 11 | 11 | 13 |
| 789 | Histone H4 [Larimichthys crocea] | Nucleosome | 7 | 5 | 14 |
| 6642 | Annexin A4-like [Octopus bimaculoides] | Protein-protein | 10 | 3 | 3 |
| 9775 | PREDICTED: transaldolase-like [Octopus bimaculoides] | Metabolism | 8 | 10 | 1 |
| 11178 | Uncharacterized protein LOC106873394 [Octopus bimaculoides] | Uncharacterized | 8 | 14 | 8 |
| 13722 | Hypothetical protein OCBIM_22013356mg [Octopus bimaculoides] | Uncharacterized | 19 | 12 | 5 |
| 19621 | PREDICTED: puromycin-sensitive aminopeptidase-like [Octopus bimaculoides] | Metabolism | 1 | 1 | 5 |
| 2610 | PREDICTED: ras-like protein 3 isoform X1 [Octopus bimaculoides] | Metabolism | 12 | 14 | 2 |
| 6168 | Peroxiredoxin 6 protein [Sepiella maindroni] | Metabolism | 11 | 9 | 5 |
| 12463 | PREDICTED: uncharacterized protein LOC106880511 isoform X2 [Octopus bimaculoides] | Uncharacterized | 9 | 11 | 0 |
| 12766 | Hypothetical protein OCBIM_22013362mg [Octopus bimaculoides] | Uncharacterized | 2 | 2 | 0 |
| 16407 | Retrograde protein of 51 kDa [Biomphalaria glabrata] | Cytoskeleton | 11 | 7 | 33 |
| 4018 | PREDICTED: cdc42 homolog [Octopus bimaculoides] | Metabolism | 15 | 12 | 12 |
| 6452 | Lipase maturation factor 2-like isoform X2 [Octopus bimaculoides] | Metabolism | 2 | 9 | 3 |
| 7494 | PREDICTED: calpain-11-like [Octopus bimaculoides] | Metabolism | 1 | 0 | 7 |
| 8301 | PREDICTED: moesin-like [Octopus bimaculoides] | Protein-Protein | 3 | 3 | 3 |
| 10800 | Cystathionine gamma-lyase [Crassostrea gigas] | Metabolism | 8 | 7 | 0 |
| 11319 | PREDICTED: dolichyl-diphosphooligosaccharide--protein glycosyltransferase subunit 2-like [Octopus bimaculoides] | Metabolism | 3 | 5 | 5 |
| 17423 | Hypothetical protein LOTGIDRAFT_223383 [Lottia gigantea] | Uncharacterized | 14 | 10 | 2 |
| 19611 | PREDICTED: receptor expression-enhancing protein 5-like isoform X2 [Octopus bimaculoides] | Membrane | 22 | 18 | 7 |
| 61 | Tyrosine 3-monooxygenase/tryptophan 5-monooxygenase activation protein, epsilon polypeptide 2 | Metabolism | 6 | 5 | 8 |
| 4205 | PREDICTED: 78 kDa glucose-regulated protein-like [Octopus bimaculoides] | Metabolism | 3 | 2 | 4 |
| 5534 | peptidyl prolyl cis-trans isomerase A [Conus frigidus] | Metabolism | 19 | 14 | 0 |
| 6866 | PREDICTED: uncharacterized protein LOC106872046 [Octopus bimaculoides] | Uncharacterized | 8 | 7 | 1 |
| 9620 | PREDICTED: laminin subunit beta-1-like [Octopus bimaculoides] | Extracellular Matrix | 0 | 2 | 1 |
| 3161 | PREDICTED: flotillin-1-like isoform X1 [Aplysia californica] | Membrane | 0 | 2 | 8 |
| 5970 | nucleoredoxin-like protein 2 [Acanthaster planci] | Metabolism | 9 | 9 | 15 |
| 6668 | PREDICTED: pantetheinase-like [Octopus bimaculoides] | Metabolism | 2 | 2 | 7 |
| 7421 | PREDICTED: extended synaptotagmin-2-like isoform X1 [Octopus bimaculoides] | Membrane | 4 | 2 | 1 |
| 9133 | PREDICTED: uncharacterized protein LOC106876157 [Octopus bimaculoides] | Uncharacterized | 15 | 0 | 12 |
| 9154 | PREDICTED: rab GDP dissociation inhibitor alpha-like [Octopus bimaculoides] | Metabolism | 2 | 8 | 2 |
| 10684 | PREDICTED: NADP-dependent malic enzyme-like isoform X1 [Octopus bimaculoides] | Metabolism | 3 | 6 | 0 |
| 12448 | Clathrin heavy chain 1 isoform X2 [Mizuhopecten yessoensis] | Membrane | 1 | 1 | 1 |
| 13024 | PREDICTED: cytoplasmic dynein 1 heavy chain 1 isoform X12 [Crassostrea gigas] | Metabolism | 0 | 0 | 0 |
| 13169 | PREDICTED: cartilage matrix protein-like [Octopus bimaculoides] | Extracellular Matrix | 11 | 6 | 14 |
| 13720 | Hypothetical protein OCBIM_22013360mg [Octopus bimaculoides] | Uncharacterized | 22 | 12 | 0 |
| 17326 | PREDICTED: kynurenine formamidase [Chrysochloris asiatica] | Metabolism | 7 | 19 | 0 |
| 19928 | Hypothetical protein A1O3_08667 [Capronia epimyces CBS 606.96] | Uncharacterized | 4 | 8 | 10 |
| 1839 | PREDICTED: guanine nucleotide-binding protein G(o) subunit alpha isoform X1 [Octopus bimaculoides] | Metabolism | 4 | 5 | 4 |
| 6813 | Vacuolar protein sorting-associated protein 4B | Metabolism | 4 | 6 | 10 |
| 7691 | PREDICTED: transitional endoplasmic reticulum ATPase-like [Octopus bimaculoides] | Metabolism | 2 | 2 | 1 |
| 8860 | PREDICTED: hephaestin-like protein [Octopus bimaculoides] | Metabolism | 3 | 1 | 2 |
| 9165 | Reflectin-like protein A2 [Doryteuthis pealeii] | Reflectin | 7 | 14 | 14 |
| 11316 | Copine-3-like isoform X2 [Octopus bimaculoides] | Membrane | 5 | 9 | 9 |
| 13241 | Collagen alpha-4(VI) chain-like [Lingula anatina] | Extracellular Matrix | 0 | 5 | 8 |
| 19972 | PREDICTED: ATP synthase subunit O, mitochondrial-like [Octopus bimaculoides] | Metabolism | 5 | 5 | 7 |
| 20271 | PREDICTED: ATP synthase subunit g, mitochondrial-like [Octopus bimaculoides] | Metabolism | 15 | 15 | 20 |
| 3253 | PREDICTED: FAS-associated factor 2-like [Octopus bimaculoides] | Metabolism | 3 | 5 | 1 |
| 3329 | Reflectin-like protein A1 [Doryteuthis opalescens] | Reflectin | 5 | 9 | 7 |
| 5952 | PREDICTED: saccharopine dehydrogenase-like oxidoreductase isoform X1 [Pygocentrus nattereri] | Metabolism | 4 | 4 | 4 |
| 6281 | Uncharacterized protein LOC106874846 isoform X3 [Octopus bimaculoides] | Uncharacterized | 12 | 19 | 4 |
| 9052 | Membrane-associated protein Hem-like isoform X4 [Octopus bimaculoides] | Membrane | 2 | 5 | 0 |
| 9761 | PREDICTED: LOW QUALITY PROTEIN: dolichyl-diphosphooligosaccharide--protein glycosyltransferase 48 kDa subunit-like [Octopus bimaculoides] | Metabolism | 2 | 2 | 6 |
| 10007 | Syntenin-1-like [Octopus bimaculoides] | Protein-protein | 6 | 12 | 0 |
| 12583 | PREDICTED: gelsolin-like protein 2 [Octopus bimaculoides] | Cytoskeleton | 4 | 5 | 3 |
| 17618 | PREDICTED: nucleoside diphosphate kinase-like [Octopus bimaculoides] | Metabolism | 8 | 8 | 8 |
| 3386 | PREDICTED: dolichyl-diphosphooligosaccharide--protein glycosyltransferase subunit 1-like [Octopus bimaculoides] | Metabolism | 1 | 2 | 3 |
| 4186 | PREDICTED: serine/threonine-protein phosphatase 2A 65 kDa regulatory subunit A alpha isoform-like isoform X2 [Biomphalaria glabrata] | Metabolism | 2 | 4 | 1 |
| 4645 | PREDICTED: uncharacterized protein LOC106884239 [Octopus bimaculoides] | Uncharacterized | 5 | 3 | 0 |
| 5108 | Beta-tubulin [Doryteuthis pealeii] | Cytoskeleton | 4 | 3 | 4 |
| 5136 | PREDICTED: protocadherin-11 X-linked-like isoform X6 [Octopus bimaculoides] | Membrane | 4 | 7 | 0 |
| 6907 | PREDICTED: alpha-actinin, sarcomeric-like isoform X1 [Octopus bimaculoides] | Cytoskeleton | 1 | 0 | 2 |
| 7007 | Elongation factor 1-alpha | Metabolism | 3 | 5 | 0 |
| 7381 | PREDICTED: V-type proton ATPase catalytic subunit A [Octopus bimaculoides] | Ion Transport | 2 | 5 | 0 |
| 8039 | glutathione S-transferase 2 [Idiosepius paradoxus] | Metabolism | 7 | 12 | 0 |
| 8664 | PREDICTED: plasma membrane calcium-transporting ATPase 2-like [Octopus bimaculoides] | Ion Transport | 1 | 1 | 2 |
| 8671 | 6-phosphogluconate dehydrogenase, decarboxylating-like [Crassostrea virginica] | Metabolism | 2 | 6 | 0 |
| 9514 | PREDICTED: aquaporin-like [Octopus bimaculoides] | Membrane | 9 | 0 | 15 |
| 9844 | Peptidase C2, calpain, large subunit, domain III | Metabolism | 1 | 3 | 1 |
| 9929 | Heat shock protein 90 [Octopus vulgaris] | Metabolism | 1 | 1 | 2 |
| 11230 | Copine-3-like isoform X2 [Octopus bimaculoides] | Membrane | 4 | 5 | 1 |
| 16523 | PREDICTED: ATP synthase subunit b, mitochondrial-like [Octopus bimaculoides] | Metabolism | 3 | 6 | 3 |
| 19642 | PREDICTED: ras-related protein Rab-2 [Octopus bimaculoides] | Metabolism | 4 | 6 | 6 |
| 3686 | PREDICTED: glucose-6-phosphate 1-dehydrogenase-like [Octopus bimaculoides] | Metabolism | 1 | 5 | 0 |
| 5750 | Hypothetical protein LOTGIDRAFT_137310 [Lottia gigantea] | Uncharacterized | 6 | 8 | 0 |
| 7162 | PREDICTED: ras-related protein Rab-10-like [Aplysia californica] | Metabolism | 7 | 14 | 3 |
| 7265 | PREDICTED: profilin-like [Amphimedon queenslandica] | Cytoskeleton | 22 | 11 | 5 |
| 8371 | PREDICTED: uncharacterized protein LOC106870819 [Octopus bimaculoides] | Uncharacterized | 5 | 2 | 10 |
| 8515 | Isocitrate dehydrogenase [NADP] | Metabolism | 2 | 0 | 5 |
| 8780 | uncharacterized protein LOC106876168 [Octopus bimaculoides] | Uncharacterized | 0 | 1 | 6 |
| 9781 | Collagen, type IV | Extracellular Matrix | 1 | 1 | 1 |
| 11498 | PREDICTED: reticulon-1-A-like isoform X2 [Octopus bimaculoides] | Membrane | 10 | 7 | 7 |
| 17742 | Reflectin-like protein A1 [Doryteuthis pealeii] | Reflectin | 4 | 7 | 15 |
| 3203 | PREDICTED: prosaposin-like isoform X2 [Octopus bimaculoides] | Metabolism | 2 | 1 | 1 |
| 3763 | Hypothetical protein OCBIM_22016971mg [Octopus bimaculoides] | Uncharacterized | 20 | 7 | 13 |
| 5585 | PREDICTED: protocadherin-11 X-linked-like isoform X6 [Octopus bimaculoides] | Membrane | 4 | 6 | 2 |
| 8144 | Hypothetical protein LOTGIDRAFT_231723 [Lottia gigantea] | Uncharacterized | 1 | 3 | 0 |
| 8255 | PREDICTED: aspartate aminotransferase, mitochondrial-like [Octopus bimaculoides] | Metabolism | 2 | 0 | 4 |
| 8594 | Transmembrane emp24 domain-containing protein 10-like [Mizuhopecten yessoensis] | Protein-Protein | 4 | 4 | 4 |
| 8828 | Uncharacterized protein LOC106879019 [Octopus bimaculoides] | Uncharacterized | 1 | 2 | 1 |
| 9592 | PREDICTED: von Willebrand factor A domain-containing protein 5A-like [Octopus bimaculoides] | Uncharacterized | 2 | 2 | 0 |
| 10106 | Vitellinogen, open beta-sheet | Metabolism | 0 | 0 | 1 |
| 10692 | PREDICTED: vinculin-like [Octopus bimaculoides] | Cytoskeleton | 1 | 1 | 1 |
| 12147 | PREDICTED: smoothelin-like protein 1 [Octopus bimaculoides] | Protein-protein | 6 | 3 | 9 |
| 12975 | PREDICTED: neural cell adhesion molecule 2-like [Octopus bimaculoides] | Membrane | 0 | 5 | 0 |
| 21163 | PREDICTED: putative ATP synthase subunit f, mitochondrial [Octopus bimaculoides] | Membrane | 6 | 6 | 6 |
| 450 | Gq-alpha [Doryteuthis pealeii] | Metabolism | 4 | 3 | 0 |
| 521 | PREDICTED: putative ATP synthase subunit f, mitochondrial [Octopus bimaculoides] | Metabolism | 7 | 3 | 7 |
| 565 | PREDICTED: ribosyldihydronicotinamide dehydrogenase [quinone]-like [Octopus bimaculoides] | Metabolism | 2 | 10 | 0 |
| 1273 | PREDICTED: zinc finger Ran-binding domain-containing protein 2-like [Octopus bimaculoides] | Protein-Protein | 2 | 2 | 5 |
| 3265 | PREDICTED: V-type proton ATPase 16 kDa proteolipid subunit [Octopus bimaculoides] | Ion Transport | 11 | 16 | 0 |
| 3746 | PREDICTED: retinal dehydrogenase 1-like [Octopus bimaculoides] | Metabolism | 0 | 3 | 2 |
| 4048 | PREDICTED: glycerol-3-phosphate dehydrogenase, mitochondrial-like isoform X1 [Octopus bimaculoides] | Metabolism | 0 | 1 | 2 |
| 4858 | Actin depolymerizing protein [Gonapodya prolifera JEL478] | Metabolism | 5 | 3 | 5 |
| 6044 | PREDICTED: endoplasmin-like [Octopus bimaculoides] | Metabolism | 1 | 2 | 1 |
| 6347 | PREDICTED: V-type proton ATPase subunit B [Octopus bimaculoides] | Ion Transport | 1 | 4 | 0 |
| 6702 | PREDICTED: putative universal stress protein SAS1637 [Octopus bimaculoides] | Metabolism | 12 | 8 | 0 |
| 7247 | Elongation factor Tu [Tenacibaculum dicentrarchi] | Metabolism | 0 | 0 | 7 |
| 7384 | PREDICTED: stomatin-like protein 2, mitochondrial [Octopus bimaculoides] | Membrane | 4 | 3 | 0 |
| 7407 | PREDICTED: GTPase HRas isoform X2 [Octopus bimaculoides] | Metabolism | 8 | 5 | 0 |
| 8509 | PREDICTED: probable phosphoglycerate mutase [Octopus bimaculoides] | Metabolism | 1 | 5 | 0 |
| 8706 | Uncharacterized protein LOC106879019 [Octopus bimaculoides] | Uncharacterized | 2 | 2 | 1 |
| 8855 | PREDICTED: retinol dehydrogenase 12-like [Octopus bimaculoides] | Metabolism | 3 | 0 | 4 |
| 9091 | PREDICTED: zinc metalloproteinase nas-15-like [Aplysia californica] | Metabolism | 2 | 0 | 1 |
| 10030 | Talin-1-like isoform X21 [Crassostrea virginica] | Cytoskeleton | 0 | 0 | 0 |
| 10086 | PREDICTED: dolichyl-diphosphooligosaccharide--protein glycosyltransferase subunit STT3A isoform X1 [Octopus bimaculoides] | Metabolism | 1 | 1 | 3 |
| 10557 | Adenylyl cyclase-associated protein 1-like isoform X3 [Crassostrea virginica] | Metabolism | 1 | 3 | 0 |
| 11283 | PREDICTED: glutamate dehydrogenase, mitochondrial-like [Octopus bimaculoides] | Metabolism | 2 | 1 | 2 |
| 11801 | PREDICTED: disco-interacting protein 2 homolog A-like [Octopus bimaculoides] | Membrane | 0 | 0 | 2 |
| 12681 | PREDICTED: gelsolin-like protein 2 [Octopus bimaculoides] | Cytoskeleton | 2 | 1 | 2 |
| 13083 | PREDICTED: cytochrome b-c1 complex subunit 2, mitochondrial-like isoform X2 [Octopus bimaculoides] | Metabolism | 0 | 3 | 4 |
| 13126 | PREDICTED: gelsolin-like protein 2 [Octopus bimaculoides] | Cytoskeleton | 0 | 0 | 1 |
| 16776 | Reflectin-like protein C1 [Doryteuthis opalescens] | Reflectin | 0 | 3 | 11 |
| 17985 | PREDICTED: ATP-dependent RNA helicase eIF4A-like [Octopus bimaculoides] | Nucleosome | 2 | 3 | 0 |
| 18701 | PREDICTED: probable caffeoyl-CoA O-methyltransferase 1 [Octopus bimaculoides] | Metabolism | 4 | 3 | 0 |
| 266 | PREDICTED: excitatory amino acid transporter 1-like [Octopus bimaculoides] | Ion Transport | 2 | 2 | 5 |
| 3058 | PREDICTED: myosin catalytic light chain LC-1, mantle muscle-like [Octopus bimaculoides] | Metabolism | 3 | 0 | 9 |
| 3062 | PREDICTED: phytanoyl-CoA dioxygenase, peroxisomal-like isoform X1 [Octopus bimaculoides] | Metabolism | 3 | 0 | 9 |
| 3231 | PREDICTED: deoxynucleoside triphosphate triphosphohydrolase SAMHD1-like [Octopus bimaculoides] | Metabolism | 1 | 1 | 2 |
| 3372 | PREDICTED: CD81 protein-like isoform X1 [Octopus bimaculoides] | Membrane | 8 | 8 | 0 |
| 3962 | Hypothetical protein CGI_10001848 [Crassostrea gigas] | Uncharacterized | 0 | 0 | 7 |
| 4948 | PREDICTED: transmembrane protein 245-like [Octopus bimaculoides] | Membrane | 1 | 3 | 0 |
| 5009 | Protein kinase C and casein kinase substrate in neurons protein 1-like [Octopus bimaculoides] | Metabolism | 7 | 7 | 0 |
| 5888 | PREDICTED: NAD(P) transhydrogenase, mitochondrial-like [Aplysia californica] | Metabolism | 1 | 2 | 1 |
| 6375 | Defender against cell death 1 | Membrane | 13 | 0 | 13 |
| 7113 | PREDICTED: GTP-binding protein SAR1b-like [Octopus bimaculoides] | Metabolism | 4 | 2 | 2 |
| 7276 | PREDICTED: malate dehydrogenase, cytoplasmic-like [Octopus bimaculoides] | Metabolism | 5 | 2 | 0 |
| 7430 | PREDICTED: serine/threonine-protein phosphatase alpha-2 isoform isoform X3 [Octopus bimaculoides] | Protein-Protein | 3 | 3 | 0 |
| 8572 | Cluster: Flagellar biosynthetic protein FlhB | Cytoskeleton | 3 | 0 | 9 |
| 9441 | PREDICTED: GLIPR1-like protein 2 [Octopus bimaculoides] | Metabolism | 1 | 1 | 2 |
| 10045 | V-type proton ATPase 116 kDa subunit a-like isoform X2 [Limulus polyphemus] | Ion Transport | 2 | 2 | 0 |
| 11310 | Low-density lipoprotein receptor-related protein 2-like [Parasteatoda tepidariorum] | Membrane | 4 | 9 | 4 |
| 11350 | PREDICTED: catenin beta-like [Octopus bimaculoides] | Protein-Protein | 1 | 0 | 2 |
| 16300 | PREDICTED: 40S ribosomal protein S4-like [Octopus bimaculoides] | Metabolism | 4 | 1 | 0 |
| 18985 | PREDICTED: 60S ribosomal protein L14-like isoform X1 [Octopus bimaculoides] | Metabolism | 5 | 0 | 5 |
| 20501 | Tyrosinase-like [Octopus bimaculoides] | Metabolism | 7 | 3 | 3 |
| 425 | PREDICTED: tryptophan 2,3-dioxygenase-like [Lingula anatina] | Metabolism | 2 | 5 | 0 |
| 1801 | Hypothetical protein OCBIM_22013360mg [Octopus bimaculoides] | Uncharacterized | 7 | 4 | 0 |
| 2347 | Iduronate 2-sulfatase-like [Octopus bimaculoides] | Metabolism | 3 | 2 | 0 |
| 2714 | PREDICTED: actin-related protein 2/3 complex subunit 4 [Octopus bimaculoides] | Cytoskeleton | 4 | 2 | 0 |
| 3066 | PREDICTED: putative aminopeptidase W07G4.4 [Octopus bimaculoides] | Metabolism | 1 | 2 | 0 |
| 3384 | PREDICTED: pyruvate dehydrogenase E1 component subunit alpha, mitochondrial-like [Octopus bimaculoides] | Metabolism | 1 | 2 | 0 |
| 4418 | PREDICTED: abhydrolase domain-containing protein 16A-like isoform X1 [Octopus bimaculoides] | Metabolism | 1 | 1 | 1 |
| 4636 | Sodium-calcium exchanger [Doryteuthis pealeii] | Ion Transport | 3 | 0 | 6 |
| 5143 | Translocon-associated protein subunit beta | Metabolism | 2 | 0 | 5 |
| 5469 | Matrilin 2 | Extracellular Matrix | 1 | 0 | 2 |
| 5721 | PREDICTED: ADP-ribosylation factor-like protein 8B [Octopus bimaculoides] | Metabolism | 2 | 2 | 2 |
| 5843 | PREDICTED: acyl-coenzyme A thioesterase 9, mitochondrial-like [Branchiostoma belcheri] | Metabolism | 1 | 2 | 0 |
| 6680 | PREDICTED: collagen alpha-4(VI) chain-like [Octopus bimaculoides] | Extracellular Matrix | 0 | 0 | 1 |
| 7375 | PREDICTED: large neutral amino acids transporter small subunit 2-like isoform X1 [Octopus | Metabolism | 2 | 2 | 2 |
| 7942 | Calmodulin [Mucor circinelloides f. circinelloides 1006PhL] | Protein-Protein | 5 | 0 | 3 |
| 8360 | Annexin A13-like isoform X2 [Octopus bimaculoides] | Protein-protein | 1 | 2 | 0 |
| 8518 | PREDICTED: nicastrin-like isoform X1 [Octopus bimaculoides] | Metabolism | 2 | 1 | 0 |
| 9076 | Thioester-containing protein 1 [Euprymna scolopes] | Protein-Protein | 0 | 0 | 1 |
| 9821 | Prohibitin 2 [Sepiella japonica] | Metabolism | 1 | 1 | 1 |
| 10121 | PREDICTED: copine-3-like [Octopus bimaculoides] | Membrane | 1 | 0 | 2 |
| 10973 | Histidine ammonia-lyase-like [Mizuhopecten yessoensis] | Metabolism | 0 | 0 | 2 |
| 11141 | PREDICTED: integrin alpha pat-2-like [Octopus bimaculoides] | Membrane | 1 | 0 | 0 |
| 11142 | PREDICTED: ornithine aminotransferase, mitochondrial-like isoform X1 [Octopus bimaculoides] | Metabolism | 1 | 3 | 0 |
| 11219 | CBN-EMB-9 protein | Protein-protein | 0 | 1 | 0 |
| 11499 | PREDICTED: reticulon-1-A isoform X5 [Crassostrea gigas] | Membrane | 9 | 9 | 9 |
| 11502 | PREDICTED: reticulon-1-A-like isoform X3 [Octopus bimaculoides] | Membrane | 3 | 3 | 3 |
| 11503 | PREDICTED: reticulon-1-A isoform X9 [Crassostrea gigas] | Membrane | 4 | 4 | 4 |
| 11655 | Low-density lipoprotein receptor-related protein 2-like [Parasteatoda tepidariorum] | Membrane | 1 | 2 | 0 |
| 12660 | NADH-cytochrome b5 reductase 3-like isoform X1 [Crassostrea virginica] | Metabolism | 2 | 2 | 2 |
| 13273 | PREDICTED: copine-8-like [Octopus bimaculoides] | Membrane | 1 | 1 | 1 |
| 16209 | PREDICTED: ganglioside GM2 activator-like [Octopus bimaculoides] | Protein-protein | 0 | 6 | 0 |
| 16329 | PREDICTED: focadhesin-like [Octopus bimaculoides] | Membrane | 8 | 0 | 16 |
| 16614 | PREDICTED: ectonucleoside triphosphate diphosphohydrolase 1-like [Octopus bimaculoides] | Membrane | 2 | 4 | 0 |
| 16835 | PREDICTED: uncharacterized protein LOC106877008 [Octopus bimaculoides] | Uncharacterized | 4 | 4 | 4 |
| 17881 | PREDICTED: ATP synthase subunit d, mitochondrial-like [Octopus bimaculoides] | Metabolism | 7 | 3 | 0 |
| 17993 | PREDICTED: adenosine deaminase-like [Octopus bimaculoides] | Metabolism | 1 | 2 | 0 |
| 18123 | Ferritin [Sepiella maindroni] | Metabolism | 3 | 5 | 0 |
| 18922 | Uncharacterized protein LOC106873057 [Octopus bimaculoides] | Uncharacterized | 7 | 3 | 0 |
| 21123 | PREDICTED: synaptophysin-like isoform X1 [Octopus bimaculoides] | Protein-Protein | 6 | 3 | 0 |
| 335 | PREDICTED: transmembrane emp24 domain-containing protein 2 [Crassostrea gigas] | Protein-Protein | 5 | 0 | 0 |
| 366 | PREDICTED: NADH dehydrogenase [ubiquinone] 1 subunit C2 [Cephus cinctus] | Metabolism | 4 | 0 | 4 |
| 672 | PREDICTED: protein Mo25-like [Octopus bimaculoides] | Metabolism | 1 | 1 | 0 |
| 1078 | PREDICTED: mesoderm-specific transcript homolog protein-like [Octopus bimaculoides] | Metabolism | 6 | 6 | 0 |
| 2064 | PREDICTED: protein deglycase DJ-1-like isoform X1 [Priapulus caudatus] | Metabolism | 4 | 0 | 0 |
| 2454 | PREDICTED: elongation factor 1-gamma-like [Octopus bimaculoides] | Metabolism | 0 | 4 | 0 |
| 2573 | PREDICTED: vesicular integral-membrane protein VIP36-like [Octopus bimaculoides] | Protein-Protein | 0 | 2 | 2 |
| 3209 | PREDICTED: acylamino-acid-releasing enzyme-like [Octopus bimaculoides] | Metabolism | 1 | 0 | 1 |
| 4004 | Chain A, Crystal Structure Analysis Of Neuronal Sec1 From The Squid L. Pealei | Membrane | 1 | 1 | 0 |
| 4141 | PREDICTED: beta-hexosaminidase subunit beta-like [Octopus bimaculoides] | Metabolism | 1 | 0 | 1 |
| 4191 | PREDICTED: actophorin-like [Octopus bimaculoides] | Metabolism | 3 | 3 | 0 |
| 4200 | PREDICTED: 78 kDa glucose-regulated protein-like [Octopus bimaculoides] | Metabolism | 2 | 0 | 2 |
| 4531 | PREDICTED: uncharacterized protein LOC106874881 [Octopus bimaculoides] | Uncharacterized | 3 | 0 | 3 |
| 4749 | PREDICTED: calcium-binding mitochondrial carrier protein SCaMC-2-like [Octopus bimaculoides] | Ion Transport | 0 | 2 | 0 |
| 5069 | Uncharacterized | Uncharacterized | 5 | 5 | 0 |
| 5145 | PREDICTED: uncharacterized protein LOC106876157 [Octopus bimaculoides] | Uncharacterized | 7 | 0 | 0 |
| 5445 | PREDICTED: ras-related protein Rab-5B-like [Octopus bimaculoides] | Metabolism | 2 | 2 | 0 |
| 5459 | Low-density lipoprotein receptor-related protein 2-like [Parasteatoda tepidariorum] | Membrane | 0 | 3 | 3 |
| 5460 | Low-density lipoprotein receptor-related protein 2-like [Parasteatoda tepidariorum] | Membrane | 0 | 3 | 3 |
| 6049 | PREDICTED: cathepsin L1-like [Octopus bimaculoides] | Metabolism | 1 | 1 | 0 |
| 6051 | PREDICTED: 26S protease regulatory subunit 6B [Octopus bimaculoides] | Metabolism | 1 | 1 | 0 |
| 6453 | PREDICTED: 40S ribosomal protein S5 [Octopus bimaculoides] | Metabolism | 2 | 0 | 2 |
| 6508 | Hypothetical protein OCBIM_22013360mg [Octopus bimaculoides] | Uncharacterized | 5 | 0 | 0 |
| 6589 | PREDICTED: triosephosphate isomerase-like [Octopus bimaculoides] | Metabolism | 2 | 2 | 0 |
| 6809 | PREDICTED: wiskott-Aldrich syndrome protein family member 3-like [Octopus bimaculoides] | Protein-Protein | 0 | 4 | 0 |
| 6810 | PREDICTED: alpha-N-acetylgalactosaminidase-like [Octopus bimaculoides] | Metabolism | 2 | 2 | 0 |
| 7324 | Ubiquitin-conjugating enzyme E2 L3-like [Limulus polyphemus] | Metabolism | 0 | 6 | 0 |
| 7522 | PREDICTED: T-cell immunomodulatory protein-like [Octopus bimaculoides] | Protein-Protein | 1 | 1 | 0 |
| 7534 | PREDICTED: translocon-associated protein subunit gamma-like [Octopus bimaculoides] | Metabolism | 4 | 0 | 4 |
| 7644 | PREDICTED: ubiquitin carboxyl-terminal hydrolase-like [Crassostrea gigas] | Metabolism | 2 | 2 | 0 |
| 7681 | PREDICTED: glycogen phosphorylase, brain form-like [Octopus bimaculoides] | Metabolism | 1 | 1 | 0 |
| 7725 | PREDICTED: aspartyl/asparaginyl beta-hydroxylase-like [Octopus bimaculoides] | Metabolism | 5 | 0 | 0 |
| 7804 | PREDICTED: surfeit locus protein 4-like [Octopus bimaculoides] | Membrane | 2 | 0 | 2 |
| 7852 | PREDICTED: 2-oxoglutarate dehydrogenase, mitochondrial-like isoform X2 [Octopus bimaculoides] | Metabolism | 1 | 1 | 0 |
| 7885 | PREDICTED: membrane-associated progesterone receptor component 1-like [Octopus bimaculoides] | Membrane | 2 | 2 | 0 |
| 8081 | Hypothetical protein OCBIM_22013360mg [Octopus bimaculoides] | Uncharacterized | 4 | 0 | 0 |
| 8804 | PREDICTED: lamin-B1-like [Octopus bimaculoides] | Cytoskeleton | 1 | 0 | 0 |
| 9092 | Spectrin beta chain | Cytoskeleton | 0 | 2 | 0 |
| 9258 | PREDICTED: retinol dehydrogenase 11-like isoform X2 [Octopus bimaculoides] | Metabolism | 1 | 0 | 1 |
| 9340 | Hypothetical protein OCBIM_22013360mg [Octopus bimaculoides] | Uncharacterized | 5 | 5 | 0 |
| 9552 | PREDICTED: acid ceramidase-like [Octopus bimaculoides] | Metabolism | 1 | 1 | 0 |
| 9689 | PREDICTED: dihydropyrimidinase-like [Octopus bimaculoides] | Metabolism | 0 | 2 | 0 |
| 9951 | PREDICTED: solute carrier family 2, facilitated glucose transporter member 1-like [Octopus bimaculoides] | Membrane | 2 | 2 | 0 |
| 10429 | PREDICTED: 60 kDa heat shock protein, mitochondrial-like [Octopus bimaculoides] | Metabolism | 1 | 1 | 0 |
| 10788 | PREDICTED: UDP-glucose:glycoprotein glucosyltransferase 1-like [Octopus bimaculoides] | Metabolism | 0 | 0 | 0 |
| 11366 | Hypothetical protein OCBIM_22002744mg [Octopus bimaculoides] | Uncharacterized | 0 | 1 | 1 |
| 11501 | Ribonuclease E [Pseudoalteromonas sp. PAMC 28425] | Metabolism | 3 | 0 | 3 |
| 13113 | PREDICTED: basement membrane-specific heparan sulfate proteoglycan core protein-like isoform X4 [Octopus bimaculoides] | Extracellular Matrix | 0 | 3 | 3 |
| 13761 | Chain A, Diisopropyl Fluorophosphatase (Dfpase), D121e Mutant [Loligo vulgaris] | Metabolism | 5 | 5 | 0 |
| 16191 | PREDICTED: sialin-like [Octopus bimaculoides] | Membrane | 2 | 2 | 0 |
| 16374 | PREDICTED: 40S ribosomal protein S3-B-like [Octopus bimaculoides] | Metabolism | 3 | 0 | 0 |
| 16868 | PREDICTED: erlin-1-like [Octopus bimaculoides] | Protein-Protein | 4 | 0 | 0 |
| 17024 | PREDICTED: 60S ribosomal protein L13a-like [Octopus bimaculoides] | Metabolism | 3 | 0 | 0 |
| 17150 | Similar to Drosophila melanogaster RpS3A [Drosophila yakuba] | Metabolism | 5 | 0 | 0 |
| 17604 | Cysteine protease ATG4B-like [Crassostrea virginica] | Metabolism | 2 | 2 | 0 |
| 17672 | PREDICTED: nucleoside diphosphate kinase-like [Octopus bimaculoides] | Metabolism | 1 | 1 | 0 |
| 17897 | PREDICTED: AP-1 complex subunit beta-1-like isoform X3 [Octopus bimaculoides] | Protein-Protein | 1 | 1 | 0 |
| 17900 | PREDICTED: 40S ribosomal protein S8-like [Octopus bimaculoides] | Metabolism | 2 | 0 | 2 |
| 17939 | Copper zinc superoxide dismutase [Sepiella maindroni] | Metabolism | 0 | 7 | 0 |
| 18329 | 40S ribosomal protein S18 [Exaiptasia pallida] | Metabolism | 2 | 0 | 2 |
| 19124 | PREDICTED: hydroxysteroid 11-beta-dehydrogenase 1-like protein [Octopus bimaculoides] | Metabolism | 0 | 0 | 5 |
| 19871 | PREDICTED: ras-related protein Rab-11A [Lingula anatina] | Metabolism | 2 | 2 | 0 |
| 20007 | PREDICTED: calumenin-A-like [Octopus bimaculoides] | Metabolism | 2 | 0 | 0 |
| 20325 | PREDICTED: 40S ribosomal protein S16 [Octopus bimaculoides] | Metabolism | 4 | 0 | 0 |
| 20347 | PREDICTED: peptidyl-prolyl cis-trans isomerase B-like [Octopus bimaculoides] | Metabolism | 2 | 0 | 2 |
| 98 | PREDICTED: minor histocompatibility antigen H13-like isoform X1 [Octopus bimaculoides] | Membrane | 0 | 0 | 2 |
| 131 | PREDICTED: mitochondrial carrier homolog 2-like [Octopus bimaculoides] | Ion Transport | 0 | 2 | 0 |
| 306 | PREDICTED: pyruvate dehydrogenase E1 component subunit beta, mitochondrial-like [Octopus bimaculoides] | Metabolism | 2 | 0 | 0 |
| 320 | PREDICTED: maestro heat-like repeat-containing protein family member 1 [Octopus bimaculoides] | Membrane | 3 | 0 | 0 |
| 362 | PREDICTED: 60S ribosomal protein L27-like [Octopus bimaculoides] | Metabolism | 3 | 0 | 0 |
| 641 | PREDICTED: LETM1 and EF-hand domain-containing protein 1, mitochondrial-like isoform X2 [Octopus bimaculoides] | Ion Transport | 0 | 0 | 0 |
| 696 | PREDICTED: cleft lip and palate transmembrane protein 1 homolog [Octopus bimaculoides] | Metabolism | 1 | 0 | 0 |
| 717 | PREDICTED: heparanase-like [Octopus bimaculoides] | Extracellular Matrix | 1 | 0 | 0 |
| 985 | PREDICTED: ras-related protein Rab-18A-like isoform X2 [Octopus bimaculoides] | Metabolism | 2 | 0 | 0 |
| 1037 | PREDICTED: erlin-1-like [Octopus bimaculoides] | Protein-Protein | 6 | 0 | 0 |
| 1527 | Octopine dehydrogenase [Doryteuthis opalescens] | Metabolism | 0 | 1 | 0 |
| 1882 | PREDICTED: digestive cysteine proteinase 2-like [Lingula anatina] | Metabolism | 1 | 0 | 0 |
| 1989 | PREDICTED: mitochondrial pyruvate carrier 2-like [Octopus bimaculoides] | Ion Transport | 0 | 0 | 4 |
| 2213 | PREDICTED: 60S ribosomal protein L15-like [Octopus bimaculoides] | Metabolism | 2 | 0 | 0 |
| 2607 | PREDICTED: cytochrome c oxidase subunit 5B, mitochondrial-like [Octopus bimaculoides] | Metabolism | 3 | 0 | 0 |
| 2727 | Translationally controlled tumor-associated [Rhizopus microsporus var. microsporus] | Protein-Protein | 0 | 3 | 0 |
| 3393 | Glutathione-S-transferase [Haliotis madaka] | Metabolism | 2 | 0 | 0 |
| 3433 | PREDICTED: signal recognition particle receptor subunit beta-like [Octopus bimaculoides] | Membrane | 2 | 0 | 0 |
| 3567 | PREDICTED: T-complex protein 1 subunit beta-like [Octopus bimaculoides] | Metabolism | 1 | 0 | 0 |
| 3895 | PREDICTED: uncharacterized protein LOC106868120 [Octopus bimaculoides] | Uncharacterized | 0 | 1 | 0 |
| 4211 | PREDICTED: uncharacterized protein LOC106882100 [Octopus bimaculoides] | Uncharacterized | 1 | 0 | 0 |
| 4247 | PREDICTED: transmembrane emp24 domain-containing protein eca isoform X1 [Crassostrea gigas] | Protein-Protein | 0 | 0 | 2 |
| 4251 | PREDICTED: glyceraldehyde-3-phosphate dehydrogenase-like isoform X1 [Octopus bimaculoides] | Metabolism | 0 | 0 | 1 |
| 4370 | PREDICTED: succinyl-CoA ligase subunit alpha, mitochondrial-like [Octopus bimaculoides] | Metabolism | 0 | 0 | 2 |
| 4407 | PREDICTED: uncharacterized threonine-rich GPI-anchored glycoprotein PJ4664.02-like [Octopus bimaculoides] | Uncharacterized | 0 | 2 | 0 |
| 4834 | Hypothetical protein OCBIM_22030393mg [Octopus bimaculoides] | Uncharacterized | 4 | 0 | 0 |
| 4918 | PREDICTED: acetolactate synthase-like protein [Octopus bimaculoides] | Metabolism | 0 | 2 | 0 |
| 5046 | PREDICTED: 60S ribosomal protein L28-like [Octopus bimaculoides] | Metabolism | 3 | 0 | 0 |
| 5056 | PREDICTED: trafficking protein particle complex subunit 12-like [Octopus bimaculoides] | Protein-Protein | 0 | 0 | 1 |
| 5325 | PREDICTED: opioid growth factor receptor-like protein 1 isoform X2 [Octopus bimaculoides] | Protein-Protein | 2 | 0 | 0 |
| 5334 | Hypothetical protein OCBIM_22013360mg [Octopus bimaculoides] | Uncharacterized | 4 | 0 | 0 |
| 5396 | PREDICTED: uncharacterized protein LOC106883080 [Octopus bimaculoides] | Uncharacterized | 0 | 0 | 0 |
| 5524 | LOW QUALITY PROTEIN: annexin A4-like [Crassostrea virginica] | Protein-Protein | 1 | 0 | 0 |
| 5634 | PREDICTED: heterogeneous nuclear ribonucleoprotein D-like-A isoform X1 [Octopus bimaculoides] | Metabolism | 2 | 0 | 0 |
| 5636 | PREDICTED: LOW QUALITY PROTEIN: eukaryotic translation initiation factor 3 subunit K-like [Octopus bimaculoides] | Metabolism | 2 | 0 | 0 |
| 5689 | Endothelin-converting enzyme 1 [Crassostrea gigas] | Metabolism | 1 | 0 | 0 |
| 5704 | PREDICTED: WW domain-binding protein 2-like [Octopus bimaculoides] | Protein-protein | 0 | 3 | 0 |
| 5751 | PREDICTED: MICOS complex subunit MIC13-like [Octopus bimaculoides] | Membrane | 0 | 0 | 5 |
| 5834 | PREDICTED: microsomal triglyceride transfer protein large subunit-like [Octopus bimaculoides] | Metabolism | 0 | 0 | 1 |
| 5855 | Uncharacterized | Uncharacterized | 1 | 0 | 0 |
| 5857 | PREDICTED: uncharacterized protein LOC106874393 isoform X7 [Octopus bimaculoides] | Uncharacterized | 0 | 0 | 0 |
| 6213 | Glutamine synthetase [Tegillarca granosa] | Metabolism | 1 | 0 | 0 |
| 6327 | PREDICTED: mitochondrial pyruvate carrier 1-like isoform X1 [Octopus bimaculoides] | Ion Transport | 0 | 0 | 3 |
| 6359 | Hypothetical protein OCBIM_22031876mg [Octopus bimaculoides] | Uncharacterized | 3 | 0 | 0 |
| 6707 | Ribonuclease E [Idiomarina donghaiensis] | Metabolism | 4 | 0 | 0 |
| 6874 | PREDICTED: actin-related protein 3 [Lingula anatina] | Cytoskeleton | 1 | 0 | 0 |
| 7189 | Sulfotransferase family cytosolic 1B member 1-like [Octopus bimaculoides] | Metabolism | 1 | 0 | 0 |
| 7243 | PREDICTED: importin-4-like [Octopus bimaculoides] | Membrane | 1 | 0 | 0 |
| 7250 | PREDICTED: 26S proteasome non-ATPase regulatory subunit 2-like [Octopus bimaculoides] | Metabolism | 1 | 0 | 0 |
| 7358 | Elongation of very long-chain fatty acids protein [Sepia officinalis] | Metabolism | 0 | 0 | 2 |
| 7560 | PREDICTED: uncharacterized protein LOC106872137 [Octopus bimaculoides] | Uncharacterized | 0 | 6 | 0 |
| 7636 | PREDICTED: CD9 antigen-like [Octopus bimaculoides] | Membrane | 3 | 0 | 0 |
| 7661 | PREDICTED: V-type proton ATPase subunit C 1-A-like [Octopus bimaculoides] | Ion Transport | 0 | 1 | 0 |
| 7700 | PREDICTED: alpha-L-fucosidase-like isoform X1 [Octopus bimaculoides] | Metabolism | 0 | 1 | 0 |
| 7926 | PREDICTED: retinol dehydrogenase 3-like [Octopus bimaculoides] | Metabolism | 1 | 0 | 0 |
| 7930 | PREDICTED: calpain-9-like [Aplysia californica] | Metabolism | 0 | 0 | 1 |
| 8024 | PREDICTED: B-cell receptor-associated protein 31-like [Octopus bimaculoides] | Membrane | 0 | 4 | 0 |
| 8068 | PREDICTED: alpha-aminoadipic semialdehyde dehydrogenase-like [Octopus bimaculoides] | Metabolism | 1 | 0 | 0 |
| 8167 | PREDICTED: MICOS complex subunit Mic60-like [Octopus bimaculoides] | Membrane | 0 | 0 | 1 |
| 8211 | Estrogen receptor [Sepiella maindroni] | Metabolism | 0 | 0 | 1 |
| 8297 | PREDICTED: vacuolar protein sorting-associated protein 13A-like [Octopus bimaculoides] | Metabolism | 0 | 0 | 0 |
| 8391 | PREDICTED: dystroglycan-like [Octopus bimaculoides] | Membrane | 0 | 1 | 0 |
| 8480 | PREDICTED: aconitate hydratase, mitochondrial-like [Octopus bimaculoides] | Metabolism | 0 | 0 | 1 |
| 8504 | PREDICTED: ninjurin-2-like isoform X1 [Octopus bimaculoides] | Membrane | 5 | 0 | 0 |
| 8601 | PREDICTED: NADH dehydrogenase [ubiquinone] 1 alpha subcomplex subunit 9, mitochondrial-like [Octopus bimaculoides] | Metabolism | 1 | 0 | 0 |
| 8635 | Uncharacterized protein LOC106879019 [Octopus bimaculoides] | Uncharacterized | 1 | 0 | 0 |
| 8652 | PREDICTED: actin-related protein 2/3 complex subunit 5-like [Octopus bimaculoides] | Cytoskeleton | 0 | 3 | 0 |
| 8879 | PREDICTED: beta-lactamase domain-containing protein 2-like [Octopus bimaculoides] | Metabolism | 0 | 0 | 1 |
| 8988 | Chain A, Neuronal Complexin SNARE COMPLEX [Loligo Pealei] | Protein-Protein | 4 | 0 | 0 |
| 9022 | PREDICTED: NADH dehydrogenase [ubiquinone] flavoprotein 2, mitochondrial-like [Octopus bimaculoides] | Metabolism | 0 | 0 | 2 |
| 9059 | Hypothetical protein OCBIM_22029476mg [Octopus bimaculoides] | Uncharacterized | 0 | 0 | 0 |
| 9223 | PREDICTED: fatty acid-binding protein 2, liver-like [Octopus bimaculoides] | Metabolism | 3 | 0 | 0 |
| 9293 | PREDICTED: histone H1-delta-like [Priapulus caudatus] | Nucleosome | 4 | 0 | 0 |
| 9333 | PREDICTED: actin-interacting protein 1-like [Octopus bimaculoides] | Metabolism | 1 | 0 | 0 |
| 9559 | PREDICTED: neuronal calcium sensor 2-like [Octopus bimaculoides] | Protein-Protein | 2 | 0 | 0 |
| 9631 | PREDICTED: mitochondrial-processing peptidase subunit beta-like [Octopus bimaculoides] | Metabolism | 1 | 0 | 0 |
| 9661 | PREDICTED: arylacetamide deacetylase-like isoform X1 [Octopus bimaculoides] | Metabolism | 0 | 0 | 1 |
| 9662 | PREDICTED: uncharacterized protein LOC106868256 isoform X2 [Octopus bimaculoides] | Uncharacterized | 1 | 0 | 0 |
| 9794 | PREDICTED: trifunctional enzyme subunit alpha, mitochondrial-like [Octopus bimaculoides] | Metabolism | 1 | 0 | 0 |
| 9944 | PREDICTED: acid ceramidase-like [Octopus bimaculoides] | Metabolism | 0 | 1 | 0 |
| 9948 | PREDICTED: F-actin-capping protein subunit alpha-2-like [Octopus bimaculoides] | Cytoskeleton | 0 | 1 | 0 |
| 9967 | PREDICTED: aspartyl/asparaginyl beta-hydroxylase-like [Biomphalaria glabrata] | Metabolism | 3 | 0 | 0 |
| 10120 | PREDICTED: protein-glutamine gamma-glutamyltransferase K-like [Octopus bimaculoides] | Protein-Protein | 1 | 0 | 0 |
| 10388 | PREDICTED: programmed cell death 6-interacting protein-like [Octopus bimaculoides] | Protein-Protein | 1 | 0 | 0 |
| 10410 | PREDICTED: ornithine carbamoyltransferase, mitochondrial-like isoform X1 [Octopus bimaculoides] | Metabolism | 1 | 0 | 0 |
| 10478 | PREDICTED: H(+)/Cl(-) exchange transporter 3-like isoform X2 [Octopus bimaculoides] | Ion Transport | 0 | 1 | 0 |
| 10634 | PREDICTED: eukaryotic translation initiation factor 3 subunit C-like [Octopus bimaculoides] | Metabolism | 1 | 0 | 0 |
| 10775 | PREDICTED: fatty acid-binding protein, liver-like [Octopus bimaculoides] | Metabolism | 3 | 0 | 0 |
| 10871 | PREDICTED: peptidyl-prolyl cis-trans isomerase B-like [Octopus bimaculoides] | Metabolism | 3 | 0 | 0 |
| 10874 | Arginase type I-like protein [Hyriopsis cumingii] | Metabolism | 0 | 2 | 0 |
| 10877 | PREDICTED: ribosome-binding protein 1-like [Octopus bimaculoides] | Membrane | 0 | 0 | 0 |
| 11116 | PREDICTED: MAM and LDL-receptor class A domain-containing protein 2-like [Octopus bimaculoides] | Membrane | 0 | 0 | 0 |
| 11557 | Poly endoribonuclease-c-like-specific [Mytilus galloprovincialis] | Metabolism | 0 | 0 | 2 |
| 11565 | Hypothetical protein OCBIM_22013360mg [Octopus bimaculoides] | Uncharacterized | 2 | 0 | 0 |
| 11686 | Thioredoxin reductase 3-like [Scleropages formosus] | Metabolism | 0 | 1 | 0 |
| 11728 | PREDICTED: integrin alpha-6-like [Octopus bimaculoides] | Membrane | 0 | 0 | 1 |
| 11843 | PREDICTED: probable methylmalonate-semialdehyde dehydrogenase [acylating], mitochondrial [Octopus bimaculoides] | Metabolism | 0 | 1 | 0 |
| 12130 | PREDICTED: neurocalcin homolog isoform X2 [Octopus bimaculoides] | Protein-Protein | 0 | 0 | 2 |
| 12340 | PREDICTED: histone deacetylase 11-like isoform X2 [Octopus bimaculoides] | Metabolism | 0 | 0 | 2 |
| 13086 | PREDICTED: fatty aldehyde dehydrogenase-like [Octopus bimaculoides] | Metabolism | 1 | 0 | 0 |
| 13337 | PREDICTED: carbonyl reductase [NADPH] 1-like [Octopus bimaculoides] | Metabolism | 0 | 1 | 0 |
| 13839 | PREDICTED: cytochrome c oxidase subunit 7A-related protein, mitochondrial-like [Octopus bimaculoides] | Metabolism | 0 | 3 | 0 |
| 13901 | PREDICTED: 40S ribosomal protein S15a-like [Octopus bimaculoides] | Metabolism | 3 | 0 | 0 |
| 14069 | Glucose-methanol-choline oxidoreductase [Sporothrix insectorum RCEF 264] | Metabolism | 3 | 0 | 0 |
| 14506 | PREDICTED: 40S ribosomal protein S15-like [Octopus bimaculoides] | Metabolism | 0 | 4 | 0 |
| 14666 | 40S ribosomal protein S3a [Orbicella faveolata] | Metabolism | 0 | 0 | 3 |
| 14743 | Hypothetical protein OCBIM_22013360mg [Octopus bimaculoides] | Uncharacterized | 3 | 0 | 0 |
| 14937 | PREDICTED: cytoplasmic phosphatidylinositol transfer protein 1-like [Octopus bimaculoides] | Metabolism | 3 | 0 | 0 |
| 15124 | PREDICTED: general transcription factor 3C polypeptide 1-like [Octopus bimaculoides] | Metabolism | 0 | 0 | 4 |
| 15246 | PREDICTED: maleylacetoacetate isomerase-like [Octopus bimaculoides] | Metabolism | 2 | 0 | 0 |
| 15589 | PREDICTED: iduronate 2-sulfatase-like [Octopus bimaculoides] | Metabolism | 2 | 0 | 0 |
| 16292 | PREDICTED: importin-5-like [Octopus bimaculoides] | Protein-Protein | 0 | 0 | 0 |
| 16601 | PREDICTED: thioredoxin-like [Octopus bimaculoides] | Metabolism | 4 | 0 | 0 |
| 16981 | PREDICTED: thioredoxin-related transmembrane protein 1-like [Octopus bimaculoides] | Metabolism | 0 | 4 | 0 |
| 17158 | PREDICTED: gelsolin-like protein 2 [Octopus bimaculoides] | Cytoskeleton | 1 | 0 | 0 |
| 17371 | RAD50-interacting protein 1 [Labrus bergylta] | Protein-Protein | 0 | 0 | 3 |
| 17450 | PREDICTED: cytochrome P450 20A1-like [Octopus bimaculoides] | Metabolism | 3 | 0 | 0 |
| 17477 | PREDICTED: uncharacterized protein LOC106884065 isoform X1 [Octopus bimaculoides] | Uncharacterized | 0 | 1 | 0 |
| 17739 | PREDICTED: histone H1-delta-like [Octopus bimaculoides] | Nucleosome | 3 | 0 | 0 |
| 17988 | Ribosomal protein L12, partial [Orbicella franksi] | Metabolism | 0 | 0 | 3 |
| 18713 | Hypothetical protein OCBIM_22031876mg [Octopus bimaculoides] | Uncharacterized | 4 | 0 | 0 |
| 18803 | PREDICTED: ubiquitin-conjugating enzyme E2 variant 2-like [Octopus bimaculoides] | Metabolism | 3 | 0 | 0 |
| 19325 | PREDICTED: 40S ribosomal protein S26 [Octopus bimaculoides] | Metabolism | 4 | 0 | 0 |
| 20227 | Glutaredoxin [Haliotis diversicolor supertexta] | Metabolism | 4 | 0 | 0 |
| 20390 | PREDICTED: general transcription factor 3C polypeptide 1-like [Octopus bimaculoides] | Metabolism | 5 | 0 | 0 |
| 20778 | PREDICTED: transmembrane protein 254-like isoform X2 [Octopus bimaculoides] | Membrane | 0 | 0 | 8 |
| 20854 | PREDICTED: histidine triad nucleotide-binding protein 3-like [Biomphalaria glabrata] | Metabolism | 5 | 0 | 0 |

**Table S2.** Proteins from isolated and purified granules (N = 1 MS/MS). Samples were not separated by color; instead they were collected from whole skin sections across the dorsal and ventral regions of four animals, pooled and distributed at random throughout the extraction and identification studies.

| Accession # | Protein Name | Category | Peptide Count |
| --- | --- | --- | --- |
| 11724 | Omega-crystallin [Octopus bimaculoides] | Crystallin | 158 |
| 21216 | Collagen alpha-4(VI) chain-like [Octopus bimaculoides] | Extracellular Matrix | 154 |
| 20135 | Carboxypeptidase A | Metabolism | 109 |
| 6812 | Immunoglobulin E-set | Metabolism | 103 |
| 5905 | Voltage-dependent anion-selective channel protein 2-like [Octopus bimaculoides] | Ion Transport | 98 |
| 11795 | Uncharacterized | Uncharacterized | 98 |
| 3200 | ADP,ATP carrier protein 3, mitochondrial-like [Octopus bimaculoides] | Membrane | 71 |
| 11325 | CD63 antigen-like [Aplysia californica] | Membrane | 52 |
| 6946 | Glutathione S-transferase Y1-like [Crassostrea gigas] | Metabolism | 47 |
| 16456 | PREDICTED: hemocyte protein-glutamine gamma-glutamyltransferase-like isoform X2 [Octopus bimaculoides] | Protein-protein | 46 |
| 5134 | Phosphate carrier protein, mitochondrial-like [Octopus bimaculoides] | Membrane | 44 |
| 6813 | Vacuolar protein sorting-associated protein 4B | Metabolism | 37 |
| 17802 | Tubulin beta-4B chain | Cytoskeleton | 37 |
| 5469 | Matrilin 2 | Extracellular Matrix | 35 |
| 6955 | Elongation factor 1-alpha | Metabolism | 35 |
| 5143 | Translocon-associated protein subunit beta | Metabolism | 29 |
| 16139 | PREDICTED: ATP synthase subunit beta, mitochondrial-like [Octopus bimaculoides] | Metabolism | 28 |
| 1684 | PREDICTED: pyridoxal 5'-phosphate synthase subunit SNZERR-like [Octopus bimaculoides] | Metabolism | 26 |
| 9250 | Tubulin α-1B | Cytoskeleton | 24 |
| 16741 | PREDICTED: casein kinase II subunit alpha isoform X2 [Aplysia californica] | Metabolism | 21 |
| 5009 | Uncharacterized Prot | Metabolism | 21 |
| 10106 | Vitellinogen, open beta-sheet | Metabolism | 20 |
| 10636 | Ribonuclease T2 | Metabolism | 20 |
| 13974 | Histone H2B | Nucleosome | 19 |
| 13200 | Annexin | Protein-protein | 17 |
| 8375 | PREDICTED: collagen alpha-1(XII) chain-like [Octopus bimaculoides] | Extracellular Matrix | 16 |
| 9551 | Belongs to the 3-beta-HSD family | Metabolism | 16 |
| 8572 | Uncharacterized | Uncharacterized | 16 |
| 8660 | Calcium ion binding | Metabolism | 16 |
| 11219 | CBN-EMB-9 protein | Protein-protein | 14 |
| 9330 | EGF-like domain | Membrane | 14 |
| 17780 | PREDICTED: integrin beta-2-like [Octopus bimaculoides] | Membrane | 14 |
| 7836 | Paramyosin | Cytoskeleton | 13 |
| 20952 | ATP synthase subunit alpha | Metabolism | 13 |
| 11343 | Ferritin | Metabolism | 13 |
| 2093 | Elongation factor 1-alpha | Metabolism | 12 |
| 13238 | PREDICTED: zonadhesin-like [Octopus bimaculoides] | Protein-protein | 12 |
| 6997 | Transferrin-like [Octopus bimaculoides] | Membrane | 12 |
| 13241 | Collagen alpha-4(VI) chain-like [Lingula anatina] | Extracellular Matrix | 12 |
| 9065 | MACPF domain containing protein | Membrane | 12 |
| 5650 | PREDICTED: prohibitin-like [Octopus bimaculoides] | Metabolism | 10 |
| 9781 | Collagen alpha-2(IV) chain-like [Octopus bimaculoides] | Extracellular Matrix | 10 |
| 8515 | Isocitrate dehydrogenase [NADP] | Metabolism | 10 |
| 1245 | PREDICTED: 60S ribosomal protein L23a-like [Octopus bimaculoides] | Metabolism | 9 |
| 8780 | Uncharacterized | Uncharacterized | 8 |
| 13237 | PREDICTED: laminin subunit alpha-like isoform X2 [Lingula anatina] | Extracellular Matrix | 8 |
| 8042 | Malate dehydrogenase | Metabolism | 7 |
| 10121 | PREDICTED: copine-3-like [Octopus bimaculoides] | Membrane | 7 |
| 9091 | PREDICTED: zinc metalloproteinase nas-15-like [Aplysia californica] | Metabolism | 6 |
| 9620 | PREDICTED: laminin subunit beta-1-like [Octopus bimaculoides] | Extracellular Matrix | 6 |
| 11739 | Complement component C3-like protein [Euprymna scolopes] | Protein-protein | 6 |
| 9821 | Prohibitin 2 [Sepiella japonica] | Metabolism | 6 |
| 11898 | PREDICTED: protein DD3-3-like [Lingula anatina] | Metabolism | 6 |
| 13274 | Laminin subunit gamma-1 | Extracellular Matrix | 5 |
| 6049 | PREDICTED: cathepsin L1-like [Octopus bimaculoides] | Metabolism | 5 |
| 10075 | Uncharacterized Prot | Membrane | 5 |
| 11735 | Complement component C3-like protein [Euprymna scolopes] | Protein-protein | 5 |
| 9086 | Spectrin beta chain-like [Octopus bimaculoides] | Cytoskeleton | 5 |
| 13189 | Basement membrane-specific heparan sulfate proteoglycan core protein | Extracellular Matrix | 4 |
| 7854 | PREDICTED: cysteine-rich secretory protein 3-like [Octopus bimaculoides] | Protein-protein | 4 |
| 6681 | EGF-like domain | Membrane | 4 |
| 8129 | PREDICTED: protein disulfide-isomerase 2-like [Octopus bimaculoides] | Metabolism | 4 |
| 11240 | PREDICTED: inter-alpha-trypsin inhibitor heavy chain H5-like [Octopus bimaculoides] | Metabolism | 3 |
| 10357 | Apolipoprotein B | Protein-protein | 3 |
| 10585 | PREDICTED: neurotrypsin-like [Limulus polyphemus] | Metabolism | 3 |
| 12393 | Basement membrane-specific heparan sulfate proteoglycan core protein | Extracellular Matrix | 3 |
| 12352 | PREDICTED: beta-mannosidase-like [Octopus bimaculoides] | Metabolism | 3 |
| P85084 | Endochitinase | Contaminant | 233 |
| P00784 | Papain | Contaminant | 134 |
| P35059 | Histone H4 | Nucleosome | 127 |
| P05994 | Papaya proteinase 4 | Contaminant | 60 |
| P09870 | Clostripain | Contaminant | 19 |

**Table S3.** Proteins in extracted granules; (N = 1 MS/MS). Samples were not separated by color; instead they were collected from whole skin sections across the dorsal and ventral regions of four animals, pooled and distributed at random throughout the extraction and identification studies.

| Accession # | Protein Name | Category | Peptide Count |
| --- | --- | --- | --- |
| 11724 | Omega-crystallin [Octopus bimaculoides] | Crystallin | 32 |
| 5905 | Voltage-dependent anion-selective channel protein 2-like [Octopus bimaculoides] | Ion Transport | 25 |
| 21216 | Collagen alpha-4(VI) chain-like [Octopus bimaculoides] | Extracellular Matrix | 18 |
| 6946 | Glutathione S-transferase Y1-like [Crassostrea gigas] | Metabolism | 8 |
| 6955 | Elongation factor 1-alpha | Metabolism | 7 |
| 5469 | Matrilin 2 | Extracellular Matrix | 6 |
| 6812 | Immunoglobulin E-set | Metabolism | 5 |
| 11240 | PREDICTED: inter-alpha-trypsin inhibitor heavy chain H5-like [Octopus bimaculoides] | Metabolism | 4 |
| 6680 | PREDICTED: collagen alpha-4(VI) chain-like [Octopus bimaculoides] | Extracellular Matrix | 2 |
| 9330 | EGF-like domain | Membrane | 2 |
| 13189 | Basement membrane-specific heparan sulfate proteoglycan core protein | Extracellular Matrix | 2 |
| P85084 | Endochitinase | Contaminant | 70 |
| P00784 | Papain | Contaminant | 38 |
| P05994 | Papaya proteinase 4 | Contaminant | 27 |
| C3K2Y6 | 50S ribosomal protein L1 | Contaminant | 20 |
| C3K2X8 | Elongation factor Tu | Contaminant | 11 |
| P00761 | Trypsin | Contaminant | 30 |
| Q3K603 | 50S ribosomal protein L6 | Contaminant | 22 |
| Q3K5Y8 | 50S ribosomal protein L3 | Contaminant | 17 |
| Q3KFU6 | Succinyl-CoA ligase [ADP-forming] subunit beta | Contaminant | 8 |
| Q4K556 | 30S ribosomal protein S4 | Contaminant | 11 |

**Table S4.** Proteins Found in Pigment; (N = 1 MS/MS). Samples were not separated by color; instead they were collected from whole skin sections across the dorsal and ventral regions of four animals, pooled and distributed at random throughout the extraction and identification studies.

| Accession # | Protein Name | Category | Peptide Count |
| --- | --- | --- | --- |
| 5905 | Voltage-dependent anion-selective channel protein 2-like [Octopus bimaculoides] | Ion Transport | 31 |
| 11724 | Omega-crystallin [Octopus bimaculoides] | Crystallin | 22 |
| 21216 | Collagen alpha-4(VI) chain-like [Octopus bimaculoides] | Extracellular Matrix | 16 |
| 5469 | Matrilin 2 | Extracellular Matrix | 8 |
| 11240 | PREDICTED: Inter-alpha-trypsin inhibitor heavy chain H5-like [Octopus bimaculoides] | Metabolism | 7 |
| 9330 | EGF-like domain | Membrane | 2 |
| 10106 | Vitellogenin | Metabolism | 1 |
| P35059 | Histone H4 | Nucleosome | 103 |
| P85084 | Endochitinase | Contaminant | 79 |
| P00784 | Papain | Contaminant | 35 |
| P05994 | Papaya proteinase 4 | Contaminant | 19 |
| P14080 | Chymopapain | Contaminant | 12 |
| P02769 | Serum albumin | Contaminant | 4 |

**Table S5.** Statistics of the top ten threading templates used by I-TASSER for the structural prediction of Ω-crystallin. In I-TASSER terminology, *Iden1* is the percentage sequence identity of the templates in the threading aligned region with the query sequence. *Iden2* is the percentage sequence identity of the whole template chains with query sequence. *Cov* represents the coverage of the threading alignment and is equal to the number of aligned residues divided by the length of query protein. *Norm. Z-score* is the normalized Z-score of the threading alignments. Alignment with a Normalized Z-score >1 mean a good alignment and vice versa.


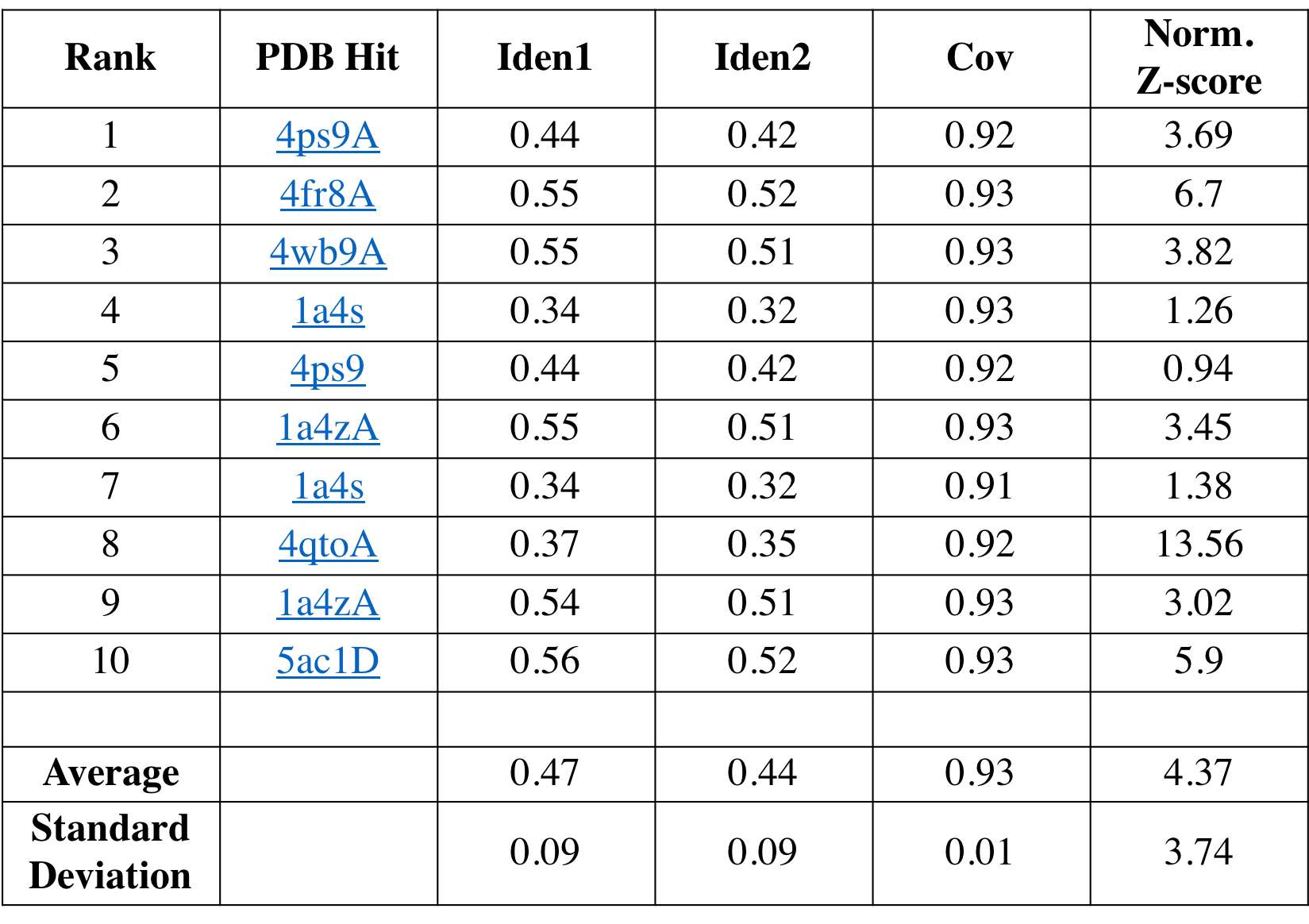


**Table S6.** Statistics of the top ten structural analogs identified by I-TASSER for the predicted structure of Ω-crystallin. In I-TASSER terminology, *TM-score* is a metric that has values between (0,1) for measuring the structural similarity of two protein models. A value of 1 indicates a perfect match between two structures, scores below 0.17 corresponds to randomly chosen unrelated proteins whereas with a score higher than 0.5 assumes generally the same fold in SCOP/CATH. *RMSD^a^* is the RMSD between residues that are structurally aligned by TM-align. *IDEN^a^* is the percentage sequence identity in the structurally aligned region. *Cov* represents the coverage of the alignment by TM-align and is equal to the number of structurally aligned residues divided by length of the query protein.


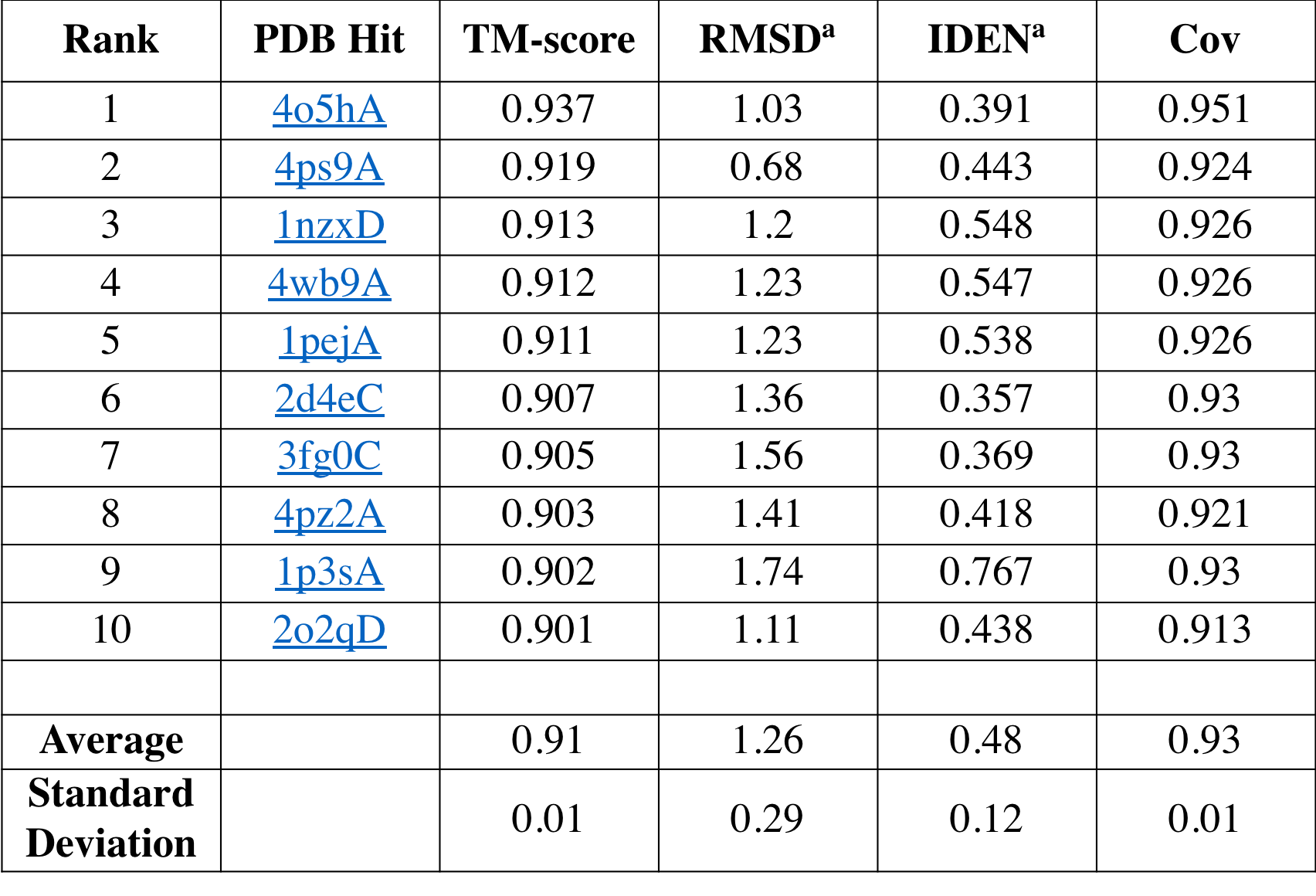


**Table S7.** Statistics of the top ten threading templates used by I-TASSER for the structural prediction of reflectin.


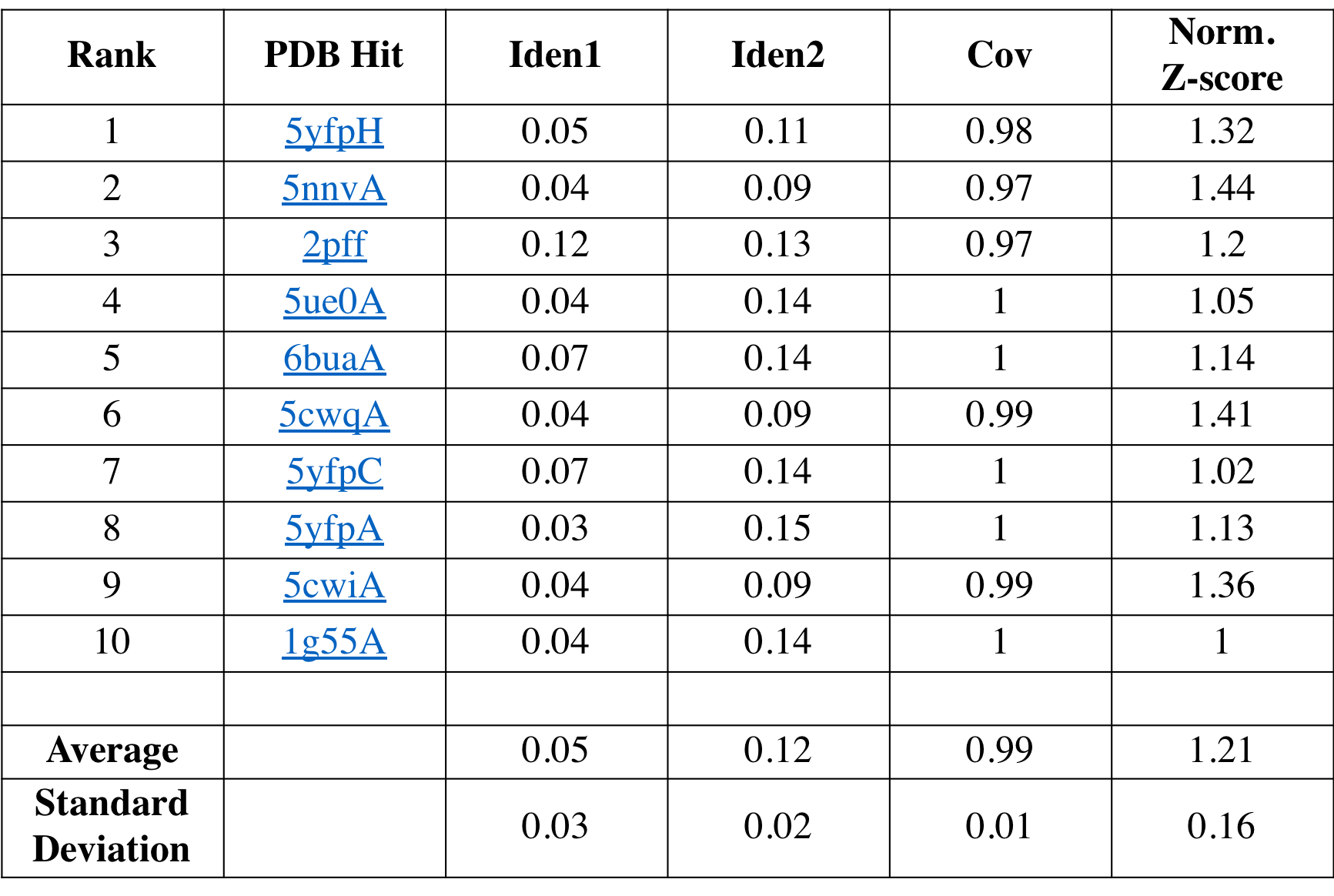


**Table S8.** Statistics of the top ten structural analogs identified by I-TASSER for the predicted structure of reflectin.


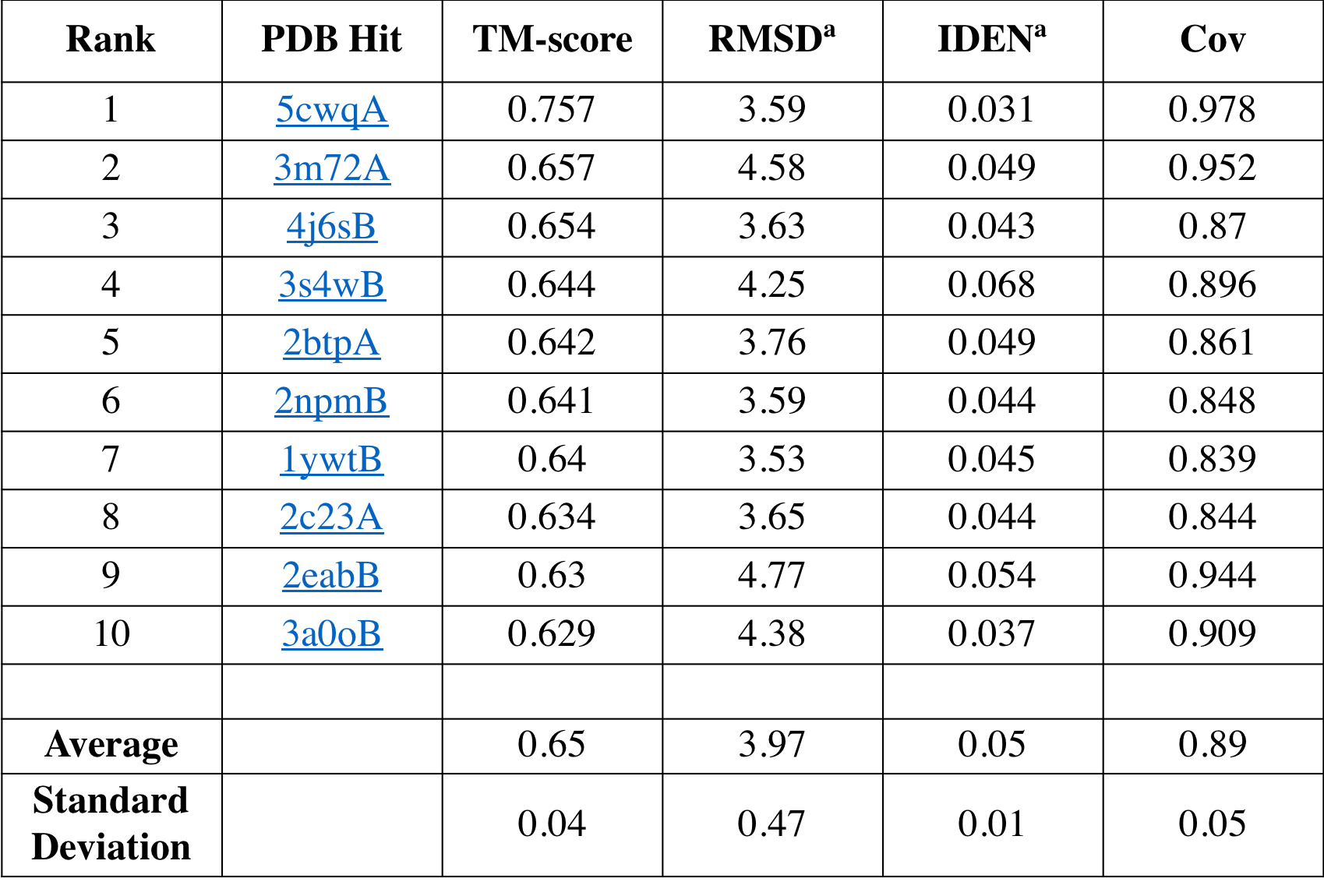


**Table S9.** Comparison of the most favourable binding energies (**Supplementary Notes S3**) after docking of small, aromatic molecules to both monomeric crystallin and reflectin.

| Most Favourable Binding Energies (kcal mol^-1^) | | | |
| --- | --- | --- | --- |
|  | Crystallin | Reflectin | Difference |
| Xanthommatin | -10.9 | -9.5 | -1.4 |
| Kynurenine | -7.5 | -7.0 | -0.5 |
| Xanthopterin | -7.9 | -6.3 | -1.6 |
| Xanthurenic Acid | -8.2 | -7.0 | -1.2 |
| Tryptophan | -8.8 | -7.3 | -1.5 |
| Cyanidin | -9.6 | -8.8 | -0.8 |
| Phenoxazine | -8.5 | -7.4 | -1.1 |

**Supplementary Notes**

**S1. Significance Analysis of Spectral Count Data**

Spectral count data for Figure S2 was processed using QSpec^4^. Because QSpec compares samples pairwise, chromatophore types were placed into pairs: brown and red, brown and yellow, and red and yellow. Each set of data was processed by the QSpec software, which also normalized each set by protein content and scaled individual proteins by molecular weight. For each protein in each pair of chromatophore types, this provides both the fold change, a measure of the change in protein abundance, as well as the calculated false discovery rate (FDR). For each detected protein, the -log_10_(FDR) was plotted against the log_2_(Fold Change) and colored by assigned category.

**S2. Details of Immunocytochemistry**

Fixative containing 4% paraformaldehyde in Hepes buffer (in filtered NSW) was poured over the skin, quickly expanding the chromatophores. The tissue was placed on a shaker overnight at 10°C. It was then transferred into 1x PBS containing 50 mM glycine (to remove unbound aldehyde) and the fixed iridophore and hyalin layers were manually peeled off from the lower and upper surface of the chromatophore layer (for better chemical penetration and microscopic observation). The tissues were cut into smaller pieces (roughly 1/2 inch or less) further rinsed in PBS, then placed overnight in blocking solution (PBS containing 2% goat serum, 0.25% triton-X, and occasionally 0.1% BSA). Primary (rabbit) antibody (kindly provided by Dr. Daniel Morse [anti-A1/A2] and by Dr. Wendy Goodson) was diluted (~1:200-300, 50 ul to 15 ml) in blocking solution. Then 0.5 to 1.0 ml was added to each well (except control) of a 24-well plastic Costar culture cluster, containing tissue pieces. This was shaken for 12 or 36 hours, then washed (3 times 30 min, with shaking) in 1.0 ml PBS. Secondary (goat-anti-rabbit) antibody was diluted (~1:250) in PBS and added to the tissue (transferred to a fresh plastic Costar) and again shaken overnight. A single (Alexa488 or Alexa568) anti-rabbit secondary was used for some experiments; two anti-rabbit secondary antibodies (Alexa405 and Alexa568) were mixed together in others. After a final PBS wash (3 times 30 min, with shaking), the sections were mounted in Prolong Gold (with DAPI) between two coverslips so that they might be viewed from either side using short working distance lenses. Mounted material was imaged in 2D and 3D using a Zeiss 780 confocal microscope using 10x 0.45NA, 20x 0.80NA, and 40x 1.4NA lenses (and electronic zoom from 0.7 to 3.0x), set to simultaneously image DAPI, Alexa488, and Alexa568, at 1K and 2K pixel dimensions, and saved as composite RGB data in the Zeiss “czi” format. SBFI (serial block-face imaging) data was obtained with the assistance of Gatan, Inc. and FEI, Inc., using tissue that we fixed in 4% parafomaldehyde and 2.5% glutaraldehyde, and stained with heavy metals based on the protocol of Deerinck and coworkers^5^.

**S3. Computational Structure Prediction for Xanthommatin, Crystallin, and Reflectin**

For the docking experiments, we obtained the structure for the pigment molecule (Xa) after geometry optimizations from different initial structures with Density Functional Theory (DFT) using the Gaussian 09 computational package^6^. The global hybrid functional M062x^6^ with Hartree Fock exchange were used, together with 6-31G* basis set^8^. Implicit solvent effects were included with the Polarizable Continuum Model (PCM) using the integral equation formalism variant (IEFPCM)^9^, which creates the solute cavity via a set of overlapping spheres. Homologous protein structures were obtained from the I-TASSER homology modeling webserver^10^ using the identified protein sequence of Ω-crystallin and reflectin. The top-ranked models that have the largest cluster sizes was further refined using replica exchange molecular dynamics (MD) simulations with solute tempering (REST2)^11^. This was implemented in the NAMD 2.10 open-source MD software^12,13^ in conjunction with the CHARMM36m forcefield^14^. MD simulations with REST2 greatly reduces the computational costs required by regular replica exchange and increases the efficiency of sampling protein conformations. The use of REST2 has, for instance, been successfully implemented in other manuscripts to predict *ab initio* folding of the trpcage protein^8^ and silkworm silk aggregation in different solvents^15^. Water molecules were modeled explicitly and sufficient counterions were added to create a neutral charge environment. 16 replicas were defined with temperatures ranging between 300 to 400 K and simulations were performed for 10 ns per replica for a total sampling time of 160 ns. The time step was 2 fs, conformations of the proteins were saved every 20 ps, and exchange of configurations between neighboring replicas were attempted every 2 ps with an average acceptance ratio of more than 20%, indicating adequate sampling. The trajectory over the last 5 ns was clustered with the GROMOS method^16^ and a cut-off of 0.3nm, using analysis tools from the GROMACS MD software^17^. The structure with the largest cluster size was used in the docking studies. The tetrameric structure was constructed in Chimera^18^ through structural alignment of four Ω-crystallin monomers with the top-ranked protein in terms of structural similarity (PDB: 4O5H) as predicted by I-TASSER. The normalized Z-scores calculated for the predicted reflectin structure were all greater than 1 with an average value of 1.21 ± 0.16 (N= 10 and error is standard deviation, **Table S7**), indicating a good alignment of the predicted tertiary structure of reflectin with the templates generated by I-TASSER. Additionally, the calculated template modelling scores (TM-scores) were consistently greater than 0.5 with an average of 0.65 ± 0.04 (N= 10 and error is standard deviation, **Table S8**), indicating good similarities in the predicted models and the structural analogues of reflectin predicted from I-TASSER (listed in **Table S8**).

**S4. Electrostatic Potential Maps of Crystallin**

The electrostatic potential map of the crystallin monomer and tetramer were calculated with the Adaptive Poisson-Boltzmann Solver (APBS)^19^ implemented in Chimera. Atomic charges were assigned according to the CHARMM forcefield with PDB2PQR^20,21^.

**S5. Ligand-Protein Docking**

Ligand-protein docking was performed in Autodock Vina^22^ through the PyMol interface^23^. To locate potential binding pockets for each protein monomer, twenty docked poses per monomer were generated from rigid docking using a grid size that encompassed the globular portions of each monomer and a grid spacing of 0.375 Å. The top five poses, ranked according to their binding energies, belonged to only two pockets for both crystallin and reflectin. Therefore, the most energetically favorable poses in each pocket were used for further refinement with flexible docking to determine the final docked conformations. The grid size was 22.5 × 22.5 × 22.5 Å^3^ with a grid spacing of 0.375 Å, centered at the ligands’ center-of-mass of the respective poses. Sidechains of the monomers that had atoms within a distance of 3.5 Å from the ligand were defined to be flexible.

**S6. Visualization**

All figures derived from computational data were visualized using the VMD software^24^. Maps of the interacting ligands and amino acid sidechains were plotted with the LigPlot+ software^25^. Ligands and sidechains were defined to be in contact if they were within a distance of 3.9 Å. Polar interactions were defined to be acceptor-donor pairs that were within a distance of 3.35 Å.

**S7. Absorption spectra calculations**

The molecular structures of Xa were optimized with DFT using ORCA quantum chemistry package^26^. The dispersion correction DFT-D3 with Becke-Johnson damping (D3BJ)^27^ was implemented to describe non-covalent intramolecular interactions. The SMD model^28^, a continuum solvation model based on the quantum mechanical charge density of a solute molecule interacting with a continuum description of the solvent, was adopted to take water effects into account. The B3LYP^29,30^ functional was adopted together with the 6-31G(d) basis set^31^ for all geometry optimizations. The recently developed geometrical counterpoise correction (gCP)^32^ was adopted to circumvent the so-called basis set superposition error (BSSE) when applying small basis sets^33,34^.

After the molecular structures of Xa were optimized, their absorption spectra were calculated with Time-Dependent Density Functional Theory (TDDFT). As with the method adopted in the geometry optimizations mentioned above, the dispersion correction D3BJ, SMD model for water, and geometrical counterpoise correction (gCP) were also implemented for consistency. Instead of using the B3LYP functional, here we adopted the BLYP^35,36^ functional together with the 6-31G(d) basis set since the pure BLYP functional has been shown to reproduce absorption spectra better than the B3LYP hybrid functional^37^.

**Supplementary References**

1 Kinoshita, S., Yoshioka, S. & Miyazaki, J. Physics of structural colors. *Rep. Prog. Phys.* **71** (2008).

2 Ghoshal, A., DeMartini, D. G., Eck, E. & Morse, D. E. Experimental determination of refractive index of condensed reflectin in squid iridocytes. *J. Roy. Soc. Int.* **11**, 20140106 (2014).

3 Ross, K. F. A. Measurement of the refractive index of cytoplasmic inclusions in living cells by the interference microscope. *Nature* **174**, 836 (1954).

4 Choi, H., Fermin, D. & Nesvizhskii, A. I. Significance analysis of spectral count data in label-free shotgun proteomics. *Mol. Cell. Proteomics* **7**, 2373-2385 (2008).

5 Deerinck, T. J. *et al.* Enhancing serial block-face scanning electron microscopy to enable high resolution 3-D nanohistology of cells and tissues. *Microsc. Microanal.* **16**, 1138-1139 (2010).

6 Frisch, M.J. *et al.* Gaussian 09, revision D. 01 (Gaussian, Inc., Wallingford CT, 2009).

7 Zhao, Y. & Truhlar, D. G. The M06 suite of density functionals for main group thermochemistry, thermochemical kinetics, noncovalent interactions, excited states, and transition elements: two new functionals and systematic testing of four M06-class functionals and 12 other functionals. *Theor. Chem. Acc.* **120**, 215-241 (2008).

8 Rassolov, V. A., Ratner, M. A., Pople, J. A., Redfern, P. C. & Curtiss, L. A. 6‐31G* basis set for third‐row atoms. *J. Comput. Chem.* **22**, 976-984 (2001).

9 Tomasi, J., Mennucci, B. & Cammi, R. Quantum mechanical continuum solvation models. *Chem. Rev.* **105**, 2999-3094 (2005).

10 Yang, J. *et al.* The I-TASSER Suite: protein structure and function prediction. *Nat. Methods* **12**, 7 (2014).

11 Wang, L., Friesner, R. A. & Berne, B. J. Replica exchange with solute scaling: a more efficient version of replica exchange with solute tempering (REST2). *J. Phys. Chem. B* **115**, 9431-9438 (2011).

12 Phillips, J. C. *et al.* Scalable molecular dynamics with NAMD. *J. Comput. Chem.* **26**, 1781-1802 (2005).

13 Jo, S. & Jiang, W. A generic implementation of replica exchange with solute tempering (REST2) algorithm in NAMD for complex biophysical simulations. *Comput. Phys. Commun.* **197**, 304-311 (2015).

14 Huang, J. *et al.* CHARMM36m: an improved force field for folded and intrinsically disordered proteins. *Nat. Methods* **14**, 71-73 (2016).

15 Zhu, Z. *et al.* High-strength, durable all-silk fibroin hydrogels with versatile processability toward multifunctional applications. *Adv. Funct. Mater.* **28**, 1704757 (2018).

16 Daura, X. *et al.* Peptide folding: when simulation meets experiment. *Angew. Chem. Int. Ed.* **38**, 236-240 (1999).

17 Abraham, M. J. *et al.* GROMACS: high performance molecular simulations through multi-level parallelism from laptops to supercomputers. *SoftwareX* **1–2**, 19-25 (2015).

18 Pettersen, E. F. *et al.* UCSF Chimera—A visualization system for exploratory research and analysis. *J. Comput. Chem.* **25**, 1605-1612 (2004).

19 Baker, N. A., Sept, D., Joseph, S., Holst, M. J. & McCammon, J. A. Electrostatics of nanosystems: application to microtubules and the ribosome. *Proc. Natl. Acad. Sci. U.S.A* **98**, 10037-10041 (2001).

20 Dolinsky, T. J. *et al.* PDB2PQR: expanding and upgrading automated preparation of biomolecular structures for molecular simulations. *Nucleic Acids Res.* **35**, W522-W525 (2007).

21 Dolinsky, T. J., Nielsen, J. E., McCammon, J. A. & Baker, N. A. PDB2PQR: an automated pipeline for the setup of Poisson–Boltzmann electrostatics calculations. *Nucleic Acids Res.* **32**, W665-W667 (2004).

22 Trott, O. & Olson, A. J. AutoDock Vina: improving the speed and accuracy of docking with a new scoring function, efficient optimization, and multithreading. *J. Comput. Chem.* **31**, 455-461 (2010).

23 The PyMOL Molecular Graphics System, Version 1.8 Schrödinger, LLC.

24 Humphrey, W., Dalke, A. & Schulten, K. VMD: visual molecular dynamics. *J. Mol. Graph.* **14**, 33-38, 27-38 (1996).

25 Laskowski, R. A. & Swindells, M. B. LigPlot+: multiple ligand-protein interaction diagrams for drug discovery. *J. Chem. Inf. Model* **51**, 2778-2786 (2011).

26 Neese, F. The ORCA program system. *Wiley Interdiscip. Rev. Comput. Mol. Sci.* **2**, 73-78 (2012).

27 Grimme, S., Ehrlich, S. & Goerigk, L. Effect of the damping function in dispersion corrected density functional theory. *J. Comput. Chem.* **32**, 1456-1465 (2011).

28 Marenich, A. V., Cramer, C. J. & Truhlar, D. G. Universal solvation model based on solute electron density and on a continuum model of the solvent defined by the bulk dielectric constant and atomic surface tensions. *J. Phys. Chem. B* **113**, 6378-6396 (2009).

29 Becke, A. D. A new mixing of Hartree–Fock and local density‐functional theories. *J. Chem. Phys.* **98**, 1372-1377 (1993).

30 Becke, A. D. Density‐functional thermochemistry. III. The role of exact exchange. *J. Chem. Phys.* **98**, 5648-5652 (1993).

31 Hehre, W. J., Ditchfield, R. & Pople, J. A. Self—consistent molecular orbital methods. XII. Further extensions of gaussian—type basis sets for use in molecular orbital studies of organic molecules. *J. Chem. Phys.* **56**, 2257-2261 (1972).

32 Kruse, H. & Grimme, S. A geometrical correction for the inter-and intra-molecular basis set superposition error in Hartree-Fock and density functional theory calculations for large systems. *J. Chem. Phys.* **136**, 154101 (2012).

33 Kruse, H., Goerigk, L. & Grimme, S. Why the standard B3LYP/6-31G* model chemistry should not be used in DFT calculations of molecular thermochemistry: understanding and correcting the problem. *J. Org. Chem.* **77**, 10824-10834 (2012).

34 Chen, C.-T., Martin-Martinez, F. J., Jung, G. S. & Buehler, M. J. Polydopamine and eumelanin molecular structures investigated with *ab initio* calculations. *Chem. Sci.* (2017).

35 Lee, C., Yang, W. & Parr, R. G. Development of the Colle-Salvetti correlation-energy formula into a functional of the electron density. *Phys. Rev. B* **37**, 785 (1988).

36 Becke, A. D. Density-functional exchange-energy approximation with correct asymptotic behavior. *Phys. Rev. A* **38**, 3098 (1988).

37 Li, W. *et al.* DFT/TDDFT studies of the geometry, electronic structure and spectra of (12S)-1, 4, 7, 10-tetraazadicyclo [10, 3, 0]-pentadecane-3, 11-dione and its derivatives. *J. Phys. Chem. A* **109**, 2878-2886 (2005).
