# Supplementary material for: Dynamic pigmentary and structural coloration within cephalopod chromatophore organs
Source: Nat Commun. 2019 Mar 1;10:1004. doi: 10.1038/s41467-019-08891-x (PMC6397165; doi:10.1038/s41467-019-08891-x)
Supplement: Supplementary file 2 — Description of Additional Supplementary Files [file 41467_2019_8891_MOESM2_ESM.docx]

**Title: Supplementary Movie 1
Description:** Anterior mantle of a living unanaesthetized squid, at 60 fps, showing motion from respiration. Mantle is tiled with “discoid units” (one central brown chromatophore encircled by about a dozen smaller brown and red chromatophores that have several dozen yellow chromatophores ringed between them). A hand-held light pipe (and ambient daylight) provides the illumination. Linear patches of bright yellow can be seen reflecting off of the surface of the open chromatophores of all three color types. Different sets of chromatophores sparkle as the angle of the incident light changes. The large patches of whitish-green are iridophores (clusters of hundreds of small iridocytes, each considerably smaller than a yellow chromatophore, and at this magnification producing a more diffuse reflectance).

**Title: Supplementary Movie 2
Description:** Excised patch of adult squid skin, still attached to the white underlying mantle muscle. The outermost (hyaline) layer of the skin has been removed. Illumination by a stabilized light pipe (ca. 6 mm diameter) is swept by hand through a small angle (estimated to be 10-20 degrees) during the course of the clip. All three colors of chromatophore show strong multi-hued reflectance. Yellow chromatophores show the widest range of iridescent hues. The regions of reflection coincide precisely with the moving dimensions (and apparently even with the fine topographic texture) of the saccule. 30 fps. Scale: yellow chromatophores are approximately 450 µm in diameter when fully open.

**Title: Supplementary Movie 3
Description:** A field of highly reflective yellow chromatophores that lied just above less-reflective browns. White mantle muscle below. Angle of incident light pipe illumination changes over time and becomes sufficiently different that the regional “saran-wrap-like” reflection of the yellow chromatophores comes and goes (at times revealing the shape of the chromatophore through absorbance alone). In this video, hue tends to stay in a narrow range for any given portion of each moving chromatophore, even though the angle of the light changes (by an uncalibrated but significant amount). 30 fps. Scale: yellow chromatophores are approximately 450 µm in diameter when fully open.

**Title: Supplementary Movie 4
Description:** A single expanded yellow chromatophore illuminated by a hand-held fiber optic light pipe. Many colors are reflected, showing hue adjacency much like Newton’s series. In each region, the hues change as the chromatophore deforms slightly, but they do not seem to change systematically as the angle of the light is gradually moved back and forth during the movie segment. It is possible that these hues result from thin-film interference between components of the chromatophore organ which maintain a close adjacency that varies across the expanded organ. The brightness of the colors is reminiscent of the second and third order series of the Michel-Lévy interference color chart. A simple inference could be that there are reflecting interfaces (of differing refractive index) separated by some portion of a micron to several microns, and that this separation (and or refractive index difference) is greater towards the center of the chromatophore. Potentially this could be due to interactions among sheath cell membranes, or between sheath cells and the chromatocyte saccule (or even the surface of the mass of chromatophore granules when pulled into a flat disc).

**Title: Supplementary Movie 5
Description:** Squid skin after ethanol anaesthesia and decapitation. Skin is being stimulated electrically in four epochs with a Grass S9 stimulator, with one lead touching the skin and another in the bath. The illumination is stationary. The stimulation leads to transient chromatophore opening, and the light is angled such that the yellow chromatophores briefly show regions of strong reflectivity. The induction of this skin reflection is very rapid. (In contrast, stimulation of iridophores (none are visible in this field of view] produces iridescence that is known to have a rise and fall time of many seconds to minutes). 30 fps. Scale: yellow chromatophores are approximately 450 µm in diameter when fully open.
