# Supplementary material for: Dynamic pigmentary and structural coloration within cephalopod chromatophore organs
Source: Nat Commun. 2019 Mar 1;10:1004. doi: 10.1038/s41467-019-08891-x (PMC6397165; doi:10.1038/s41467-019-08891-x)
Supplement: Supplementary file 8 — Reporting Summary [file 41467_2019_8891_MOESM8_ESM.pdf]

nature research

Corresponding author(s): Deravi and Hanlon

Reporting Summary

Nature Research wishes to improve the reproducibility of the work that we publish. This form provides structure for consistency and transparency in reporting. For further information on Nature Research policies, see Authors & Referees and the Editorial Policy Checklist.

Statistical parameters

When statistical analyses are reported, confirm that the following items are present in the relevant location (e.g. figure legend, table legend, main text, or Methods section).

n/a	Confirmed	
☐	☒	The exact sample size (n) for each experimental group/condition, given as a discrete number and unit of measurement
☐	☒	An indication of whether measurements were taken from distinct samples or whether the same sample was measured repeatedly
☒	☐	The statistical test(s) used AND whether they are one- or two-sided<br>*Only common tests should be described solely by name; describe more complex techniques in the Methods section.*
☒	☐	A description of all covariates tested
☐	☒	A description of any assumptions or corrections, such as tests of normality and adjustment for multiple comparisons
☒	☐	A full description of the statistics including central tendency (e.g. means) or other basic estimates (e.g. regression coefficient) AND variation (e.g. standard deviation) or associated estimates of uncertainty (e.g. confidence intervals)
☒	☐	For null hypothesis testing, the test statistic (e.g. F, t, r) with confidence intervals, effect sizes, degrees of freedom and P value noted<br>*Give P values as exact values whenever suitable.*
☒	☐	For Bayesian analysis, information on the choice of priors and Markov chain Monte Carlo settings
☒	☐	For hierarchical and complex designs, identification of the appropriate level for tests and full reporting of outcomes
☒	☐	Estimates of effect sizes (e.g. Cohen's d, Pearson's r), indicating how they were calculated
☒	☐	Clearly defined error bars<br>*State explicitly what error bars represent (e.g. SD, SE, CI)*

Our web collection on statistics for biologists may be useful.

Software and code

Policy information about availability of computer code

Data collection	We used ImageJ to compile immunocytochemical images.
Data analysis	We used originPro, SigmaPlot, and microsoft Excel to analyze and plot data

For manuscripts utilizing custom algorithms or software that are central to the research but not yet described in published literature, software must be made available to editors/reviewers upon request. We strongly encourage code deposition in a community repository (e.g. GitHub). See the Nature Research guidelines for submitting code & software for further information.

Data

Policy information about availability of data

All manuscripts must include a data availability statement. This statement should provide the following information, where applicable:

- Accession codes, unique identifiers, or web links for publicly available datasets
- A list of figures that have associated raw data
- A description of any restrictions on data availability

All data generated or analysed during this study are included in this published article (and its supplementary information files).

Field-specific reporting

Please select the best fit for your research. If you are not sure, read the appropriate sections before making your selection.

☒ Life sciences ☐ Behavioural & social sciences ☐ Ecological, evolutionary & environmental sciences

For a reference copy of the document with all sections, see nature.com/authors/policies/ReportingSummary-flat.pdf

Life sciences study design

All studies must disclose on these points even when the disclosure is negative.

Sample size	Where appropriate, spectral data collected from samples were measured in triplicate from at least two technical replicates. Data shown are either representative of each condition or otherwise stated in the caption. For mass spectrometric analysis, peptide counts were normalized by predicted peptide count and by total peptide abundance per chromatophore type. (n=1 MS/MS for each color, which included a total of ~700 yellow, ~700 red, and ~1000 brown chromatophore organs that were collected and pooled from five total squid. Yellow chromatophores were pooled from two squid; Red chromatophores were pooled from two squid; and browns were pooled from three.) Spectral count data for Figure S2 was processed using QSpec, which compares samples pairwise in the absence of replicate runs. For the analysis of the granules, pigment-extracted granules, and extracted pigments, the sample size was n = 1 MS/MS, where samples were not separated by color; instead they were collected from whole skin sections across the dorsal and ventral regions of four animals, pooled and distributed at random throughout the extraction and identification studies.
Data exclusions	No data was excluded from analysis.
Replication	All attempts made at replication were successful.
Randomization	Randomization was not required in this study.
Blinding	Blinding was not relevant in our study.

Reporting for specific materials, systems and methods

Materials & experimental systems

n/a	Involved in the study
☒	☐ Unique biological materials
☐	☒ Antibodies
☒	☐ Eukaryotic cell lines
☒	☐ Palaeontology
☐	☒ Animals and other organisms
☒	☐ Human research participants

Methods

n/a	Involved in the study
☒	☐ ChIP-seq
☒	☐ Flow cytometry
☒	☐ MRI-based neuroimaging

Antibodies

Antibodies used	Primary (rabbit) antibody to reflectin was kindly provided by Dr. Daniel Morse [anti-A1/A2] and by Dr. Wendy Goodson. Secondary (goat-anti-rabbit) antibody and a single (Alexa488 or Alexa568) anti-rabbit secondary was used for some experiments; two anti-rabbit secondary antibodies (Alexa405 and Alexa568) were mixed together in others
Validation	Primary anti-reflectin antibodies were validated on other optical organs (e.g., iridophores) in squid skin.

Animals and other organisms

Policy information about studies involving animals; ARRIVE guidelines recommended for reporting animal research

Laboratory animals	The study did not include laboratory animals.
Wild animals	Adult D.pealeii (approximately 6 inches long from mantle to tail) were obtained live in the late Summer and Fall season from the Marine Resources Center at the MBL
Field-collected samples	The study did not include field-collected samples.
